# Functionalised Mn^VI^-nanoparticles: an advanced high-valent magnetic catalyst

**DOI:** 10.1038/srep08636

**Published:** 2015-03-02

**Authors:** Saikat Khamarui, Yasmin Saima, Radha M. Laha, Subhadeep Ghosh, Dilip K. Maiti

**Affiliations:** 1 Department of Chemistry, University of Calcutta, University College of Science, 92 A. P. C. Road, Kolkata-700009, India

## Abstract

We discover Mn^VI^-nanoparticles (NPs) bearing functional groups, high oxidation state, strong electron affinity, unique redox and paramagnetic nature, which opens up a new avenue to catalysis, magnetism and material application. However, its synthesis is challenging and remains unexplored because of associated serious difficulties. A simple benign synthetic strategy is devised to fabricate the high-valent NPs using mild reducing agent bromide, which transformed Mn^VII^ to valuable Mn^VI^-species. The EELS-imaging of individual elements, ESI-MS, XPS and other techniques established its composition as Br(Me_3_SiO)Mn^VI^O_2_. It revealed significantly improved magnetic moment (SQUID) with isotropic hyperfine splitting of six line spectrum (EPR). The high-oxidation state and incorporated-ligands of the metals present on the active surface of the NPs led to development of a general catalytic process for oxidative heterodifunctionalisation to C-C triple bond towards formation of a new O-C/N-C/S-C and C-C coupling *cum* cyclisation to biologically important flavones and their aza- and marcapto-analogues, and valuable enaloxy synthons.

Catalysis is like a key to the major chemical processes of industry and academia^1^,^2^,^3^,^4^,^5^,^6^,^7^. The mid- and low-valent metal catalysts have been dominating for controlling the reactivity and selectivity of organic transformations^1^,^2^,^3^,^4^. Interestingly in the last few years catalysis by high-valent bulk-metals is emerging as an important domain of research^5^,^6^,^7^. We envisioned synthesis of metal-NPs^8^,^9^,^10^,^11^,^12^,^13^ of higher oxidation state possessing incompletely filled d-shell for unique magnetism, highly active surface, strong electron affinity and redox capability and catalytic site preference for outstanding catalytic activity and selectivity. In particular, ligand-modified version of the high-valent metal-NPs is expected to be a versatile catalyst for the oxidative grafting of C-C triple bond through push-pull mechanism towards heterodifunctionalisation^14^ such as O-C/N-C/S-C and C-C coupled fundamental organic transformations *cum* annulation to flavone analogues. However, controlling size and shape of high-valent metal-NPs is a challenge owing to their less stability at higher temperature and other associated problems. The fabrication of even moderately high-valent metal-NPs (e.g. Mn^IV^) was usually achieved by thermal decomposition or through stabilization of co-metal ions^15^,^16^. Thus, we were looking for a straight forward strategy to fabricate nanomaterials of valuable manganese(VI)^17^,^18^ compounds through reduction of inexpensive Mn^VII^-salt (e.g. KMnO_4_) under benign reaction conditions. The designed magnetic Mn^VI^(d^1^)-NPs bearing ligands such as halogen, oxygen and -OR has several advantages during catalytic cycles. For example, ligands are instrumental during catalysis such as activation of bonds, complexation with the precursors and changing oxidation states of metal to construct desired product and regeneration of the valuable catalyst. Easy separation of the magnetic NPs from the post reaction mixture can be performed by simply using an external magnet and it can be reused further with comparable efficiency^19^,^20^,^21^,^22^,^23^,^24^,^25^.

The compounds bearing flavone skeletons are wide spread in Nature and found broad spectrum of applications in medicinal, material and synthetic chemistry^26^,^27^,^28^,^29^,^30^,^31^,^32^,^33^,^34^,^35^,^36^,^37^,^38^,^39^,^40^,^41^,^42^,^43^,^44^,^45^. For instance, the flavone compounds displayed antiulcer, anticancer, antitumor, antinociceptive, anti-inflammatory, antioxidant, antimicrobial, antiviral, antidiabetic and many other pharmacological properties^30^,^31^,^32^,^33^,^34^,^35^,^36^,^37^. Tremendous application of flavone compounds has grown interest among the scientists for their synthesis even in 1898^38^. Intramolecular cyclization of 2-hydroxychalcones, oxidative cyclization of acetophenone, dehydrative cyclization of 1,3-diaryl diketones, cyclization of alkynones, carbon monoxide insertion of iodophenols with terminal alkynes, cycloaddition of α-oxoketene and benzyne, and multistep strategies were developed for their synthesis^39^,^40^,^41^,^42^,^43^,^44^,^45^. The aza-(4-quinilinone)^46^,^47^,^48^ and marcapto-analogues^49^,^50^ of flavone are of much interest due to their bioactivity and their syntheses is especially essential for diverse medicinal applications. Thus, a general strategy for direct construction of substituted flavones and their hetero-atomic analogues is desirable for designing new drugs, innovative materials and synthetic compounds.

## Results

### Design, synthesis and EELS study of the Mn^VI^-NPs

**Figure 1:**
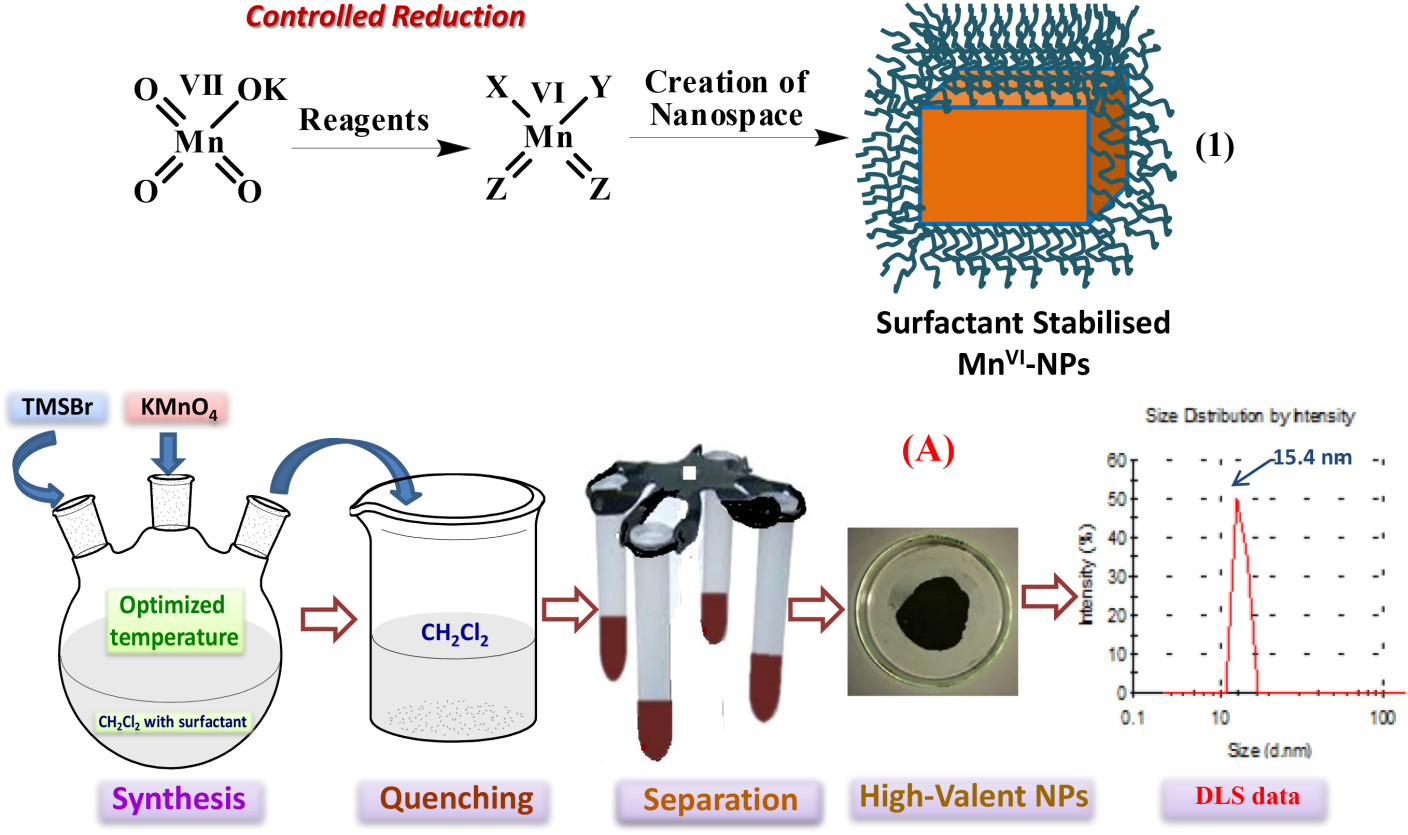
Design and synthesis of Mn^VI^-NPs.

**Figure 2:**
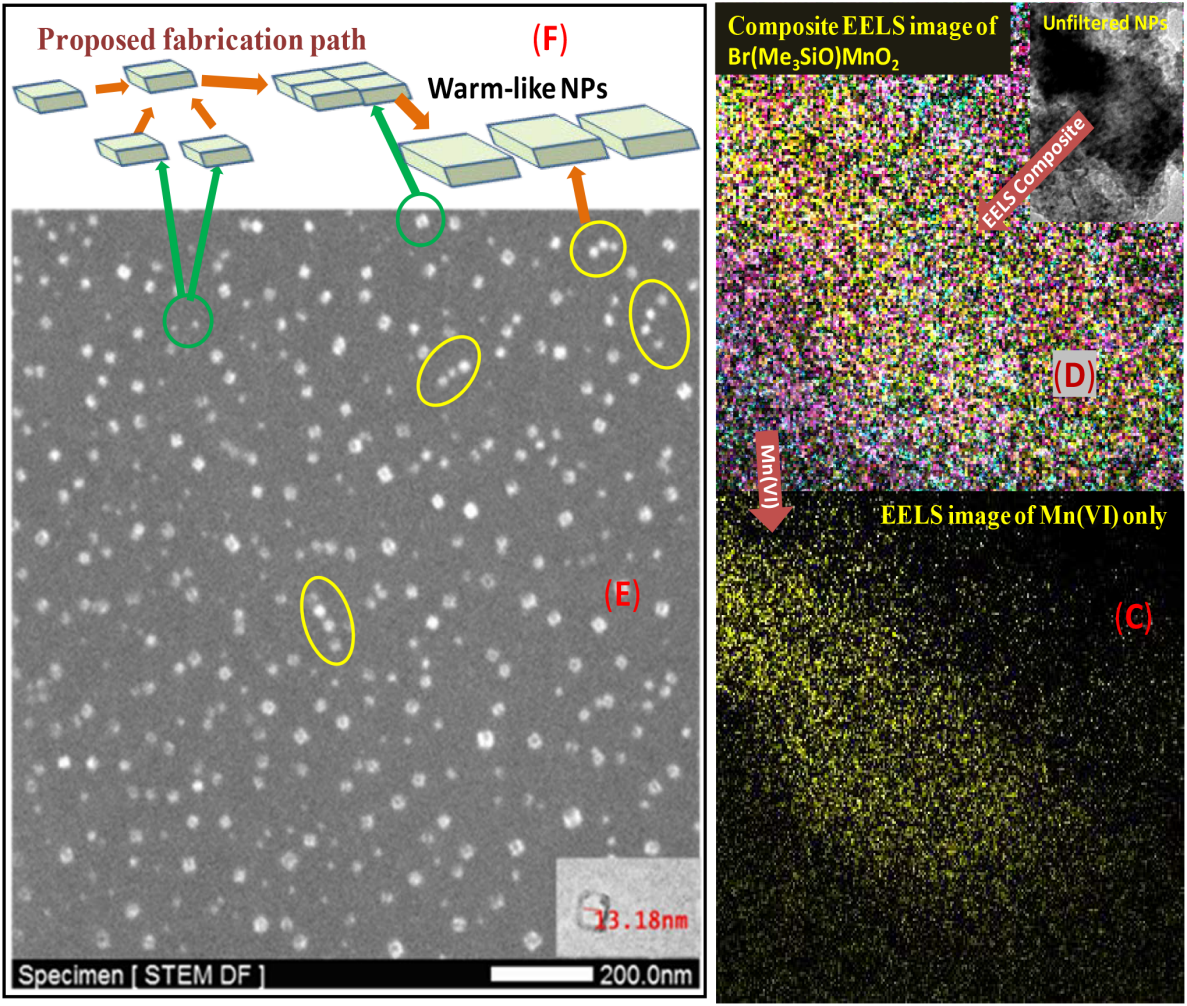
STEM-imaging, EELS elemental mapping (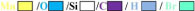) and proposed path of fabrication.

The simple Mn^VII^ salt KMnO_4_ was selected as a precursor to design the XYMn^VI^Z_2_-complex bearing -**X**, -**Y** and **-Z-** groups (eq. 1, Figure 1). We envisioned that the groups such as -I, -Br, -Cl, -OSiMe_3_, -OTf, -O-, -S- etc. possessing good leaving and insertion properties to material will be helpful to accommodate the organic precursors for bond activation around the high-valent metal-sites accomplishing a robust catalysis. After several experiments we found trimethyl silyl bromide as an effective reducing agent to the precursor KMn^VII^O_4_ towards fabrication of Mn^VI^-NPs in CH_2_Cl_2_ containing cetyltrimethyl ammonium bromide (CTAB, 10 mol%) at ambient temperature. The NPs were collected from the surfactant-assembled nanospace after one hour of reductive fabrication of the NPs, precipitation of the nanomaterial by addition of CH_2_Cl_2_, collection through centrifuge and successive washing of the brown colour residue (panel A, Figure 1). The dynamic light scattering measurement of the dilute reaction mixture in CH_2_Cl_2_ revealed maximum population of the NPs at 15.4 nm (panel A, Figure 1). However, the high resolution transmission electron microscope (HR-TEM) imaging of the nanomaterial was inconclusive to determine its morphology. It might be due to rapid damage (panel B, Figure 2) on their organic component-bearing surface by the strong electron-beam of TEM, high reactivity of the metal component of highly oxidation state and/or weak signal generation from the thin nanomaterial. Recently, scanning transmission electron microscope - Electron Energy Loss Spectroscopy (STEM- EELS) is emerging as a powerful tool for elemental mapping of nanomaterials^51^,^52^,^53^,^54^. Our EELS study for the Mn^VI^-NPs revealed presence of worm-like NPs (panel C, Mn only). The mapping of all atoms such as Mn, O, Si, C, Br and H was established by the EELS study. The composite EELS image displayed in panel D and other individual images are supplied in the supporting information. Worm like structure has also been confirmed using AFM study.

To understand the mechanism of fabrication process of the nanomaterials we analyzed a dispersed monolayer material, which was taken out from the ongoing reaction. Gratifyingly it revealed presence of small cube-like NPs along with relatively larger cube-like structure (green circles, panel E) as well as small worm-like structures (yellow circle), which was imaged on dark field (DF) scanning transmission electron microscope. Combination of four small NPs to form the actual unit cell is visible in the panel B with dark-red circle which ultimately construct the worm-like thin NPs. Interestingly the smallest one can couple among four units to form larger cubical nanomaterial which also can arrange nicely between three to four units to form worm-like NPs (~13 nm). The proposed scheme is presented in the panel F. The reaction mixtures exhibit two UV-vis absorption peaks at 514.1 and 534.9 nm, which also support presence of Mn^VI^-NPs of two different size in the reaction mixture.

### Elucidation of structure of the Mn^VI^-NPs

**Figure 3:**
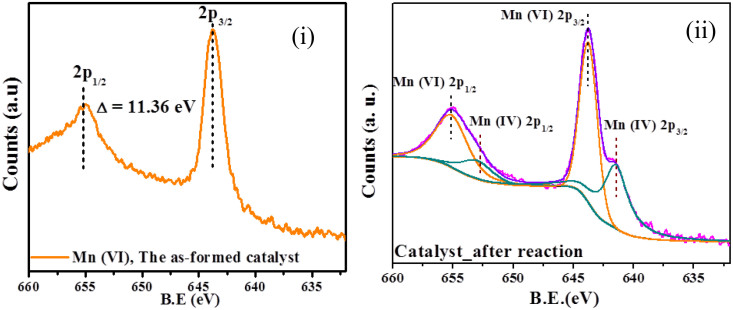
XPS spectra of the fabricated Mn-NPs (i) and recovered NPs from the reaction mixture (ii).

The orange line in the panel (i), Figure 3 corresponds to the XPS spectrum of the as-synthesized Mn-NPs, which indicates presence of manganese in the +6 oxidation state^55^. The pink line in panel (ii), Figure 3 represents the XPS spectrum of the recovered manganese catalyst from the reaction mixture. The deconvolution graph (violet line) was found after fitting the data in XPS software. It showed presence of a mixture of Mn^VI^ and Mn^IV^ in the recovered material, which matches with the standard curve for Mn^VI^ (orange line) and Mn^IV^ (green line). The powder X-ray diffraction peaks (2*θ*) of the solid Mn-NPs appeared at 28.1°, 40.2°, 49.8°, 58.1°, 66.1° and 73.1°. Electronspray ionization-mass spectrometry (ESI-MS) of the NPs is performed in CH_3_CN medium and the appearance of ESI-MS-generated peak at 254.8885 dalton established the composition Mn^VI^-NPs as Br(Me_3_SiO)MnO_2_. Presence of (CH_3_)_3_SiOMn group was verified by measurement of FTIR spectroscopy of the nanomaterial, which revealed appearance of FTIR stretching vibration for C-H at 2922 cm^−1^, Si-O at 1393 cm^−1^ and Mn^VI^-O broad peaks around 511 cm^−1^. The peak at δ 1.46 in the solid state^1^H-NMR confirmed the presence of methyl group of –OSi(C*H*_3_)_3_ in the functionalized NPs. All other spectra are available in the supporting information.

### Unusual nanoscale magnetism of the high-valen Mn^VI^-NPs

**Figure 4:**
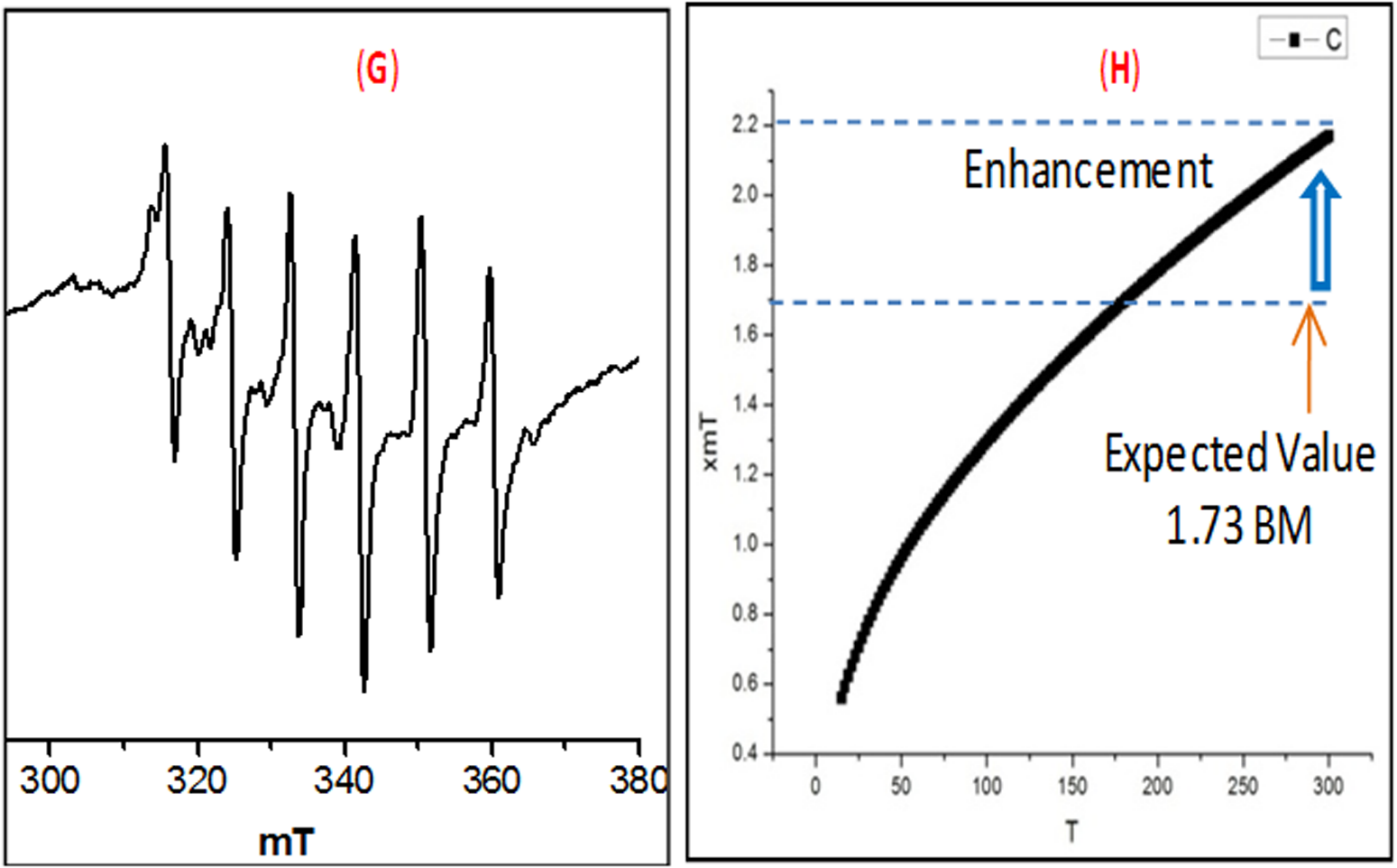
EPR and SQUID diagram of the Mn^VI^-NPs.

Interestingly, spin only magnetic moment of Mn^VI^-complex in nano-state is found to be 2.2 BM at room temperature, which is unusually high relative to the expected value (~1.73 BM) of bulk-Mn^VI^ compound bearing 3d^1^ electron. The X-band EPR spectrum (panel G, Figure 4) of the powdered nanomaterial at −78°C (liquid N_2_) showed a significantly improved isotropic hyperfine splitting^55^ (Mn; *I* = 5/2) of six line spectrum (g = 1.99836). Earlier, Wieghardt *et al.* reported a six line EPR spectrum (g ~ 2.0) of [(cyclam)Mn^VI^(N)(NCCH_3_)]^3+^-complex^56^. The higher value of magnetic moment of the small nanomaterial and hyperfine splitting in EPR spectrum may be attributed due to their exceptionally high oxidation state and existence of magnetic vector in an unidirectional^56^ fashion. It led us to execute the temperature-dependent SQUID measurement^57^ of the nanomaterial, which also revealed (panel H) higher magnetic moment (2.2 BM). It clearly indicates the special arrangement (F, Figure 1) of the tiny high- valent magnetic NPs (vector) which eventually added the vector towards significant enhancement of the magnetic moment.

### Discovery of C-X/C-C coupled annulation catalysis

**Figure 5:**
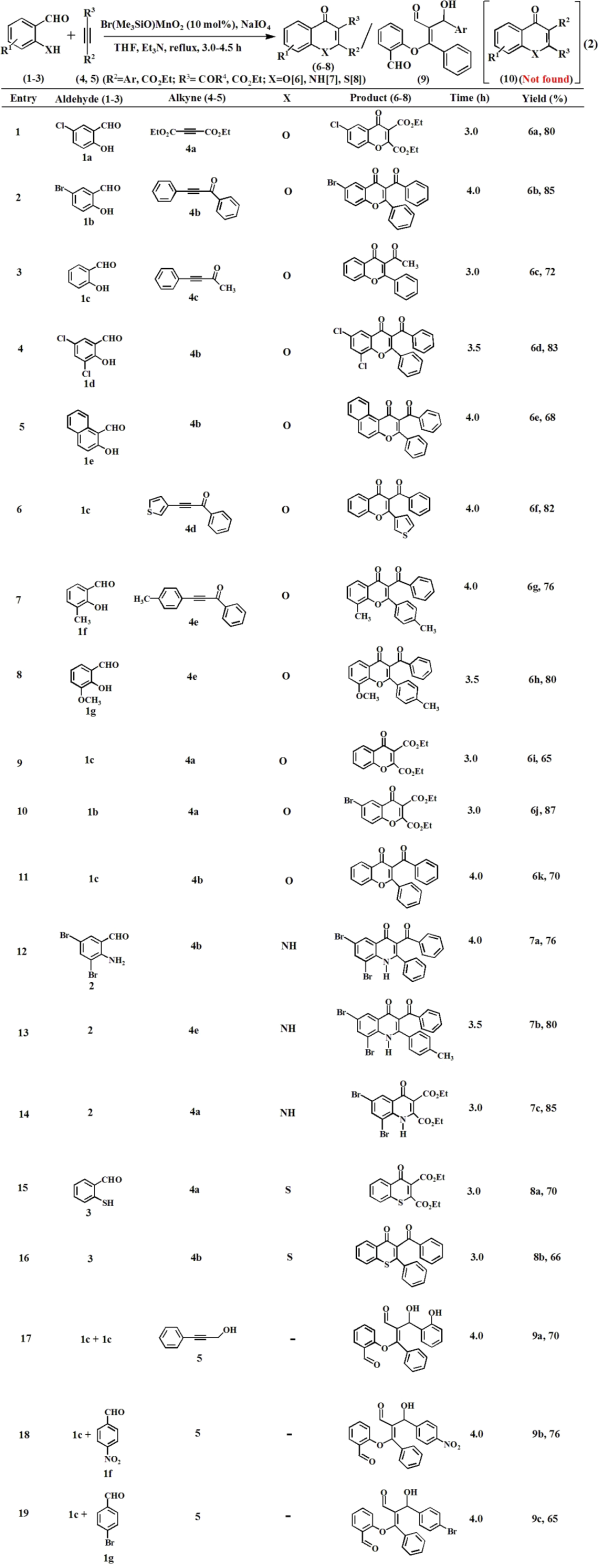
Direct construction of flavone analogues and 3-oxyenals.

Design, synthesis and development of new catalytic activity of a material are of intense interest to the chemical science community because the catalyst offers novel reactivity and selectivity towards synthesis of valuable compounds. An annulation reaction that can selectively execute O-C/C-C coupling through grafting of C-C triple bond is a very promising strategy towards direct construction of functionalised analogues of natural product flavones (**6**, eq. 2, Figure 5). Herein, we for the first time synthesize Mn^VI^-NPs, developed outstanding catalytic activity for O-C/N-C/S-C^20^,^25^ and C-C coupled annulation to flavone skeletons (**6**–**8**) and enaloxy synthons (**9**), easy recovery of the magnetic catalyst from the post reaction mixture by using external magnet and successfully recycled, and several facets of catalysis for C–C triple bond functionalisation reactions. We first observed the oxidative coupling between functionalized salicylaldehyde derivative (**1a**, entry 1) and a deactivated alkyne (**2a**) to undergo annulation reaction at ambient temperature in THF towards direct construction of valuable flavones (**6a**, 20% yield) in presence of catalytic amount (10 mol%) of the Mn^VI^-NPs, NaIO_4_ (1.1 mmol) and triethylamine. Gratifyingly the reaction was complete in 4 h and yield (80%) as well as catalyst loading (10 mol%) were improved by rising the reaction temperature to 70°C. NaIO_4_ was needed as a stoichiometric oxidant for the O-C and C-C coupled annulation process because the yield was drastically reduced (7%) in its absence under the optimized conditions. On carrying out the reaction using bulk-Mn^VI^ compound as a catalyst conversion of the starting material was very poor (~40% conversion) even after prolong heating (~24 h). Similarly, the product **6a** was not found in absence of the catalyst. It indicates that surface of the functionalized Mn^VI^-NPs is highly active towards binding the precursors (**1a** and **4a**) which led to grafting of the triple bond with rapid annulation. This catalytic strategy was helpful for synthesis of a large number of flavones (**6a**–**k**, entries 1–11) within 3–4 h by varying the starting ingredients. Methyl, halogen, methoxy etc. substituted aromatic rings, heterocycles, carbonyl, ester functionalities were tolerated under the above optimized reaction conditions. The feasibility of the reaction was also checked with highly deactivated alkyne such as diethyl acetylene dicarboxylate, which produced the desired flavones (**6a**, **6i** and **6j**). Interestingly, the functionalized surfaces of high-valent-NPs are very much site-selective for binding the precursors which led to complete regioselective annulation to compound **6**. The other possible regioisomer **10** (eq. 2) was not found using unsymmetrical alkynes (entries 2–8). The nitrogen analogues of flavones i.e. 4-quinolinones (**7a–c**, entries 12–14) and marcapto-flavones (**8a**,**b**, entries 15–16) were also been synthesized using 2-aminobenzaldehyde and 2-marcaptobenzaldehyde respectively under the similar reaction conditions. Thus, the newly synthesized high-valent Mn^VI^-NPs were found as robust catalyst for developing a rapid and general strategy to bioactive flavone analogues with outstanding regioselectivity and high yield. Surprisingly on replacement of carbonyl group by alcoholic -OH in the triple bond the C-C coupled annulation was completely unsuccessful to afford corresponding flavone (**6**, entry 17). In turn, it underwent C-C coupling with another aldehyde functionality to afford highly functionalised valuable enaloxy synthons (**9a**–**c**, entries 17–19) in 4 h with 65–76% yield.

### The possible role of the high-valent Mn^VI^-NPs in the robust catalysis

**Figure 6:**
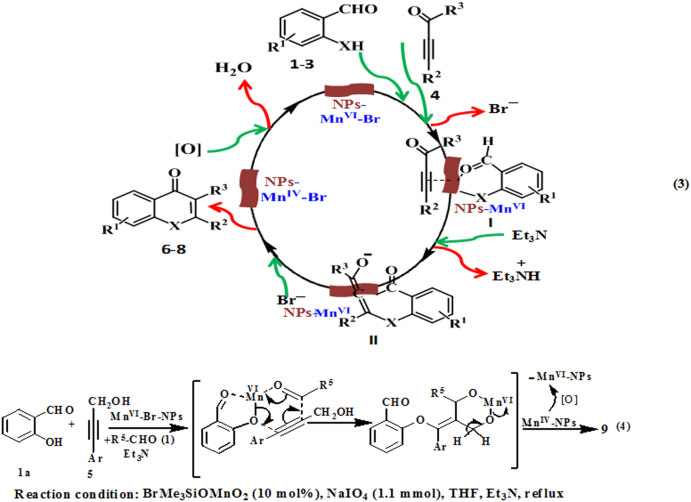
Possible annulation catalytic cycle for flavones and enaloxy synthons.

Exact mechanism of the catalytic process is unknown to us. However, high oxidation state of the Mn^VI^-NPs, catalytic site-preference, metal-bearing good leaving groups, and highly active nanoscale-surface with strong electro affinity of the NPs lead to activation of the precursor aldehyde (**1**–**3**) and alkyne (**4**) through formation of an assembly (**I**, eq. 3, Figure 6) involving C-C triple bond, carbonyl oxygen and -XH. In this step bromide was lost from the NPs and Mn-X bond was formed. The reactive intermediated **II** is expected to form by deprotonation of aldehyde through -CO-*H* activation and regioselective nucleophilic addition of -X from C_3_ of the alkyne (**4**) to generate O-C/N-C/S-C bond. During optimization of the reaction we observed that presence of base NEt_3_ was essential in the catalysis process, which might be needed to abstract aldehyde-sp^2^C-*H* for transforming **I** to **II**. The desired product **6**, **7** or **8** was produced *via* oxidative C-C coupling of the allene-like intermediate(**II**) with insertion of bromide and release of reduced Mn^IV^-modified NPs. Trace amount of Mn^IV^ along with Mn^VI^ was detected *via* XPS analysis of the recovered catalyst from the ongoing reaction mixture. (SI) Catalytic cycle is maintained through regeneration of the Mn^VI^-NPs at the surface by the stoichiometric oxidant sodium metaperiodate (NaIO_4_) under the reaction conditions. To understand whether the involvement of carbonyl oxygen of the alkyne with the high-valent Mn^VI^-NPs is essential for the oxidative C-C coupled annulation process we used its reduced form i.e. propargyl alcohol (**5**) under the similar reaction conditions. However, corresponding oxidative C-C coupled annulation product (**6**, Figure 5) involving aldehyde group of salicylaldehyde (**1**) was not found from the post reaction mixture. It supports our proposed reaction pathway, which passes through initial coordination of the carbonyl oxygen of **4** (**I**) with Mn^VI^-NPs. Interestingly the high-valent-NPs completely changed the O-C/C-C coupling reaction path and it was joined to another molecule of salicylaldehyde with simultaneous oxidation of -CH_2_OH of **5** to aldehyde affording highly functionalized synthon **9a** (entry 17, Figure 5). The catalytic three component coupling process was also successfully carried out in presence of other aldehydes (**9b**,**c**, entries 18,19). It indicates that oxidation took place after the Mn^VI^-NPs catalyzed cascade type of O-C and C-C coupling, which is expected to proceed through formation of two successive six member transition states **III** and **IV** (eq. 4, Figure 6). XPS studies (Figure 3) showed that recovered catalyst was the mixture of Mn^IV^ and Mn^VI^, which were very difficult to separate for characterization. It supports our proposed reaction pathway, which passes through transformation of intermediate **II** to desired product (**6**–**8**) with generation of Mn^IV^-species (Figure 6). Interestingly we observed in our experiments using the recovered catalyst that it was equally efficient to as-fabricated Mn-NPs in presence of NaIO_4_ due to transformation of Mn^IV^ to Mn^VI^ under the reaction conditions.

## Discussion

**Figure 7:**
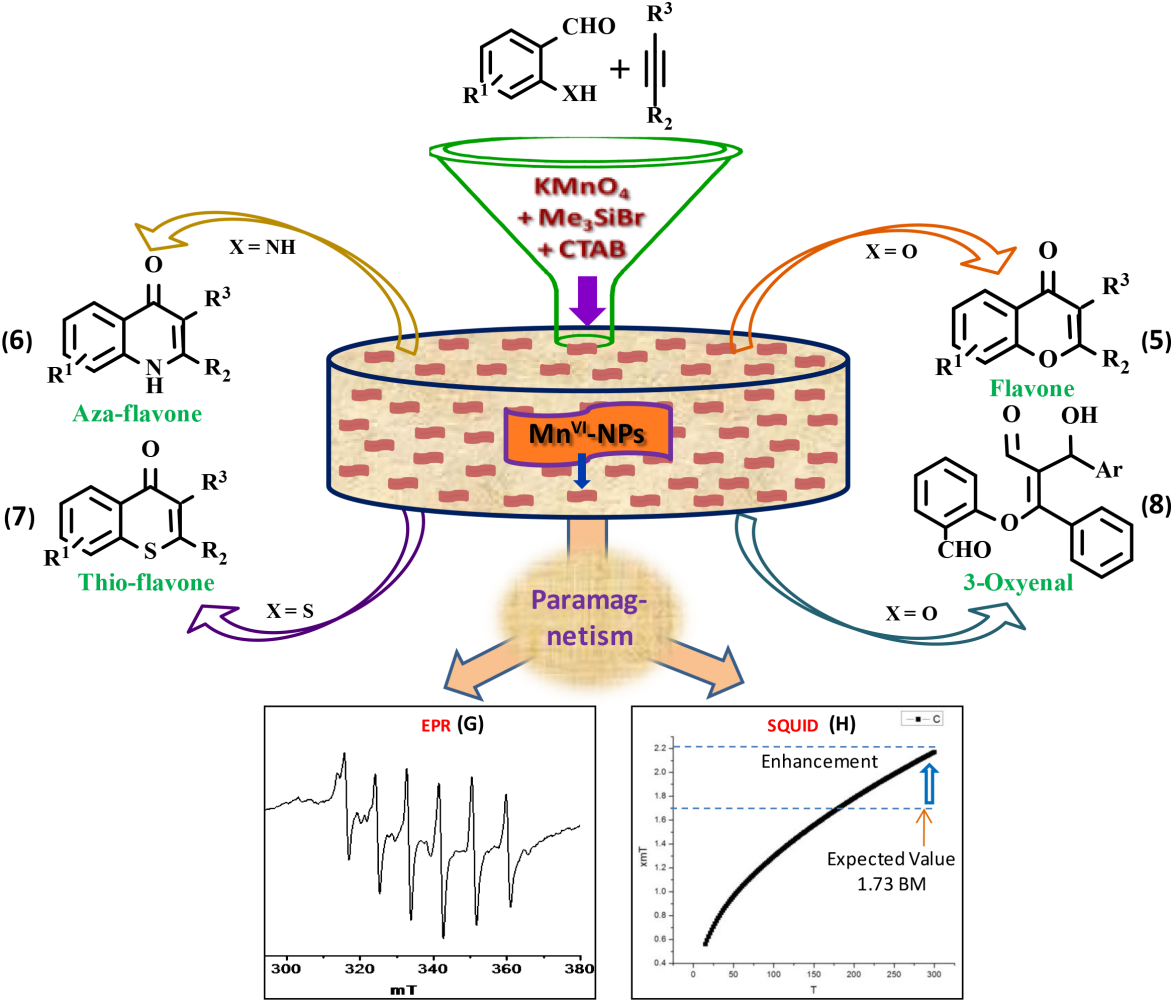
High-valent Mn^VI^-NPs and its innovative properties.

In this article, we have demonstrated an innovative design and fabrication of a functionalised high-valent nanomaterial through formation of a new Mn^VI^-compound under benign reaction conditions. Herein, we report an unprecedented reductive transformation of readily available Mn^VII^-salt KMnO_4_ to valuable Mn^VI^-species using Me_3_SiBr as a mild reducing agent. The Mn^VI^-NPs bearing uncommon six oxidation state, exchangeable ligands, catalytic-site preference possibility, strong electron affinity, unique redox and magnetic property is the potential candidate for unique magnetism, material and catalysis. However, fabrication of well defined high-valent metal-NPs is a difficult job because of its higher oxidation state, reactivity and less stability under the heating conditions which is usually required during synthesis of a nanomaterial. Herein, a benign strategy is devised for fabrication of the high-valent metal-NPs of uniform size and shape through in situ generation of the ingredient and creation of nanospace utilizing inexpensive KMnO_4_, Me_3_SiBr and CTAB (Figure 7). The modern EELS imaging technique is used for mapping of individual elements present in the nanomaterial and it revealed formation of worm-like structure. The possible mechanism of unidirectional packing of the nanomaterial is also predicted (panel F, Figure 2) with the experimental evidence. The primary formula of the NPs was established as Br(Me_3_SiO)Mn^VI^O_2_. A significantly improved magnetic property was observed in the X-band ESR spectroscopy of the NPs with isotropic hyperfine splitting of six line spectrum (panel G, Figure 7). It also showed higher magnetic moment with respect to common Mn^VI^-compounds (d^1^), which is comparable to the temperature dependant SQUID data (panel H). As expected the high-oxidation state and incorporated-ligands of the metals present on the active surface of the NPs was crucial to develop a robust catalytic process for oxidative heterodifunctionalisation of C-C triple bond towards formation of a new O-C/C-C coupling *cum* cyclisation to afford a wide range of biologically important flavone (**6a**–**k**, eq. 5, Figure 7) compounds. Herein, we have introduced a general strategy for direct construction of the bioactive flavone with outstanding regioselectivity and its analogues such as azaflavones (**7a**–**c**, eq. 6) and marcaptoflavones (**8a**,**b**, eq. 7), and a valuable 3-oxyenal synthon (**9a**–**c**, eq. 8) involving and N-C/S-C/O-C and C-C coupling, which is quite important to synthetic and medicinal chemistry - the most active area of contemporary research. We believe that the intriguing and inspiring strategy for fabrication of the functionalized high-valent metal-NPs, its unique magnetic property and diverse catalytic activity will open up a new avenue in science and technology.

## Methods

### Synthesis of Mn^VI^-NPs

In a 100 mL round bottomed flask, CTAB (364 mg, 1 mmol) and CH_2_Cl_2_ (36.4 mL) were taken together and stirred magnetically for 5 min. KMnO_4_ (158 mg, 1 mmol) was added into the solution and stirring was continued. Me_3_SiBr (306 mg, 2 mmol) was added drop wise at 0°C and content of the reaction mixture was stirred for 45 min. Finally the reaction mixture was poured into 200 mL of CH_2_Cl_2_ and centrifuged, washed with CH_2_Cl_2_ (5 × 20 mL) and dried under reduced pressure at ambient temperature to afford Mn^VI^-NPs as a brown solid material (yield: 52%; 138 mg, 0.52 mmol). The characterization data of the new nanomaterial such as FTIR, NMR, ESI-MS, powder XRD, UV-vis, AFM, FESEM-EDS for elements, EELS elemental mapping and the important spectra are incorporated in the supporting information.

### Synthesis of flavones

To a solution of salicylaldehyde or 2-amino-3,5-dibromo-benzaldehyde or thio-salicylaldehyde (1.0 mmol) in THF (20 mL) triethylamine (1.1 mmol, 111 mg) was added drop wise under stirring condition. The propargyl ketone (1.0 mmol) was added to it. The Mn^VI^-NPs (10 mol%) and sodium periodate (1.1 mmol, 235 mg) were added and allowed to reflux at 70°C for 3–4 h. The reaction was monitored by thin layer chromatography (TLC). The solvent of the post reaction mixture was removed under reduced pressure at room temperature and extracted with EtOAc (2 × 20 mL). The combined organic layer was washed successively with saturated sodium bicarbonate solution (1 × 10 mL) and brine (3 × 10 mL). It was dried over anhydrous Na_2_SO_4_, filtered and evaporated to dryness in a rotary evaporator under reduced pressure at room temperature. Thus, the reaction with 5-chlorosalicylaldehyde (1.0 mmol, 156 mg) and diethylacetylenedicarboxylate (1.0 mmol, 170 mg) afforded 6-chloro-4-oxo-4*H*-chromene-2,3-dicarboxylic acid diethyl ester (**6a**) which was isolated after purification by column chromatography on silica gel (60–120 mesh) using ethyl acetate-petroleum ether (1:9, v/v) as an eluent to afford 80% (259 mg, 0.70 mmol) yield. The synthesized flavones derivatives (**6a–k**), azaflavones (**7a–c**) and marcaptoflavones (**8a**,**b**) were characterised by recording NMR (^13^H and ^13^C), FTIR and Mass (HR-MS) spectra. The characterization data of the all new compounds and their spectra are reported in the supporting information.

## Author Contributions

Major work was done by S.K. and Y.S. S.K., Y.S., R.M.L., S.G. and K.M. were contributed in synthesizing and characterizing the molecules, manuscript preparation, discussion on chemistry involved, and commenting on the manuscript.

## Supplementary Material

Supplementary Information Functionalised MnVI-nanoparticles: an advanced high-valent magnetic catalyst
