# Supplementary material for: Functionalised MnVI-nanoparticles: an advanced high-valent magnetic catalyst
Source: Sci Rep. 2015 Mar 2;5:8636. doi: 10.1038/srep08636 (PMC4345317; doi:10.1038/srep08636)

**Supporting Information**

Functionalised Mn^VI^-nanoparticles: an advanced high-valent magnetic catalyst

*Saikat Khamarui, Yasmin Saima, Radha M. Laha, Subhadeep Ghosh and Dilip K. Maiti**

Department of Chemistry, University of Calcutta, University College of Science,

92, A. P. C. Road, Kolkata-700009, India.

*Corresponding author. Fax: 91-33-2351 9755, Tel: 91-33-2350 1014

*dkmchem@caluniv.ac.in*

Serial No. Content Page Numbers

1. Materials and methods S-2

2. Procedure for the fabrication of the Mn^VI^-NPs S-2

3. Characterization data of the synthesized Mn^VI^-NPs S-2

3.1. FTIR data of the Mn^VI^-NPs S-2

3.2. Identification of elements of the Mn^VI^-NPs by EDS technique (Fig. 1) S-2

3.3. Atomic Force Microscope (AFM) image of the Mn^VI^-NPs (Fig. 2) S-3

3.4. Powder XRD of the NPs (Fig. 3) S-3

3.5. UV-Visible spectrum of the NPs (Fig. 4) S-4

3.6. EELS images of the component present in the NPs (Fig. 5) S-4

3.7. ESI-MS spectrum of the NPs (Fig. 6) S-5

3.8. Solid state 1H-NMR of the NPs (Fig. 7) S-6

4. General procedure for the synthesis of flavones, aza-flavones and

marcapto-flavones S-7

5. Characterization data of the synthesized flavones **(6a-k)** S-7

6. Characterization data of aza-flavones **(7a-c)** S-12

7. Characterization data of thio-flavones (**8a**, **b**) S-13

8. General procedure of oxidative coupling toward 3-oxyenals S-14

9. Characterization data of 3-oxyenals **(9a-c)** S-14

10. References S-15

11. ESI-MS spectra of compounds S-16

12. ^1^H & ^13^C NMR Spectra of Compounds **6a**-**k**, **7a-c**, **8a**, **b** & **9a-c** S-19

**1. Materials and methods.** All solvents were dried by standard methods. Unless otherwise specified, chemicals were purchased from commercial suppliers and used without further purification. Column chromatography was performed on silica gel (60-120 mesh). TLC was done on glass sheets pre-coated with silica gel (with binder, 300 meshes, Merck). Scanning transmission electron microscopy (TEM) images and electron energy loss spectrum (EELS) were taken using an ultra-high resolution field emission gun transmission electron microscope (UHR-FEG TEM, JEM-2100F, JEOL, Japan) operating at 200 kV. The ^1^H- and ^13^C-NMR spectra were taken in CDCl_3_ with TMS as an internal standard on Bruker Supercon NMR spectrometer (Model: AV 300 Digital). The chemical shifts were reported as δ values (ppm) relative to tetramethylsilane. Proton multiplicities are represented as s (singlet), d (doublet), dd (double doublet), t (triplet), q (quartet), and m (multiplet). Infrared spectra were recorded on FTIR spectrometer in KBr pellets and in NaCl cell (liquid sample) on a Perkin-Elmer RXI-FTIR spectrophotometer. Melting points of the samples were determined with a Fisher-John melting point apparatus and were uncorrected. HR-MS data were acquired by electron spray ionization technique on a Q-tof-micro quadriple mass spectrophotometer (Bruker).

**2. Procedure for the fabrication of the Mn^VI^-NPs.** In a 100 mL round bottomed flask, CTAB (364 mg, 1 mmol) and CH_2_Cl_2_ (36.4 mL) were taken together and stirred magnetically for 5 min. KMnO_4_ (158 mg, 1 mmol) was added into the solution and stirring was continued. Me_3_SiBr (306 mg, 2 mmol) was added drop wise at 0 °C and content of the reaction mixture was stirred for 45 min. Finally the reaction mixture was poured into 200 mL of CH_2_Cl_2_ and centrifuged, washed with CH_2_Cl_2_ (5 x 20 mL) and dried under reduced pressure at ambient temperature to afford Mn^VI^-NPs as a brown solid material (yield: 52%; 138 mg, 0.52 mmol).

**3. Characterization data of the synthesized Mn^VI^-NPs**

**3.1. FTIR data of the Mn^VI^-NPs (KBr, Cm^-1^):** 453, 511, 754, 1393, 1522, 2922.

**3.2. Identification of elements of the Mn^VI^-NPs by EDS technique.** The following energy dispersive spectrum (EDS) was studied in a field emission scanning electron microscope (FESEM, JEOL, JSM 6700F).


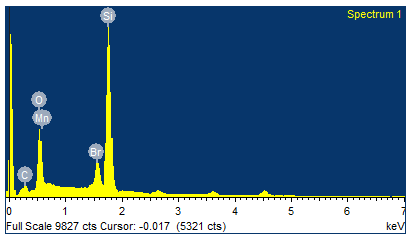


**Figure 1**

**3.3.** **Atomic Force Microscope (AFM) image of the Mn^VI^-NPs.** AFM image was captured in Agilent-5100.

**
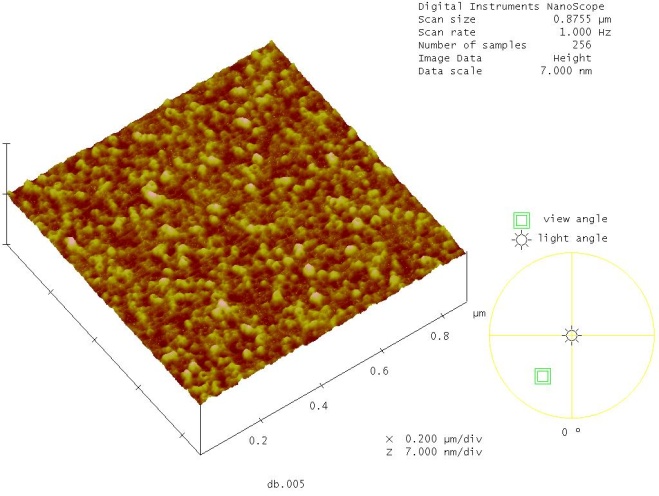
**

**Figure 2**

**3.4.** **Powder XRD of the NPs.** The powder X-ray diffraction pattern was recorded on a Bruker D-8 advance diffractometer operated at 40 kV voltage and 40 mA current and calibrated with a standard silicon sample, using Ni-filtered Cu-Kα (α = 0.15406 nm) radiation.

**
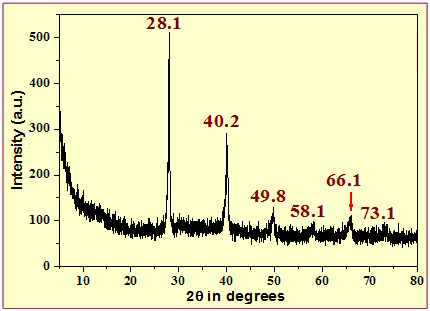
**

**Figure 3**

**3.5.** **UV-visible spectrum of the NPs in CH_2_Cl_2_.** Electronic absorption spectrum of the high-valent NPs was recorded using a Hitachi U-3501 spectrophotometer.

**Figure 4**

**3.6.** **EELS images of the individual components present in the NPs.** EELS of the synthesized Me_3_SiOMnO_2_Br-NPs were performed using JEOL TEM machine. Individual and composite elemental mapping of Me_3_SiOMnO_2_Br-NPs are displayed in the followings.

**Figure 5**

**3.7.** **ESI-MS spectrum of the NPs.** The exact mass for the metal NPs of formula C_3_H_9_BrMnO_3_Si is 254.8885 and found as 254.8857.

**Figure 6**

**3.8.** **Solid state 1H-NMR of the NPs.** The presence of organic component OSi(C***H***_3_)_3_ in the NPs was also confirmed in the solid sate ^1^H NMR of nanomaterial which appeared at δ1.469.

**
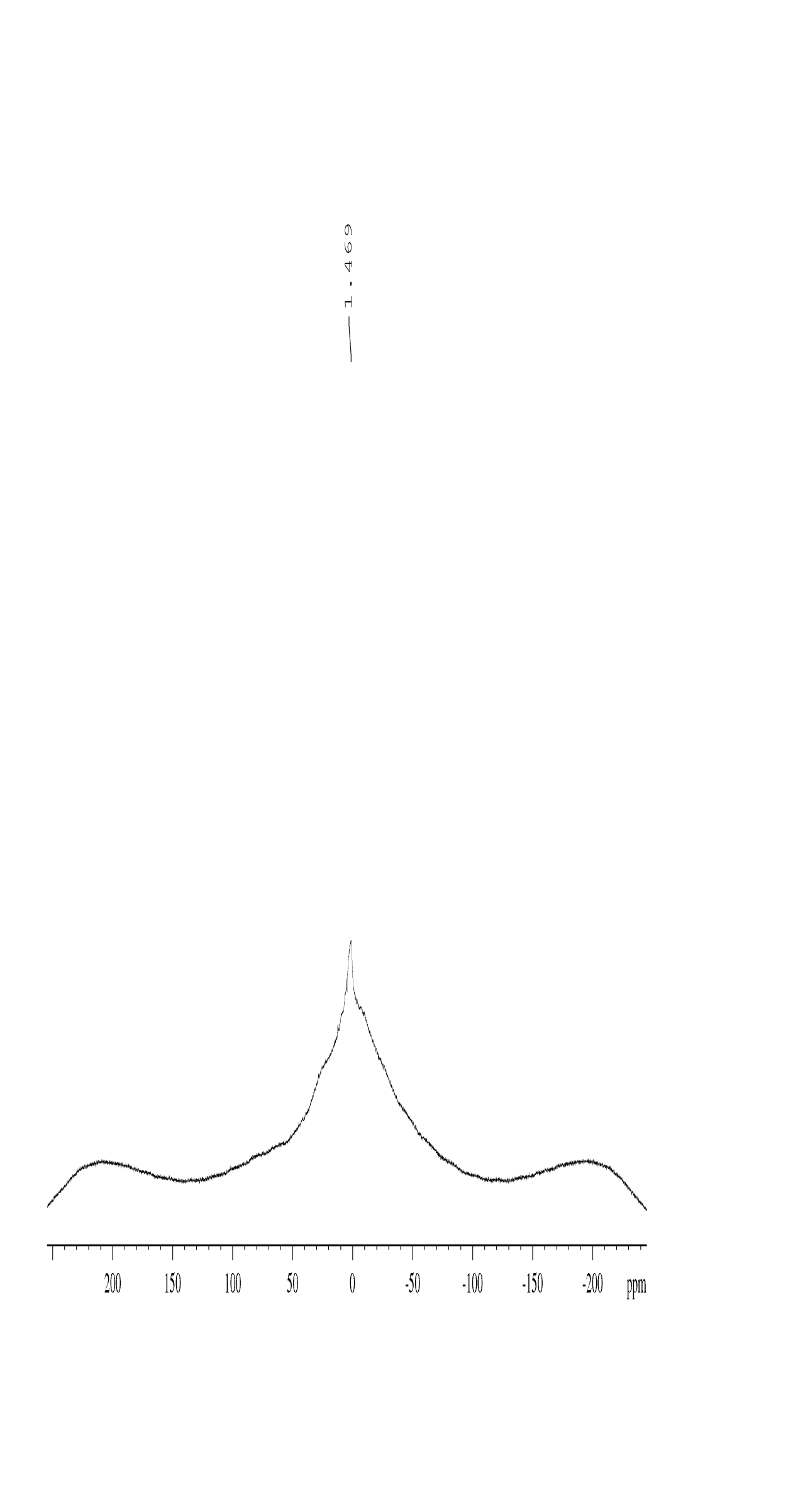
**

**Figure 7**

**4. General procedure for the synthesis of flavones, aza-flavones and marcapto-flavones**

To a solution of salicylaldehyde or 2-amino-3,5-dibromo-benzaldehyde or thio-salicylaldehyde (1.0 mmol) in THF (20 mL) triethylamine (1.1 mmol, 111 mg) was added drop wise under stirring condition. The propargyl ketone (1.0 mmol) was added to it. The Mn^VI^-NPs (10 mol %) and sodium periodate (1.1 mmol, 235 mg) were added and allowed to reflux at 70 ^o^C for 3-4 h. The reaction was monitored by thin layer chromatography (TLC). The solvent of the post reaction mixture was removed under reduced pressure at room temperature and extracted with EtOAc (2x20 mL). The combined organic layer was washed successively with saturated sodium bicarbonate solution (1x10 mL) and brine (3x10 mL). It was dried over anhydrous Na_2_SO_4_, filtered and evaporated to dryness in a rotary evaporator under reduced pressure at room temperature. Thus, the reaction with 5-chloro-salicylaldehyde (1.0 mmol, 156 mg) and diethylacetylenedicarboxylate (1.0 mmol, 170 mg) afforded 6-chloro-4-oxo-4*H*-chromene-2,3-dicarboxylic acid diethyl ester (**6a**) which was isolated after purification by column chromatography on silica gel (60-120 mesh) using ethyl acetate-petroleum ether (1:9, v/v) as an eluent to afford 80% (259 mg, 0.70 mmol) yield. The synthesized flavones derivatives (**6a-k**), aza-flavones (**7a-c**) and thio-flavones (**8a,b**) were characterized by recording NMR (^1^H and ^13^C), FTIR and Mass (HR-MS) spectra. The characterisation data of the all new compounds and their spectra are reported in the supporting information. Further, the structure of **6a** was confirmed by comparing of the reported spectroscopic data of the compound.^1^

**5. Characterization data of the synthesized flavones (6a-k)**

5.1. **6-Chloro-4-oxo-4*H*-chromene-2,3-dicarboxylic acid diethyl ester (6a)**

Yield: 80% (259 mg, 0.80 mmol).

Characteristic: Yellow oil.

^1^H NMR (400 MHz, CDCl_3_): δ 1.25-1.34 (6H, dt, *J* = 7.6 Hz), 4.24-4.41 (4H, dq, *J* = 7.6 Hz), 6.93 (1H, d, *J* = 9.6 Hz), 7.29-7.31 (1H, m), 7.71 (1H, s).

^13^C NMR (100 MHz, CDCl_3_): δ 13.9, 14.2, 61.5, 63.7, 118.4, 119.5, 122.8, 127.4, 128.3, 132.2, 149.9, 156.2, 163.7, 169.0, 176.6.

FTIR (neat, cm^-1^): 1267, 1453, 1648, 1715, 1753.

HR-MS (*m*/*z*) for C_15_H_13_ClO_6_ (M^+^): Calculated 324.0401, found 324.0406 (One of the major peaks).

5.2. **3-Benzoyl-7-bromo-2-phenylchromen-4-one (6b)**

Yield: 85% (344 mg, 0.85 mmol).

Characteristic: Colourless solid.

Melting point: 126-129 ^◦^C

^1^H NMR (300 MHz, CDCl_3_): δ 7.33-7.59 (7H, m), 7.64-7.67 (2H, m), 7.86 (1H, dd, *J* = 9.0, 2.4 Hz), 7.90-7.93 (2H, m), 8.38 (1H, d, *J* = 2.4 Hz).

^13^C NMR (75 MHz, CDCl_3_): δ 119.1, 120.1, 122.6, 124.6, 127.1, 128.2, 128.5, 128.7, 128.8, 129.4, 131.3, 131.7, 133.9, 136.8, 137.3, 154.8, 162.7, 175.1, 193.0.

FTIR (KBr, cm^-1^): 1378, 1466, 1640, 1675, 1709.

HR-MS (*m*/*z*) for C_22_H_13_BrO_3_ (M^+^): Calculated 404.0048, found 404.0045 (One of the major peaks).

5.3. **3-Acetyl-2-phenylchromen-4-one (6c)**

Yield: 72% (190 mg, 0.72 mmol).

Characteristic: Yellow thick liquid.

^1^H NMR (300 MHz, CDCl_3_): δ 2.31 (3H, s), 7.18-7.27 (3H, m), 7.28-7.62 (5H, m), 7.63-7.67 (1H, m).

^13^C NMR (75 MHz, CDCl_3_): δ 21.7, 122.1, 126.3, 128.2, 128.4, 128.6, 128.9, 129.2, 129.7, 130.5, 132.7, 134.9, 138.2, 156.5, 162.8, 175.9, 190.5.

FTIR (neat, cm^-1^): 1156, 1268, 1372, 1462, 1644, 1670, 1715.

HR-MS (*m*/*z*) for C_17_H_12_O_3_ (M^+^): Calculated 264.0786, found 264.0789.

5.4. **3-Benzoyl-5,7-dichloro-2-phenylchromen-4-one (6d)**

Yield: 83% (328 mg, 0.83 mmol).

Characteristic: Colourless solid.

Melting point: 146-148 ^◦^C

^1^H NMR (300 MHz, CDCl_3_): δ 7.20 (1H, s), 7.10 (1H, s), 7.18-7.37 (3H, m), 7.40-7.51 (3H, m), 7.56-7.68 (2H, m), 7.74 (2H, d, *J* = 7.8 Hz).

^13^C NMR (100 MHz, CDCl_3_): δ 123.1, 126.2, 126.5, 126.8, 127.3, 128.3, 128.8, 129.1, 129.4, 129.8, 129.9, 132.2, 132.3, 133.7, 134.5, 136.8, 141.6, 146.9, 157.0, 162.1, 176.3, 194.8.

FTIR (KBr, cm^-1^): 1144, 1259, 1314, 1436, 1551, 1595, 1651, 1706.

HR-MS (*m*/*z*) for C_22_H_12_Cl_2_O_3_ (M^+^): Calculated 394.0163, found 394.0161 (One of the major peaks).

5.5. **2-Benzoyl-3-phenylbenzo[*f*]chromen-1-one (6e)**

Yield: 68% (255 mg, 0.68 mmol).

Characteristic: Colourless solid.

Melting point: 168-169 ^◦^C

^1^H NMR (300 MHz, CDCl_3_): δ 7.23-7.54 (8H, m), 7.60-7.63 (1H, m), 7.73 (2H, dd, *J* = 8.1, 1.2 Hz), 7.79-7.90 (4H, m), 7.96 (1H, s).

^13^C NMR (75 MHz, CDCl_3_): δ 118.1, 120.7, 124.5, 125.9, 127.8, 128.1, 128.6, 128.7, 128.9, 129.4, 129.8, 130.2, 130.3, 131.2, 133.1, 136.9, 142.7, 151.5, 162.3, 176.6, 195.5.

FTIR (KBr, cm^-1^): 1227, 1261, 1345, 1391, 1447, 1595, 1629.

HR-MS (*m*/*z*) for C_26_H_17_O_3_ (M^+^+H): Calculated 377.1178, found 377.1174.

5.6. **3-Benzoyl-2-thiophen-3-yl-chromen-4-one (6f)**

Yield: 82% (272 mg, 0.82 mmol).

Characteristic: Yellowish solid.

Melting point: 117-119 ^◦^C

^1^H NMR (300 MHz, CDCl_3_): δ 7.01-7.08 (1H, m), 7.23-7.41 (7H, m), 7.46 (1H, d, *J* = 1.2 Hz), 7.68 (2H, dd, *J* = 7.8, 1.2 Hz), 8.01 (1H, dd, *J* = 3.0, 1.2 Hz).

^13^C NMR (75 MHz, CDCl_3_): δ 117.9, 118.2, 123.3, 125.9, 126.0, 128.0, 128.1, 128.5, 128.7, 128.8, 129.8, 134.0, 134.8, 136.7, 156.3, 162.6, 174.1, 195.5.

FT-IR (KBr, cm^-1^): 1291, 1328, 1446, 1488, 1534, 1577, 1628, 1710, 2850.

HR-MS (*m*/*z*) for C_20_H_12_O_3_S (M^+^): Calculated 332.0507, found 332.0509.

5.7. **3-Benzoyl-8-methyl-2-(p-tolyl)-chromen-4-one (6g)**

Yield: 76% (269 mg, 0.76 mmol).

Characteristic: Colourless solid.

Melting point: 160-162 ^◦^C

^1^H NMR (300 MHz, CDCl_3_): δ 2.25 (3H, s), 2.40 (3H, s), 6.89 (1H, t, *J* = 7.5 Hz), 7.02 (1H, d, *J* = 6.9 Hz), 7.11-7.14 (2H, m), 7.20-7.34 (3H, m), 7.44 (1H, t, *J* = 7.5 Hz), 7.53-7.57 (1H, m), 7.64-7.68 (2H, m), 7.75 (1H, d, *J* = 6.9 Hz).

^13^C NMR (75 MHz, CDCl_3_): δ 15.5, 21.6, 121.4, 125.8, 125.9, 126.3, 128.0, 128.4, 128.5, 128.7, 129.2, 129.8, 130.0, 134.0, 134.2, 134.3, 135.5, 136.8, 138.2, 157.2, 162.6, 176.1, 195.4.

FTIR (KBr, cm^-1^): 1179, 1235, 1291, 1364, 1441, 1464, 1578, 1600, 1630, 1708.

HR-MS (*m*/*z*) for C_24_H_19_O_3_ (M^+^+H): Calculated 355.1334, found 355.1339.

5.8. **3-Benzoyl-8-methoxy-2-(p-tolyl)-chromen-4-one (6h)**

Yield: 80% (296 mg, 0.80 mmol).

Characteristic: Yellow solid.

Melting point: 145-146 ^◦^C

^1^H NMR (300 MHz, CDCl_3_): δ 2.43 (3H, s), 3.83 (3H, s), 6.83-6.87 (1H, m), 6.98 (1H, d, *J* = 6.6 Hz), 7.14-7.33 (6H, m), 7.48 (1H, dd, *J* = 3.6 Hz), 7.72 (2H, d, *J* = 8.1 Hz), 7.82-7.92 (1H, m).

^13^C NMR (75 MHz, CDCl_3_): δ 21.6, 56.3, 112.2, 121.8, 126.0, 126.3, 128.0, 128.3, 128.5, 128.7, 129.2, 129.8, 130.0, 132.3, 133.0, 134.0, 136.7, 152.1, 156.8, 161.1, 175.6, 190.4.

FTIR (KBr, cm^-1^): 1094, 1223, 1384, 1400, 1571, 1638.

HR-MS (*m*/*z*) for C_24_H_18_O_4_ (M^+^): Calculated 370.1205, found 370.1207.

5.9. **4-Oxo-4*H*-chromene-2,3-dicarboxylic acid diethyl ester (6i)**

Yield: 65% (188 mg, 0.65 mmol).

Characteristic: Yellow oil.

^1^H NMR (500 MHz, CDCl_3_): δ 1.24-1.33 (6H, m), 4.24-4.40 (4H, m), 6.98 (1H, d, *J* = 8.0 Hz), 7.03 (1H, t, *J* = 7.5 Hz), 7.30-7.36 (2H, m).

^13^C NMR (100 MHz, CDCl_3_): δ 14.0, 14.2, 61.2, 63.6, 116.9, 118.2, 121.6, 122.5, 129.2, 132.7, 151.5, 157.2, 164.1, 169.3, 176.1.

FTIR (neat, cm^-1^): 1275, 1356, 1446, 1667, 1714, 1760.

HR-MS (*m*/*z*) for C_15_H_14_O_6_ (M^+^): Calculated 290.0790, found 290.0794.

5.10. **6-Bromo-4-oxo-4*H*-chromene-2,3-dicarboxylic acid diethyl ester (6j)**

Yield: 87% (321 mg, 0.87 mmol).

Characteristic: Yellow oil.

^1^H NMR (300 MHz, CDCl_3_): δ 1.27-1.36 (6H, m), 4.25-4.43 (4H, m), 6.90 (1H, d, *J* = 7.5 Hz), 7.46 (1H, dd, *J* = 7.5, 2.1 Hz), 7.73 (1H, s).

^13^C NMR (75 MHz, CDCl_3_): δ 13.8, 14.1, 61.3, 63.6, 114.5, 118.6, 119.9, 122.7, 131.1, 133.6, 150.3, 155.6, 163.5, 168.8, 175.3.

FTIR (neat, cm^-1^): 1206, 1262, 1280, 1470, 1642, 1713, 1751.

HR-MS (*m*/*z*) for C_15_H_13_BrO_6_ (M^+^): Calculated 367.9896, found 367.9893 (One of the major peaks).

5.11. **3-Benzoyl-2-phenylchromen-4-one** **(6k)**

Yield: 70% (228 mg, 0.70 mmol).

Characteristic: Colourless solid.

Melting point: 120-121^◦^C

^1^H NMR (300 MHz, CDCl_3_): δ 7.30-7.60 (7H, m), 7.63-7.79 (4H, m), 7.92 (2H, dd, *J* = 7.2, 1.5 Hz), 8.25 (1H, dd, *J* = 7.8, 1.5 Hz).

^13^C NMR (75 MHz, CDCl_3_): δ 118.1, 122.7, 123.3, 125.6, 126.1, 128.5, 128.6, 129.4, 131.4, 131.8, 133.6, 134.3, 137.1, 156.1, 162.4, 176.4, 193.4.

FTIR (KBr, cm^-1^): 1093, 1127, 1268, 1384, 1638, 1687, 1707.

HR-MS (*m*/*z*) for C_22_H_14_O_3_ (M^+^): Calculated 326.0943, found 326.0941.

**6. Characterization data of aza-flavones (7a-c)**

6.1. **3-Benzoyl-6,8-dibromo-2-phenyl-1*H*-quinolin-4-one** **(7a)**

Yield: 76% (367 mg, 0.76 mmol).

Characteristic: Yellow solid.

Melting point: 170-171^◦^C.

^1^H NMR (400 MHz, CDCl_3_): δ 7.27-7.40 (5H, m), 7.50-7.54 (1H, m), 7.66-7.75 (4H, m), 8.01 (1H, d, *J* = 2.0 Hz), 8.21 (1H, s), 8.24 (1H, d, *J* = 2.0 Hz).

^13^C NMR (100 MHz, CDCl_3_): δ 120.5, 126.6, 127.7, 128.6, 129.6, 129.8, 130.0, 133.8, 134.4, 136.6, 137.1, 137.2, 137.4, 138.8, 139.9, 144.1, 158.0, 173.5, 196.3.

FT-IR (KBr, cm^-1^): 1314, 1400, 1448, 1637, 2067.

HR-MS (*m*/*z*) for C_22_H_13_Br_2_NO_2_ (M^+^): Calculated 480.9313, found 480.9317 (One of the major peaks).

6.2. **3-Benzoyl-6,8-dibromo-2-(p-tolyl)-1*H*-quinolin-4-one (7b)**

Yield: 80% (397 mg, 0.80 mmol).

Characteristic: Colourless solid.

Melting point: 196-199 ^◦^C

^1^H NMR (400 MHz, CDCl_3_): δ 2.37 (3H, s), 6.83 (1H, s), 7.04-7.12 (2H, m), 7.23-7.33 (4H, m), 7.46-7.50 (1H, m), 7.63 (2H, d, *J* = 6.8 Hz), 7.89 (1H, d, *J* = 2.4 Hz), 7.90 (1H, d, *J* = 2.0 Hz).

^13^C NMR (100 MHz, CDCl_3_): δ 21.6, 116.5, 121.8, 125.5, 127.2, 127.3, 128.7, 129.4, 129.5, 129.7, 132.4, 133.0, 135.7, 136.6, 143.4, 144.2, 159.3, 171.8, 190.3.

FT-IR (KBr, cm^-1^): 1298, 1412, 1450, 1640, 1701, 2068.

HR-MS (*m*/*z*) for C_23_H_15_Br_2_NO_2_ (M^+^): Calculated 494.9470, found 494.9472 (One of the major peaks).

6.3. **6,8-Dibromo-4-oxo-1,4-dihydro-quinoline-2,3-dicarboxylic acid diethyl ester (7c)**

Yield: 85% (380 mg, 0.85 mmol).

Characteristic: Colourless solid.

Melting point: 120-123 ^◦^C

^1^H NMR (300 MHz, CDCl_3_): δ 1.43 (6H, dt, *J* = 7.2 Hz), 4.44 (2H, q, *J* = 7.2 Hz), 4.52 (2H, q, *J* = 7.2 Hz), 8.05 (1H, d, *J* = 2.0 Hz), 8.26 (1H, d, *J* = 2.0 Hz), 8.66 (1H, s).

^13^C NMR (75 MHz, CDCl_3_): δ 14.1, 14.2, 62.4, 62.5, 122.2, 124.3, 126.6, 128.9, 138.9, 139.0, 144.2, 151.1, 164.4, 166.1, 176.3.

FT-IR (KBr, cm^-1^): 1062, 1159, 1264, 1287, 1459. 1584, 1716, 1741.

HR-MS (*m*/*z*) for C_15_H_13_Br_2_NO_5_ (M^+^): Calculated 444.9160, found 444.9164 (One of the major peaks).

**7. Characterization data of thio-flavones (8a,b)**

7.1. **4-Oxo-4*H*-thiochromene-2,3-dicarboxylic acid diethyl ester (8a)**

Yield: 70% (214 mg, 0.70 mmol).

Characteristic: Yellow solid.

Melting point: 123-125 ^◦^C

^1^H NMR (300 MHz, CDCl_3_): δ 1.15-1.27 (6H, m), 4.15-4.34 (4H, m), 6.90-6.99 (2H, m), 7.23-7.31 (2H, m).

^13^C NMR (100 MHz, CDCl_3_): δ 13.8, 14.1, 61.1, 63.4, 116.8, 118.1, 121.5, 122.4, 129.0, 132.5, 135.1, 151.3, 163.9, 169.2, 186.8.

FTIR (KBr, cm^-1^): 1296, 1366, 1458, 1571, 1607, 1641, 1704, 1736.

HR-MS (*m*/*z*) for C_15_H_14_O_5_S (M^+^): Calculated 306.0562, found 306.0567.

7.2. **3-Benzoyl-2-phenylthiochromen-4-one (8b)**

Yield: 56% (191 mg, 0.56 mmol).

Characteristic: Colourless solid.

Melting point: 156-159 ^◦^C

^1^H NMR (300 MHz, CDCl_3_): 7.19-7.48 (8H, m), 7.55-7.64 (4H, m), 8.15-8.17 (4H, m).

^13^C NMR (75 MHz, CDCl_3_): δ 120.1, 128.6, 129.5, 130.7, 133.0, 134.0, 136.9, 152.3, 178.0, 187.4.

FTIR (KBr, cm^-1^): 1271, 1345, 1578, 1612, 1650, 1706, 1710, 1732.

HR-MS (*m*/*z*) for C_22_H_14_O_2_S (M^+^): Calculated 342.0715, found 342.0712.

**8. General procedure of oxidative coupling toward 3-oxyenals (9)**

To a solution of salisaldehyde (1.0 mmol) and another aldehyde (1.0 mmol) in THF (20 mL) triethylamine (1.1 mmol, 111 mg) was added drop wise under stirring conditions. The propargyl alcohol (1.0 mmol) was added to it. The Mn^VI^-NPs catalyst (10 mol %, 26 mg) and sodium periodate (1.1 mmol, 235 mg) were added and allowed to reflux at 70 ^o^C for 4 h. The reaction was monitored by thin layer chromatography (TLC). The solvent was removed from the post reaction mixture in a rotary evaporator and the residue was extracted with EtOAc (2x20 mL). The combined organic layer was washed successively with saturated sodium bicarbonate solution (1x10 mL) and brine (2x10 mL). It was dried over anhydrous Na_2_SO_4_, filtered and evaporated in a rotary evaporator under reduced pressure at room temperature. Thus, the three component cyclisation reaction of salicylaldehyde (2.0 mmol, 244 mg) with 3-phenyl-2-propyn-1-ol (1.0 mmol, 132 mg) afforded 3-oxyenal (**9a**) which was isolated after purification by column chromatography on silica gel (60-120 mesh) using ethyl acetate-petroleum ether (1:9, v/v) as an eluent in an yield of 70% (261 mg, 0.70 mmol). The synthesized compounds (**9a-c**) were characterized by means of NMR (^1^H and ^13^C), FTIR, Mass (HR-MS) spectral analysis.

**9. Characterization data of 3-oxyenals (9a-c)**

9.1. **2-[3-Formyl-4-hydroxy-4-(2-hydroxyphenyl)-2-phenyl-but-2-enyl]benzaldehyde** **(9a)**

Yield: 70% (261 mg, 0.70 mmol).

Characteristic: Yellow oil.

^1^H NMR (300 MHz, CDCl_3_): δ 1.68 (1H, s), 5.58 (1H, s), 6.98 (1H, t, *J* = 7.5 Hz), 7.07-7.12 (1H, m), 7.16-7.26 (2H, m), 7.32-7.45 (3H, m), 7.52-7.60 (4H, m), 7.67-7.72 (2H, m), 9.57 (1H, s), 9.88 (1H, s), 11.45 (1H, s).

^13^C NMR (75 MHz, CDCl_3_): δ 76.0, 115.4, 116.5, 119.7, 120.9, 124.0, 125.2, 127.9, 128.0, 128.4, 128.5, 129.4, 130.2, 131.2, 131.6, 132.5, 133.5, 136.5, 149.9, 158.9, 168.1, 190.5, 196.5.

FTIR (neat, cm^-1^): 1408, 1457, 1538, 1570, 1606, 1654, 1666.

HR-MS (*m*/*z*) for C_23_H_18_O_5_ (M^+^): Calculated 374.1154, found 374.1152.

9.2. **2-[3-Formyl-4-hydroxy-4-(4-nitrophenyl)-2-phenyl-but-2-enyl]benzaldehyde (9b)**

Yield: 76% (306 mg, 0.76 mmol).

Characteristic: Yellow oil.

^1^H NMR (400 MHz, CDCl_3_): δ 2.41 (1H, s), 5.60 (1H, s), 6.96 (1H, d, *J* = 6.8 Hz), 7.19 (1H, d, *J* = 2.0 Hz), 7.25-7.35 (4H, m), 7.45 (2H, t, *J* = 6.0 Hz), 7.57-7.63 (1H, m), 7.65 (2H, d, *J* = 6.0 Hz), 7.76 (2H, d, *J* = 6.0 Hz), 9.61 (1H, s), 10.14 (1H, s).

^13^C NMR (100 MHz, CDCl_3_): δ 75.0, 118.6, 120.0, 124.4, 125.6, 127.0, 128.0, 128.3, 128.6, 128.7, 129.0, 130.6, 131.5, 131.8, 132.6, 133.6, 134.7, 140.2, 151.3, 154.6, 162.8, 190.3, 192.9.

FTIR (neat, cm^-1^): 1409, 1449, 1528, 1575, 1609, 1650, 1667.

HR-MS (*m*/*z*) for C_23_H_17_NO_6_ (M^+^): Calculated 403.1056, found 403.1052.

9.3. **2-[3-(4-Bromophenyl)-2-formyl-3-hydroxy-1-phenylpropenyloxy]benzaldehyde (9c)**

Yield: 65% (284 mg, 0.65 mmol).

Characteristic: Yellow oil.

^1^H NMR (300 MHz, CDCl_3_): δ 2.23 (1H, s), 5.19 (1H, s), 6.98 (1H, d, *J* = 6.0 Hz), 7.21-7.49 (5H, m), 7.47-7.49 (2H, m), 7.58-7.63 (3H, m), 7.77 (2H, d, *J* = 8.4 Hz), 10.16 (1H, s), 10.38 (1H, s).

^13^C NMR (75 MHz, CDCl_3_): δ 75.1, 115.6, 116.4, 122.0, 124.1, 125.2, 127.5, 128.0, 128.3, 128.5, 128.6, 129.4, 130.2, 131.2, 131.4, 131.7, 132.5, 133.3, 136.7, 157.6, 167.3, 191.1, 195.7.

HR-MS (*m*/*z*) for C_23_H_17_BrO_4_ (M^+^): Calculated 436.0310, found 436.0313 (One of the major peaks).

**10. Reference**

1. M. Yoshida, K. Saito, Y. Fujino, Y. Fujii, T. Doi*; *Chem. Commun.,* 2012, **48**, 11796–11798.

**11. ESI-MS spectra of some representative compounds**

**SI Figure 9:** ESI-MS spectrum of compound **6a**

**SI Figure 10:** ESI-MS spectrum of compound **6b**


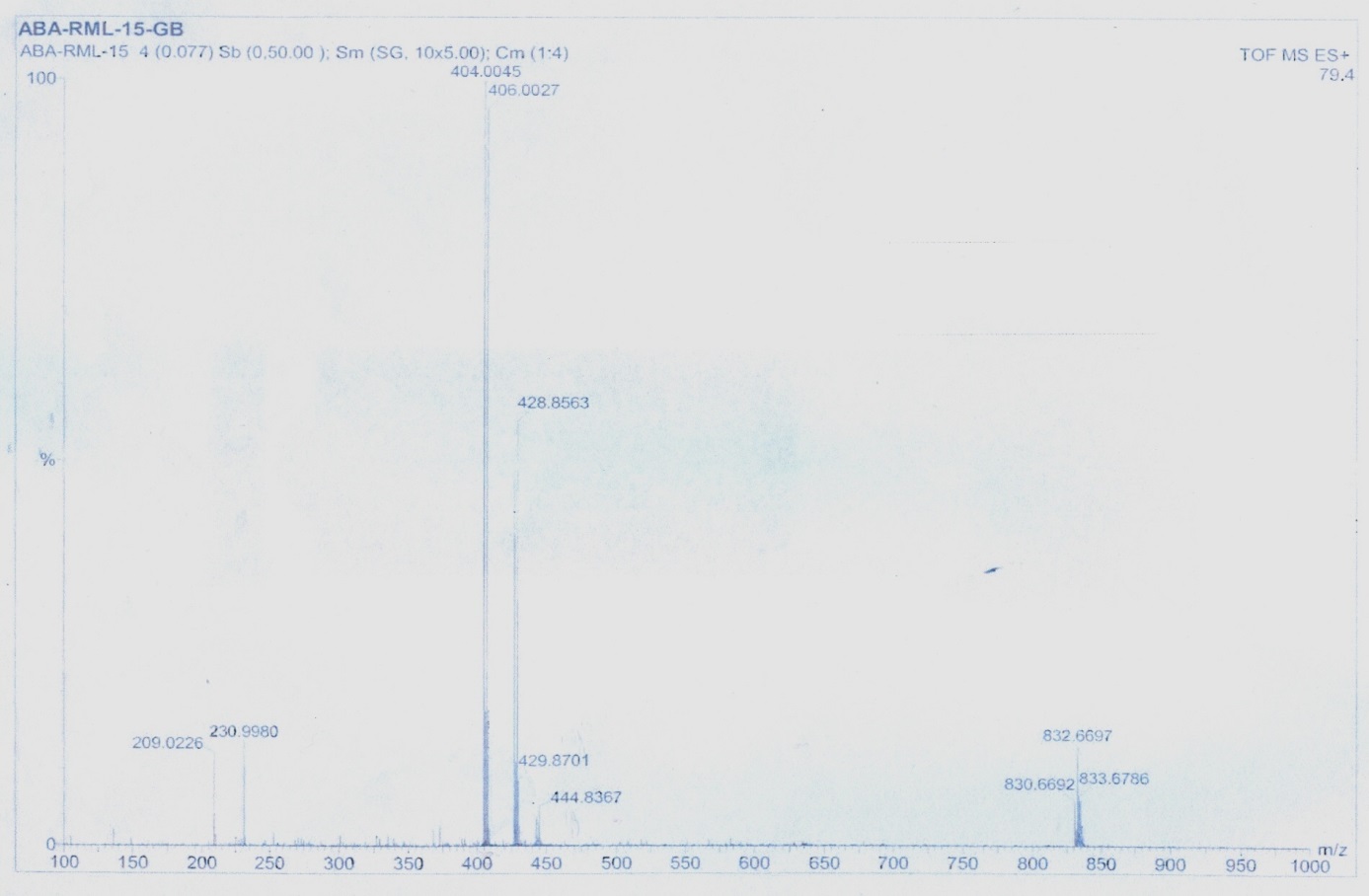


**SI Figure 11:** ESI-MS spectrum of compound **7a**

**
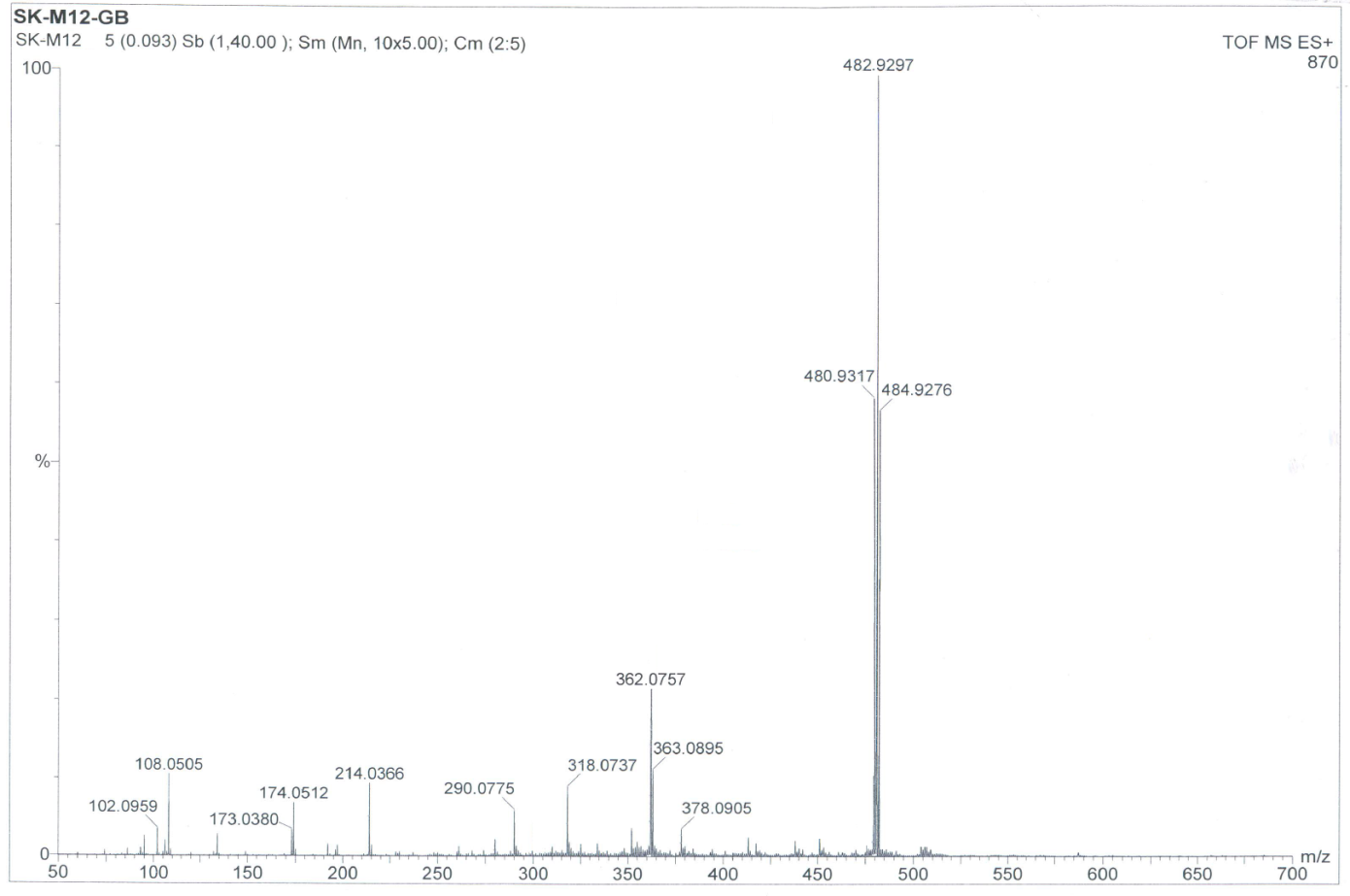
**

**SI Figure 12:** ESI-MS spectrum of compound **8a**

**
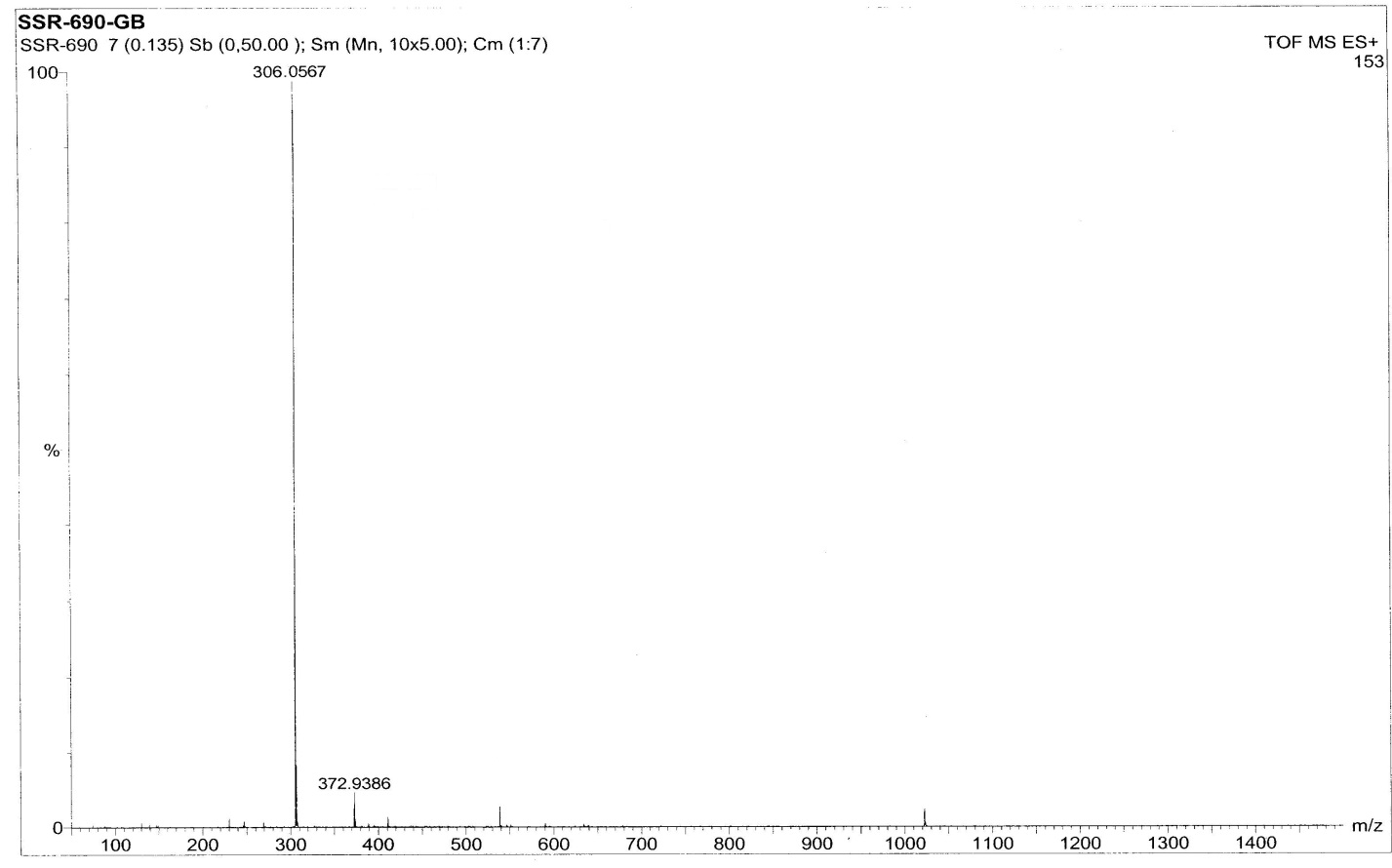
**

**SI Figure 13:** ESI-MS spectrum of compound **9a**

**
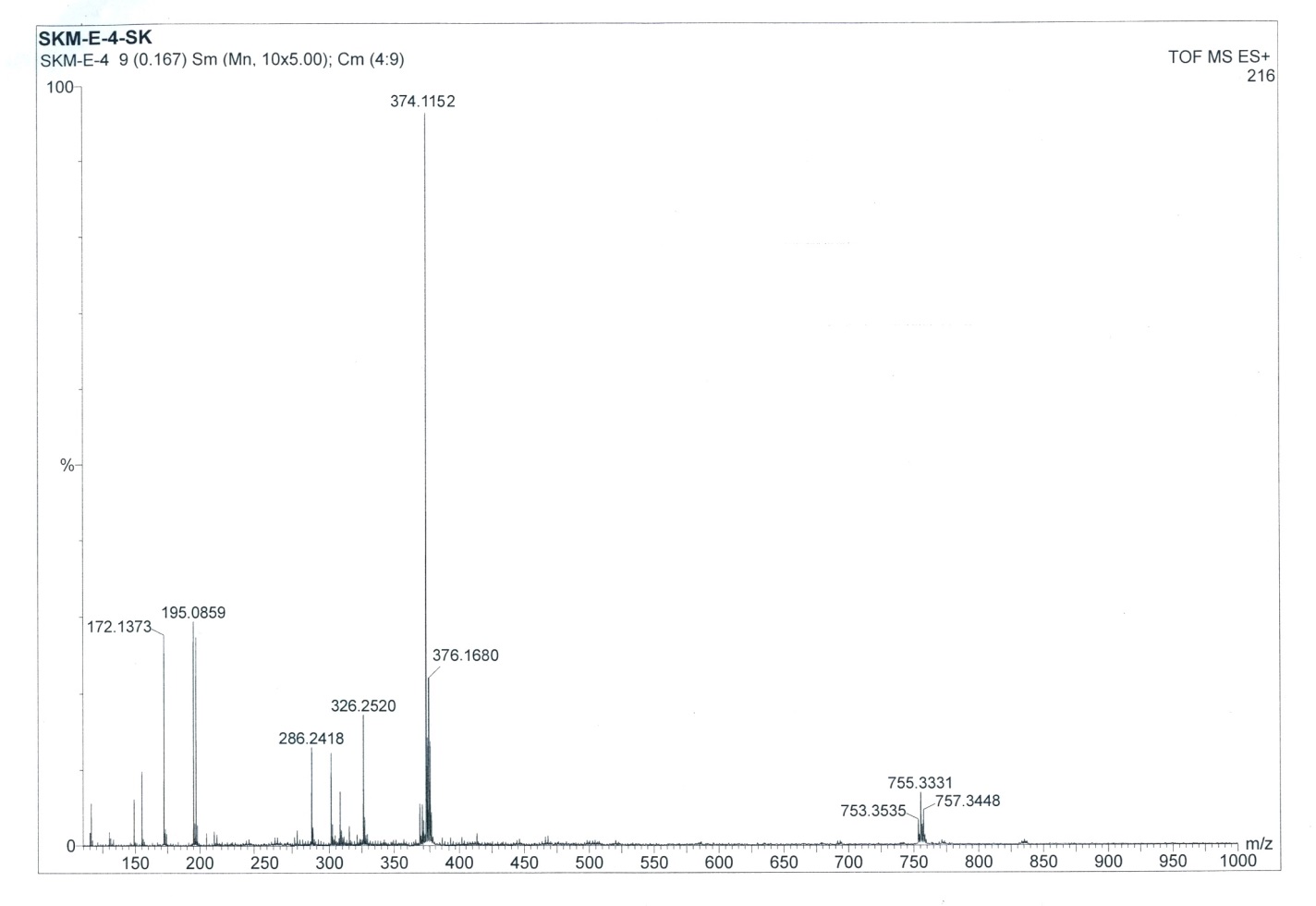
**

**12. ^1^H and ^13^C spectra of the compounds (6a-k, 7a-c, 8a-b, 9a-c)**

**SI Figure 14:** ^1^H and ^13^C-NMR spectra of compound **6a**


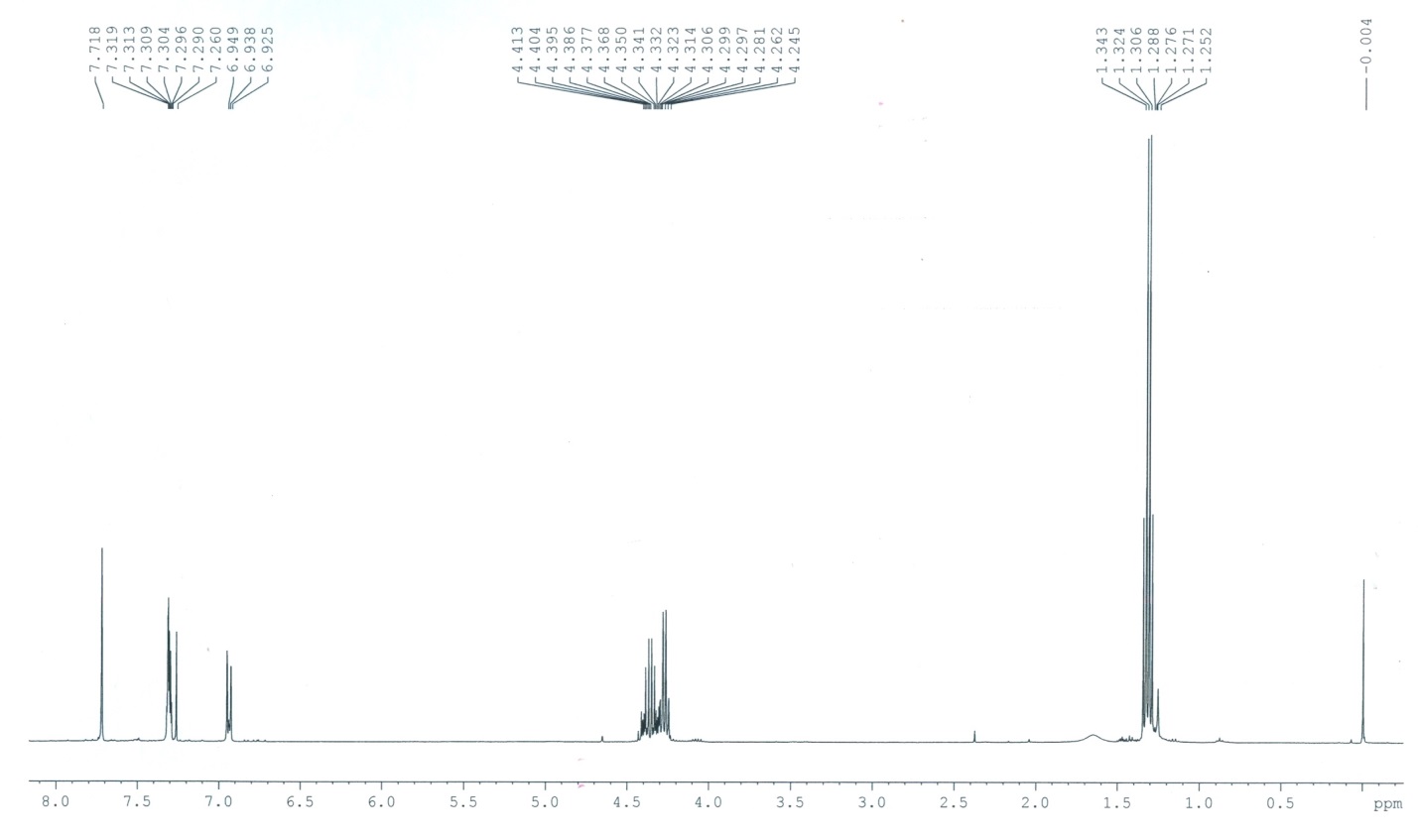

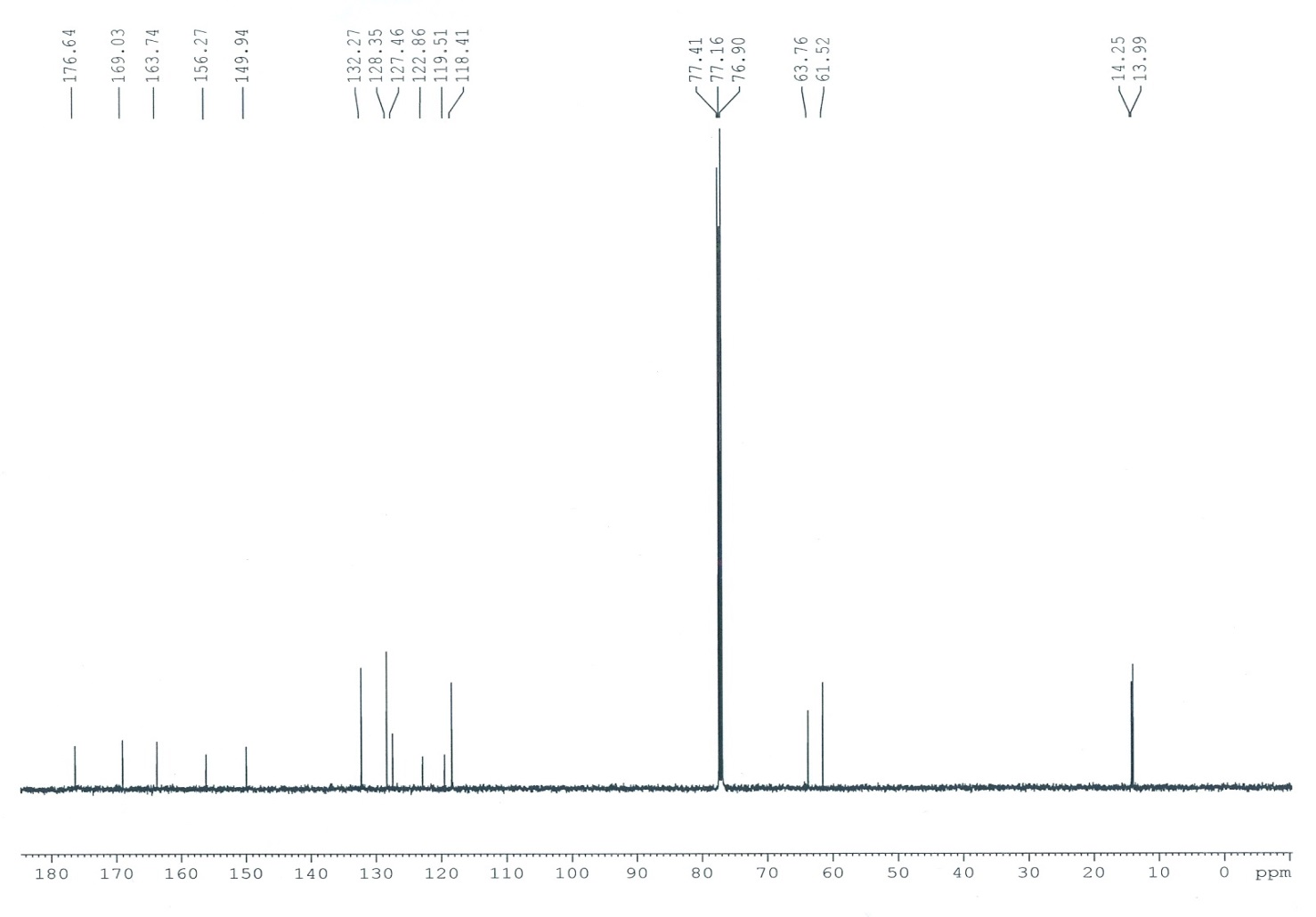


**SI Figure 15:** ^1^H and ^13^C-NMR spectra of compound **6b**


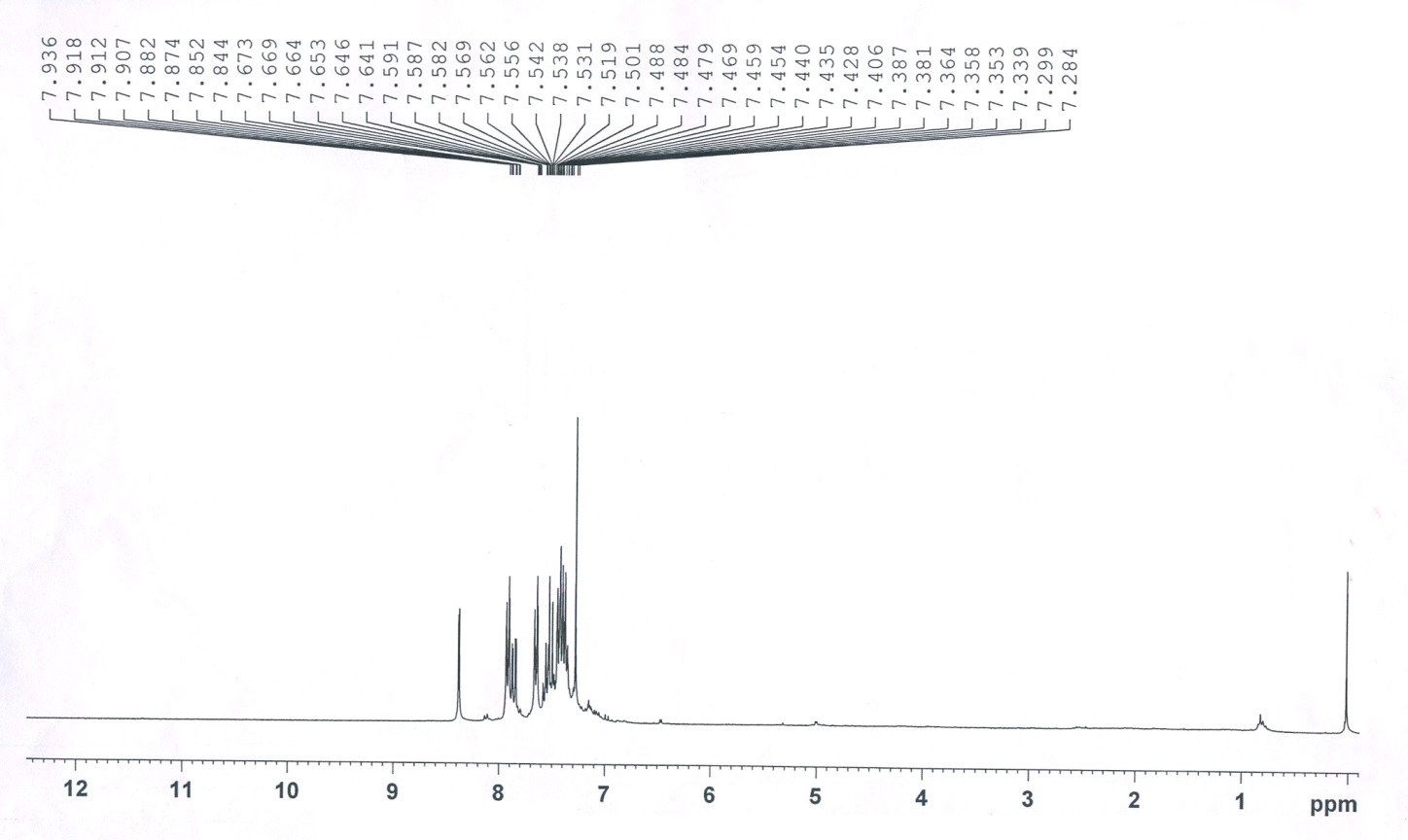

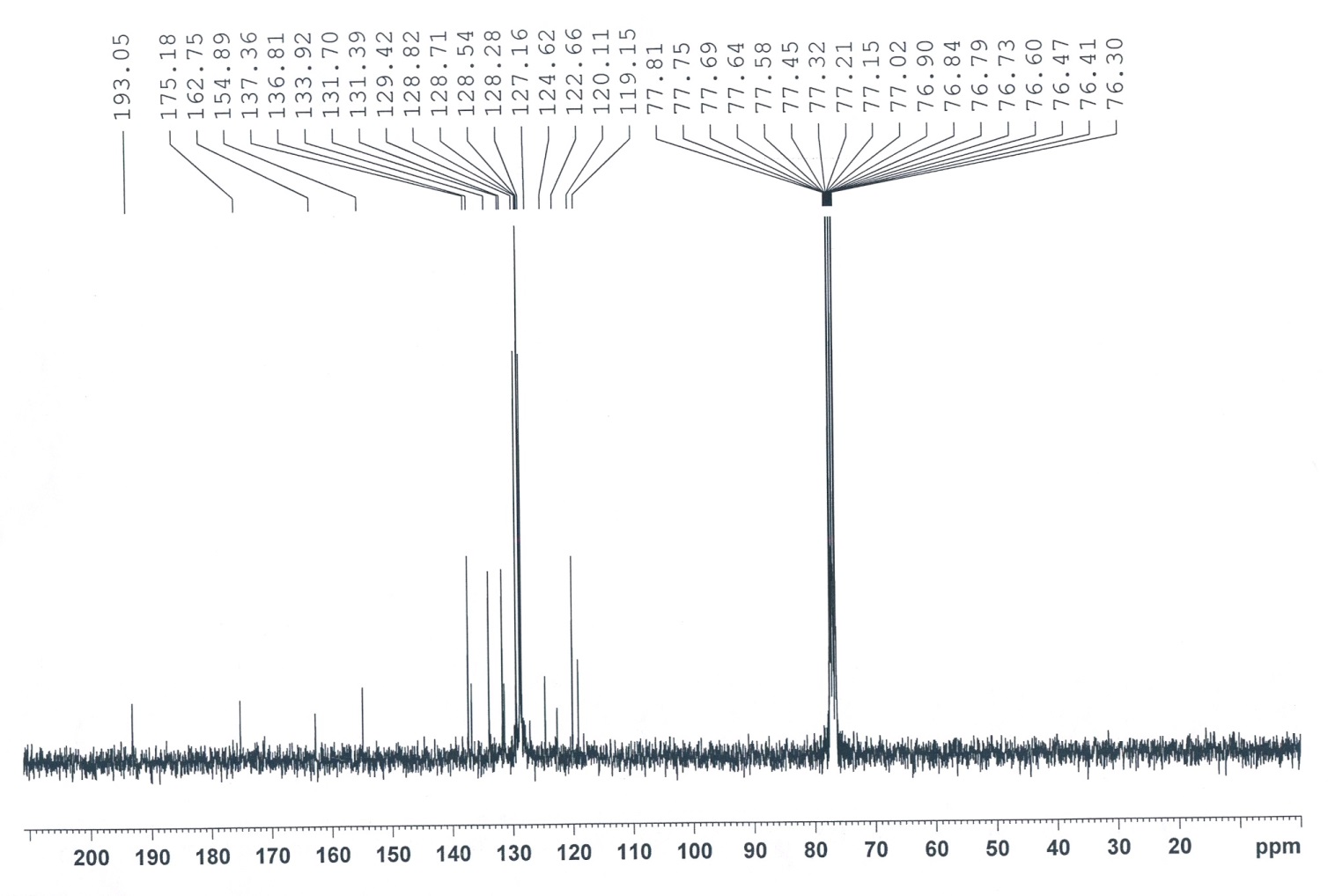


**SI Figure 16:** ^1^H and ^13^C-NMR spectra of compound **6c**


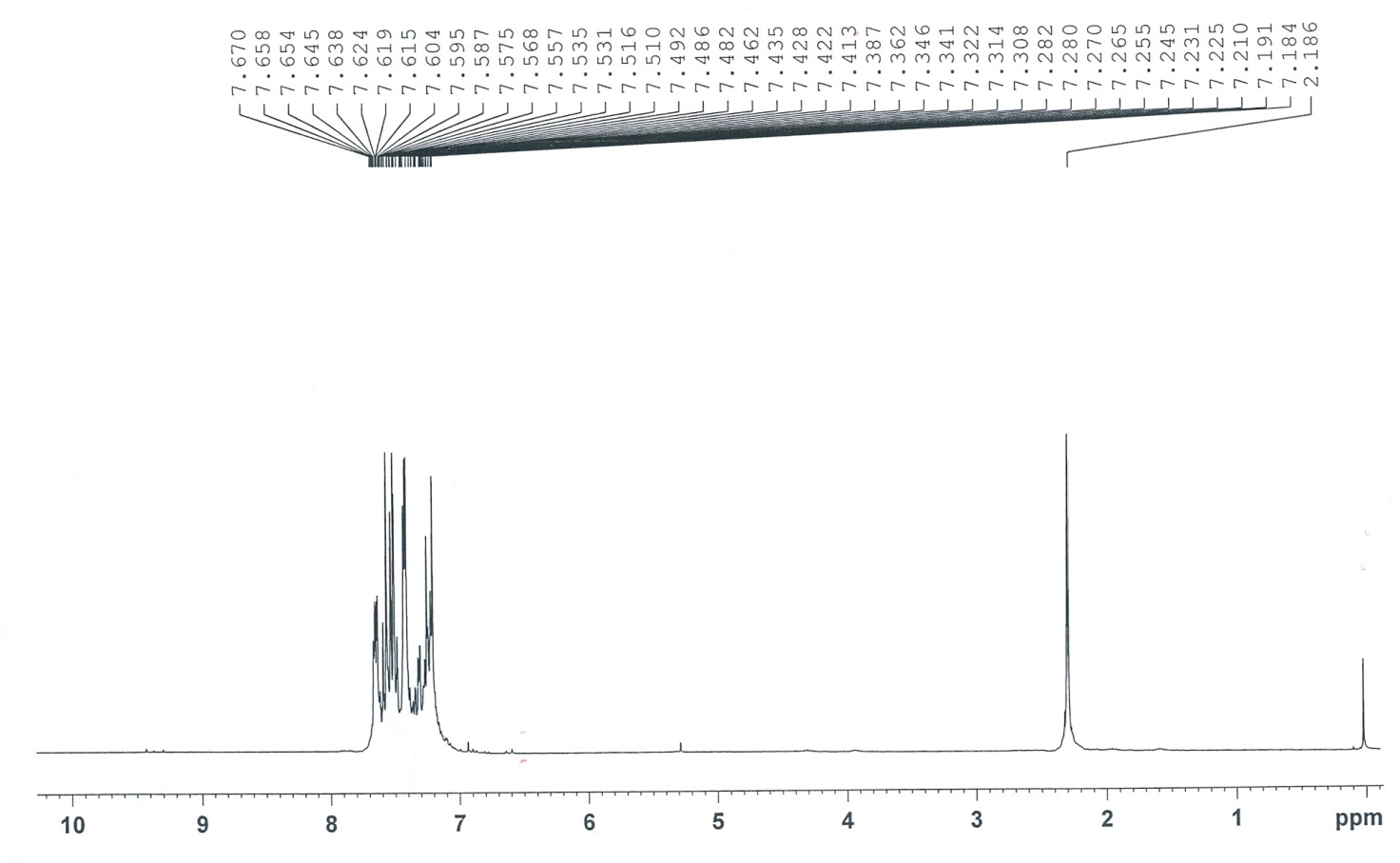

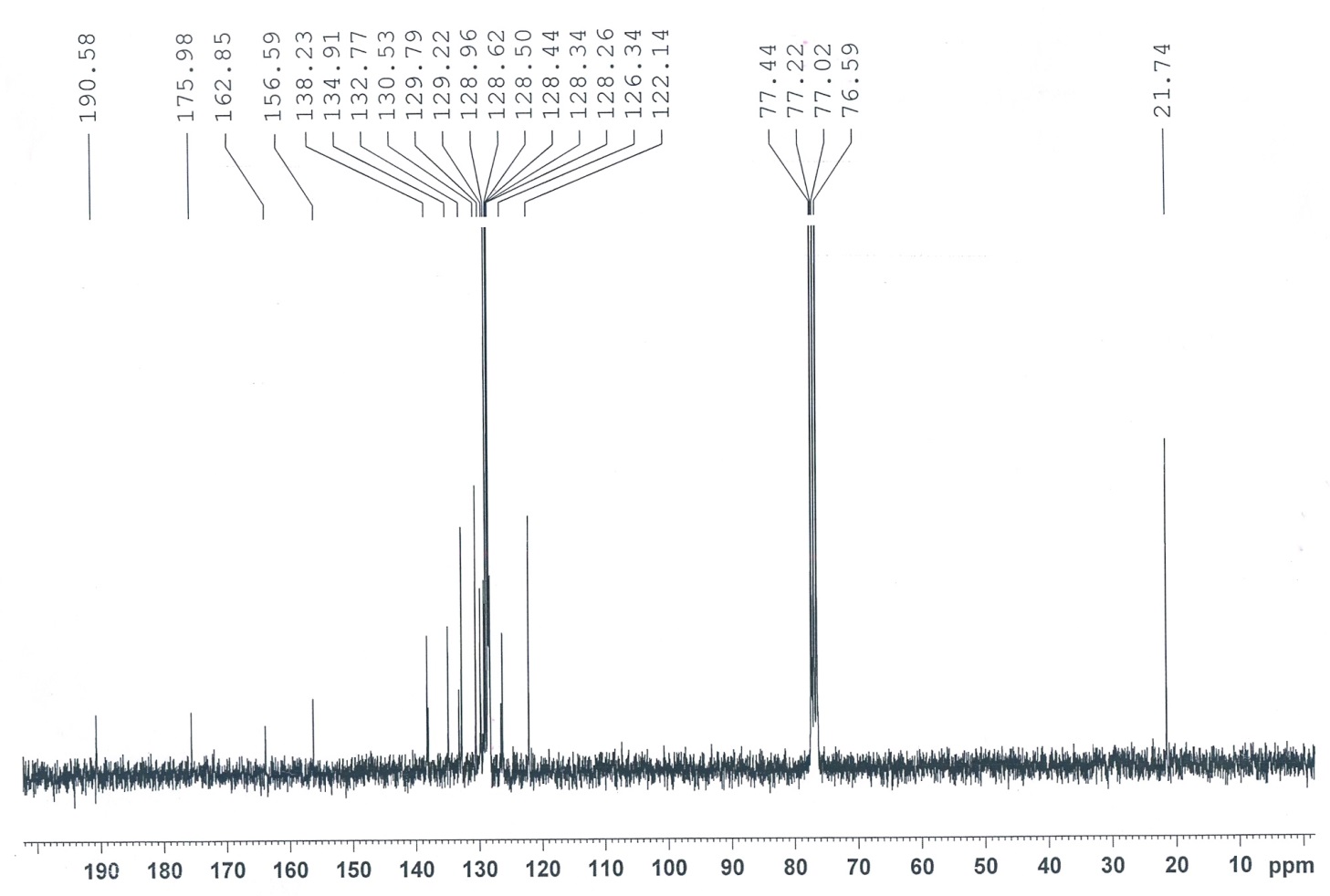


**SI Figure 17:** ^1^H and ^13^C-NMR spectra of compound **6d**


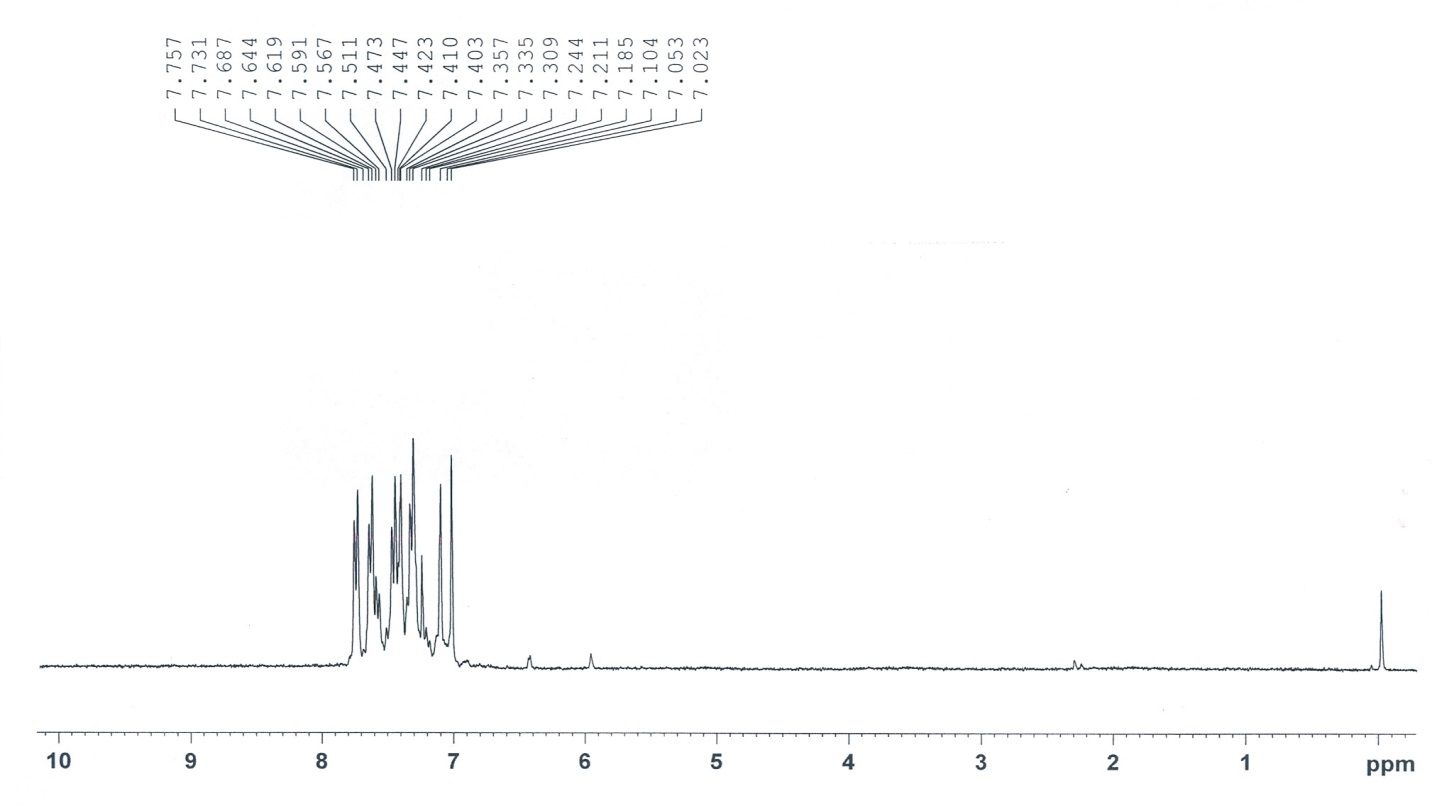

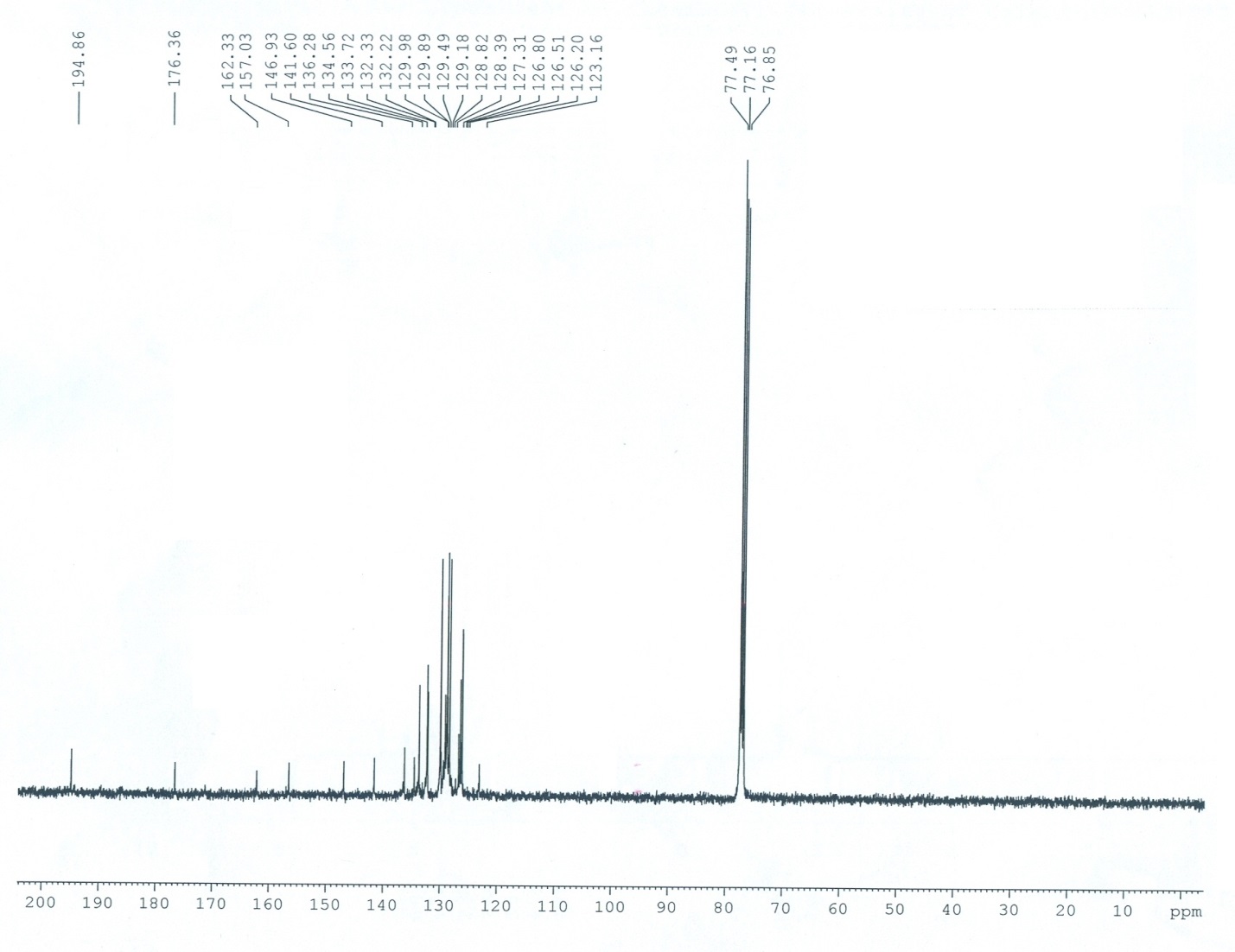


**SI Figure 18:** ^1^H and ^13^C-NMR spectra of compound **6e**


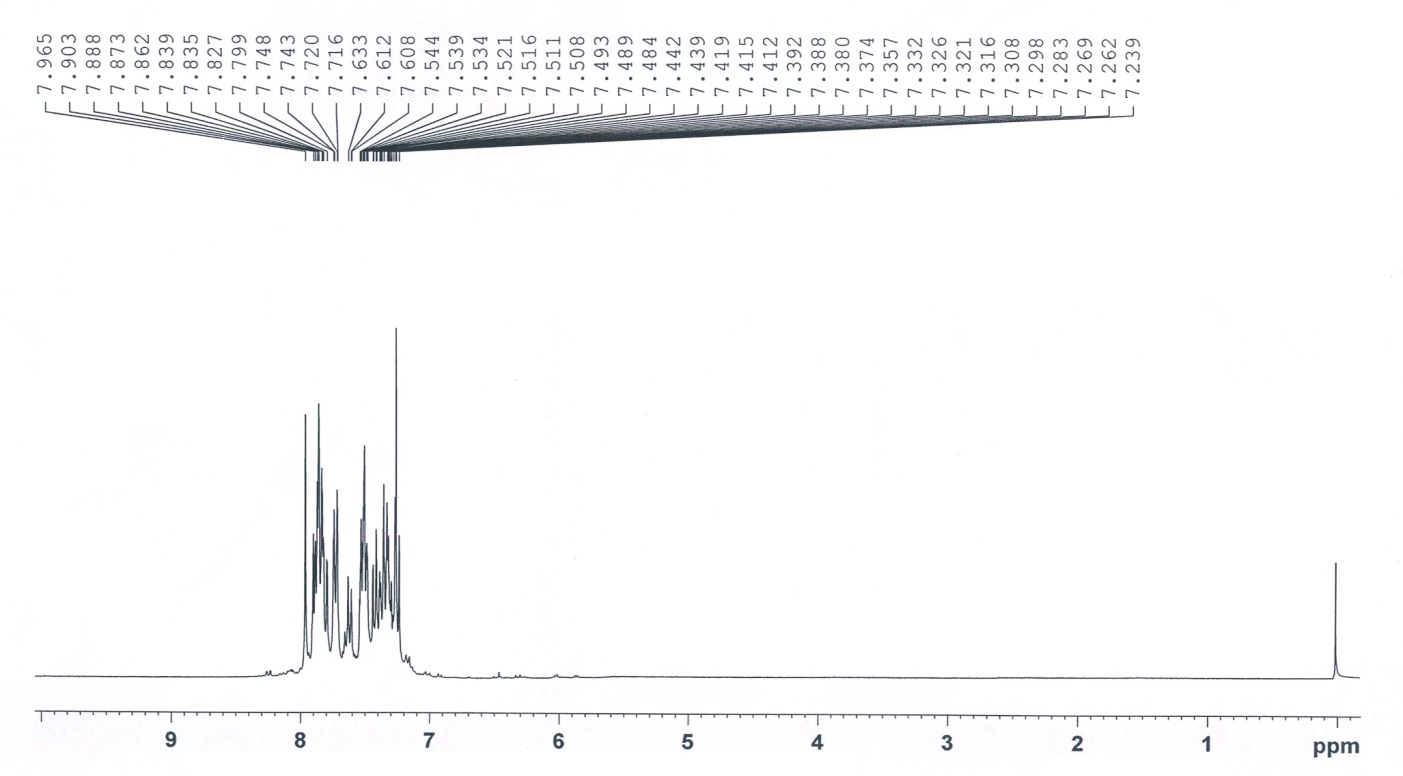

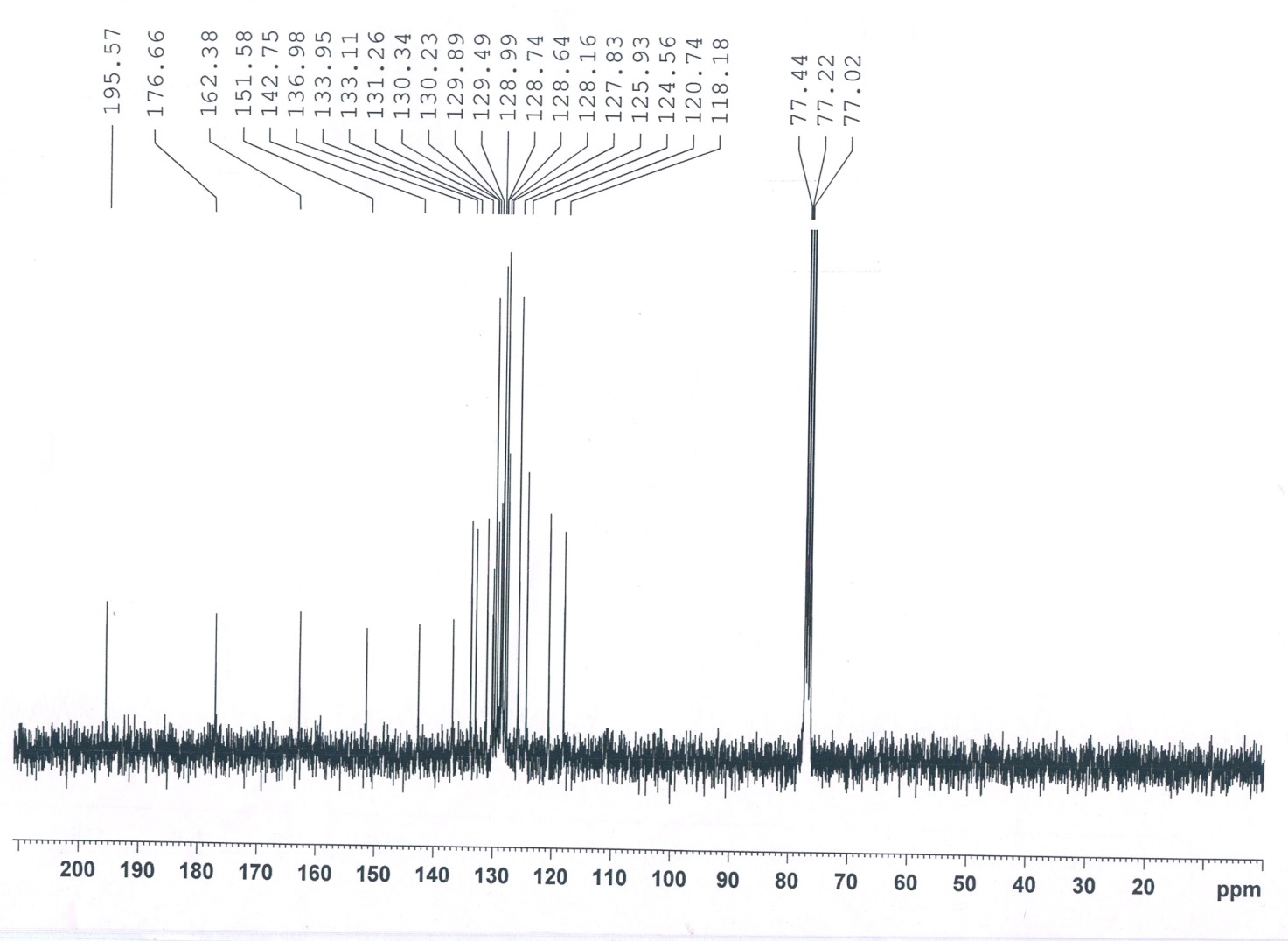


**SI Figure 19:** ^1^H and ^13^C-NMR spectra of compound **6f**


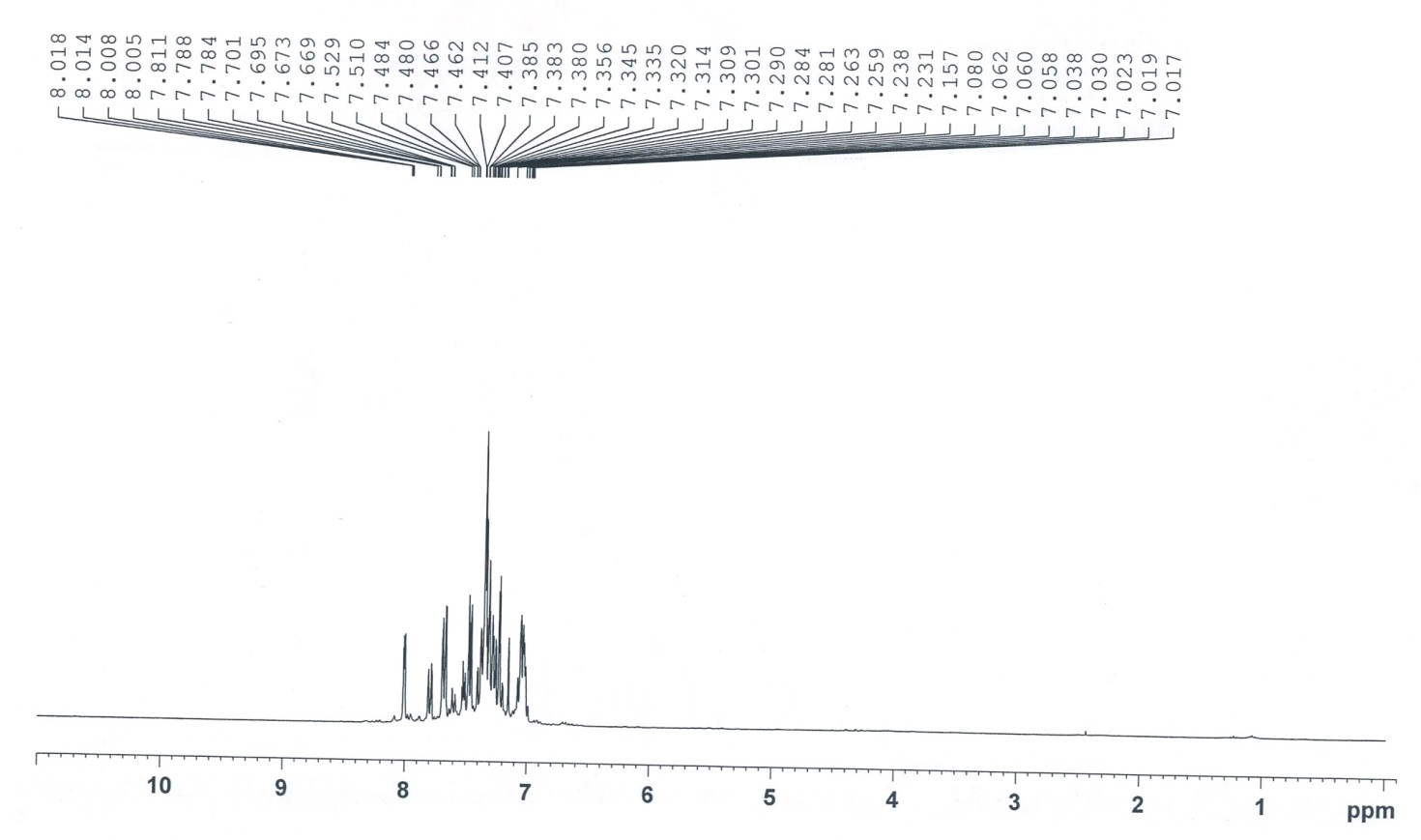

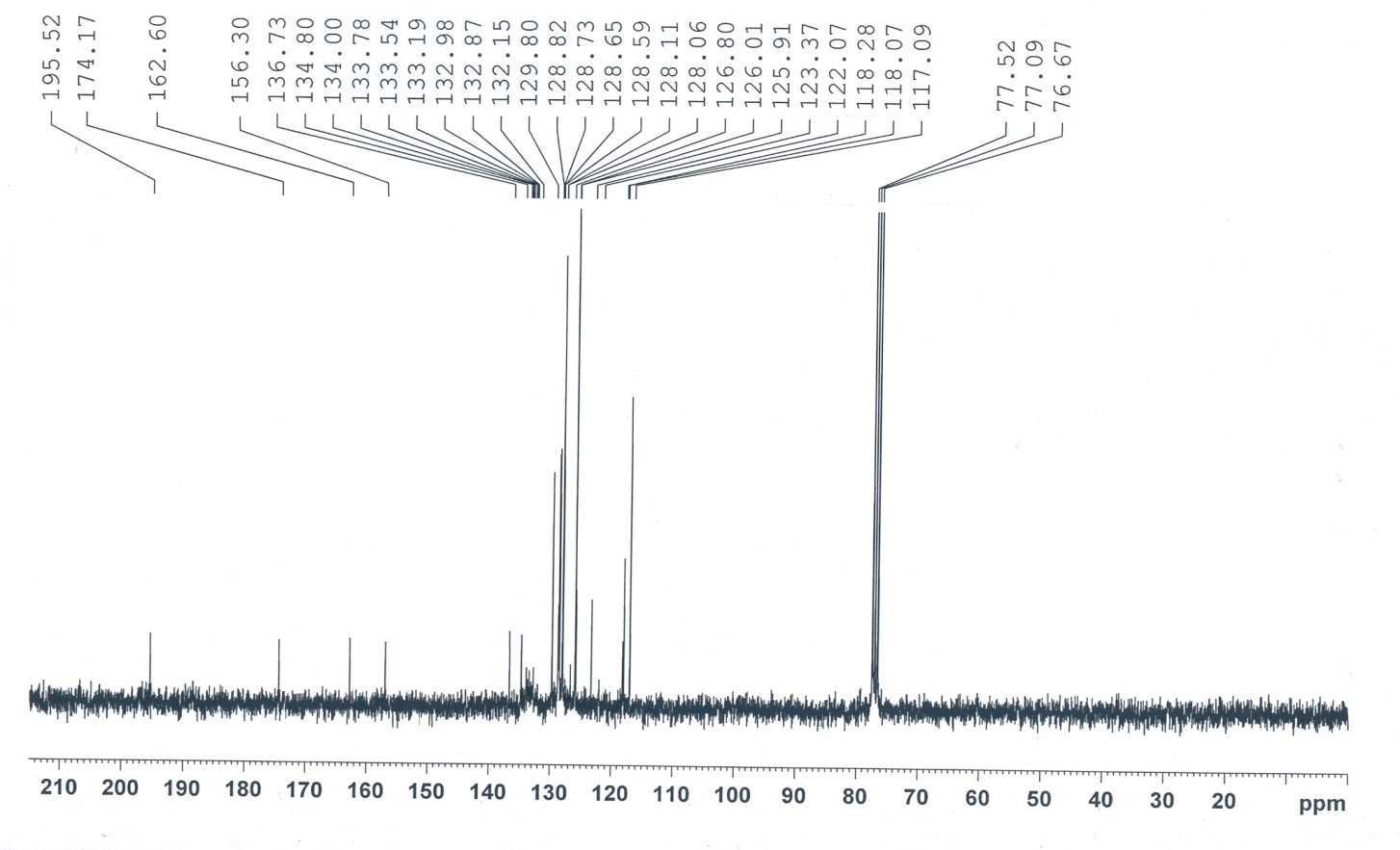


**SI Figure 20:** ^1^H and ^13^C-NMR spectra of compound **6g**


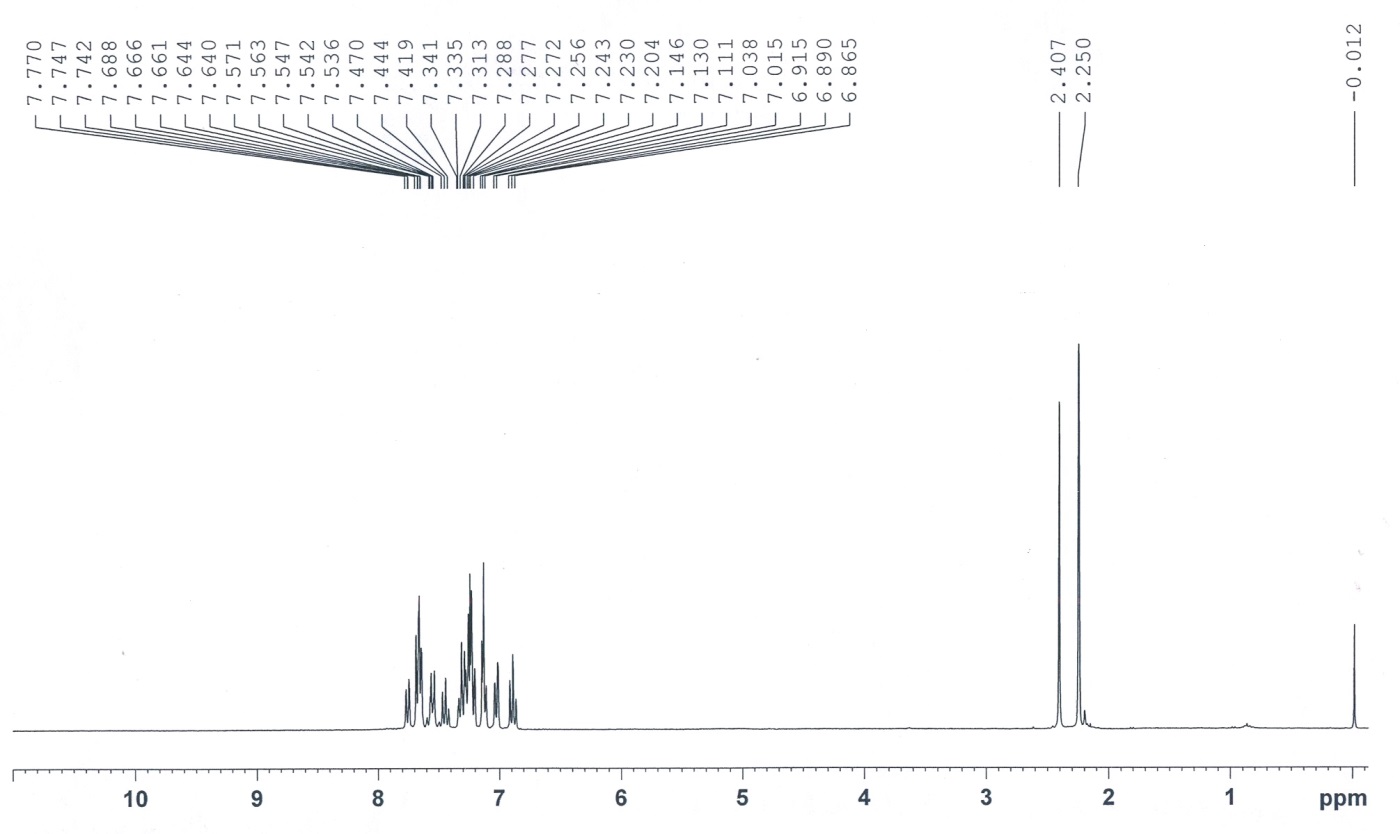

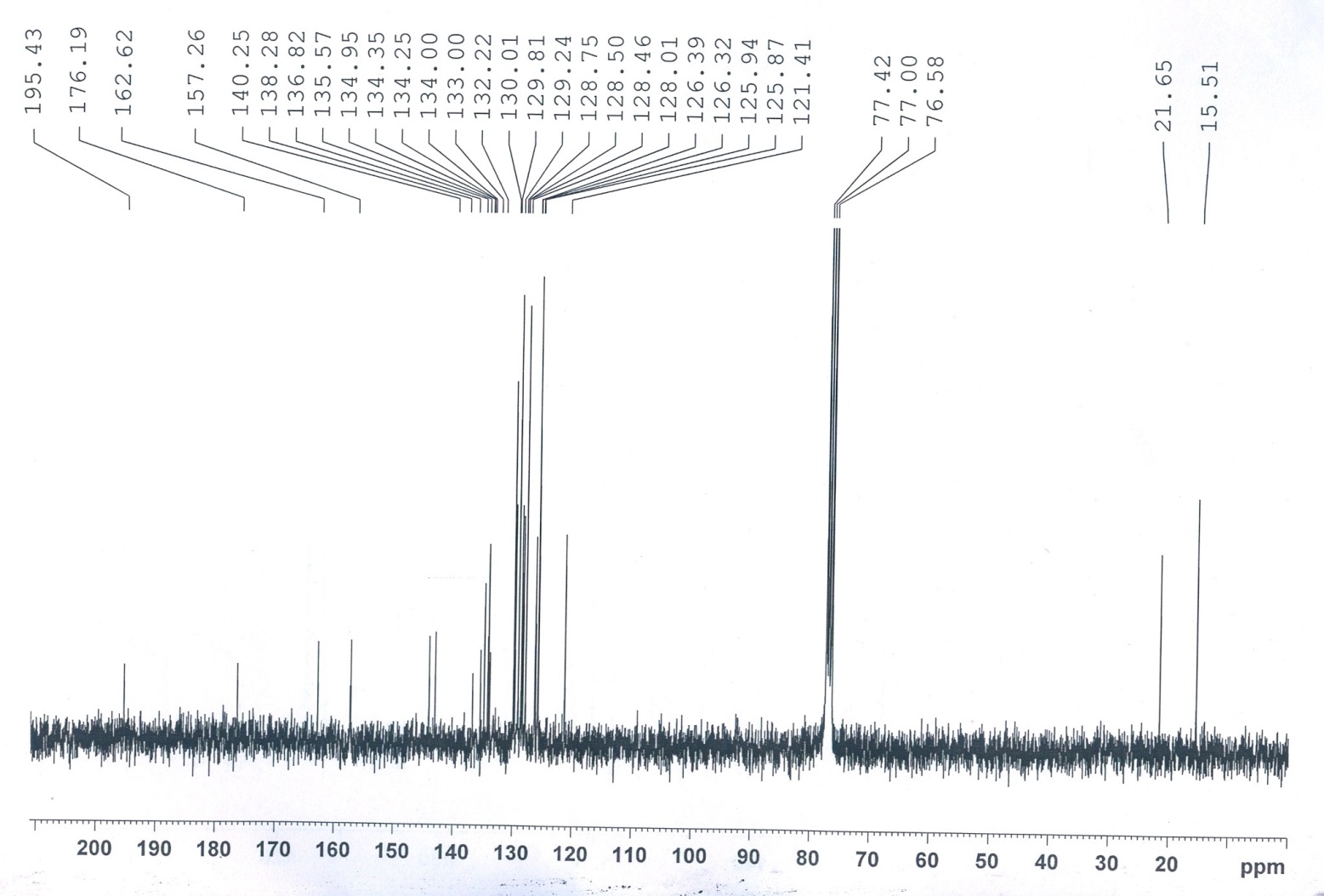


**SI Figure 21:** ^1^H and ^13^C-NMR spectra of compound **6h**


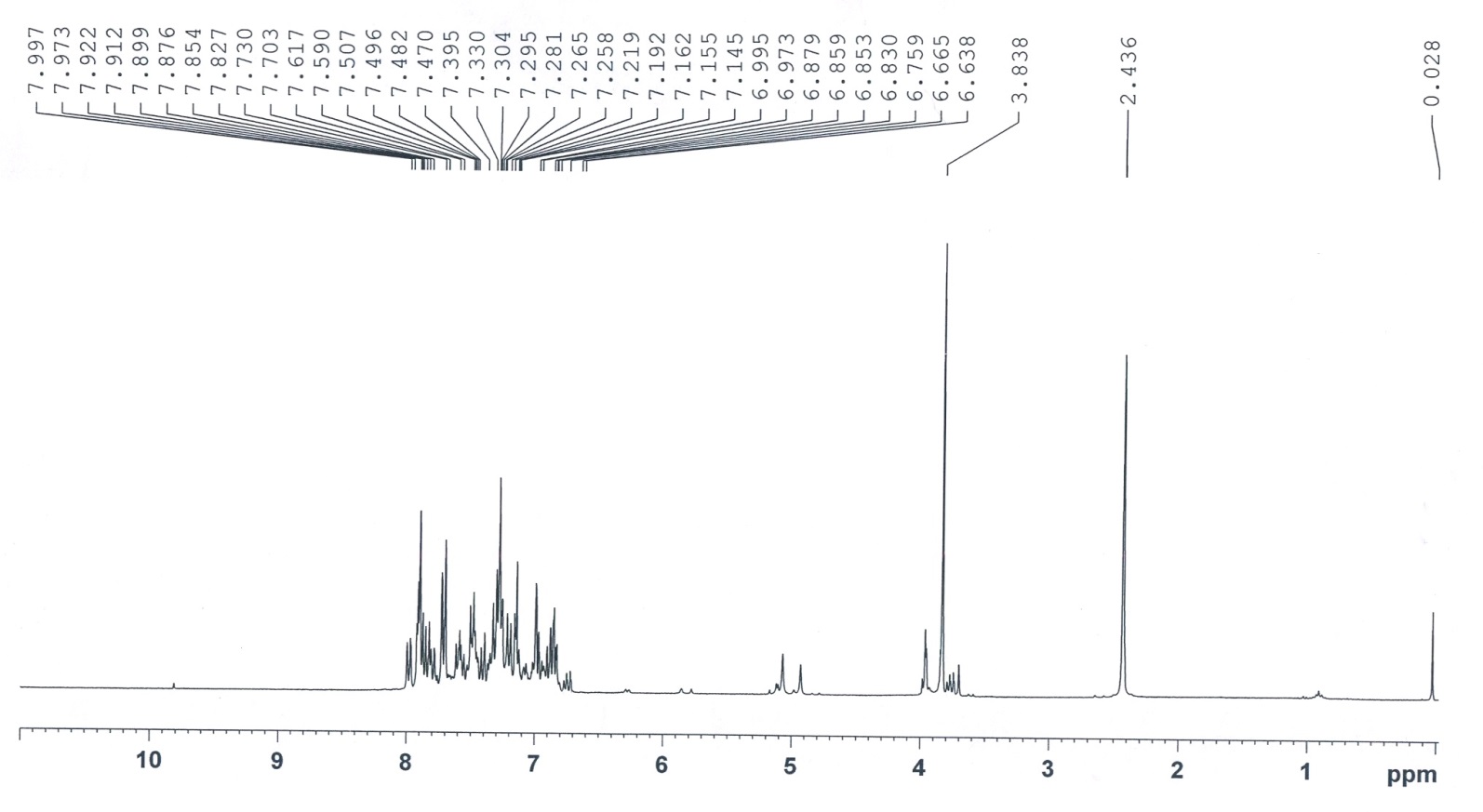

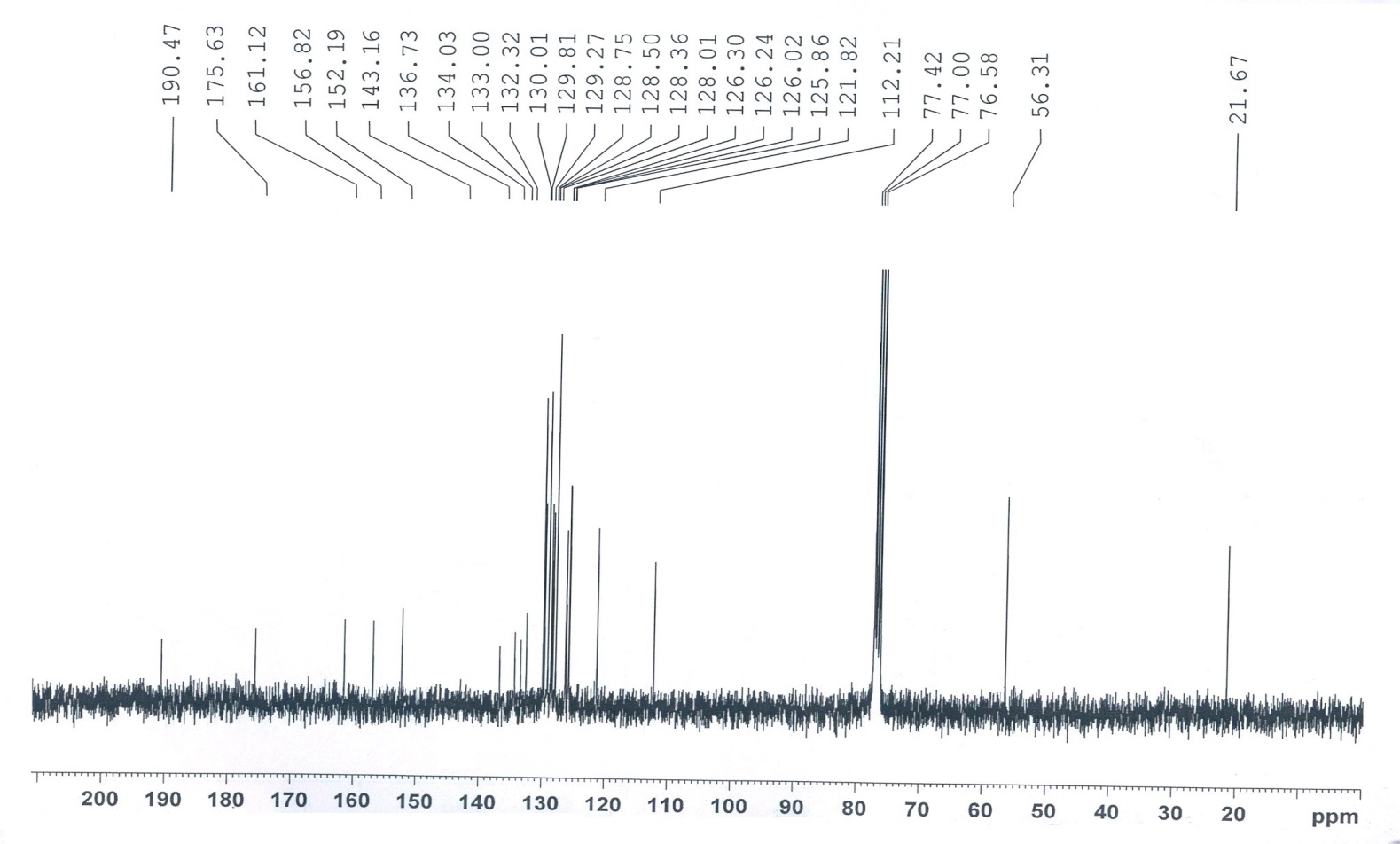


**SI Figure 22:** ^1^H and ^13^C-NMR spectra of compound **6i**


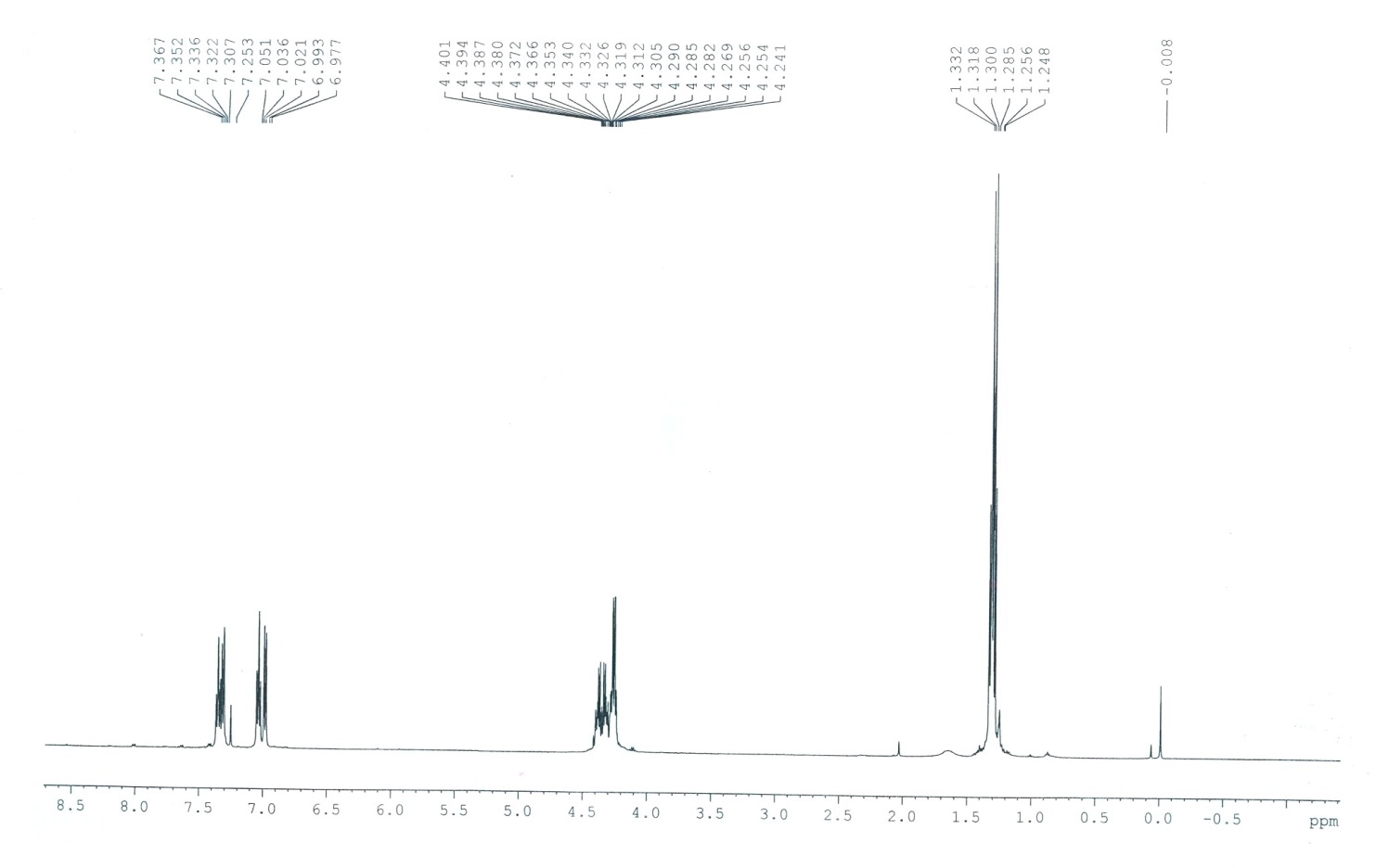

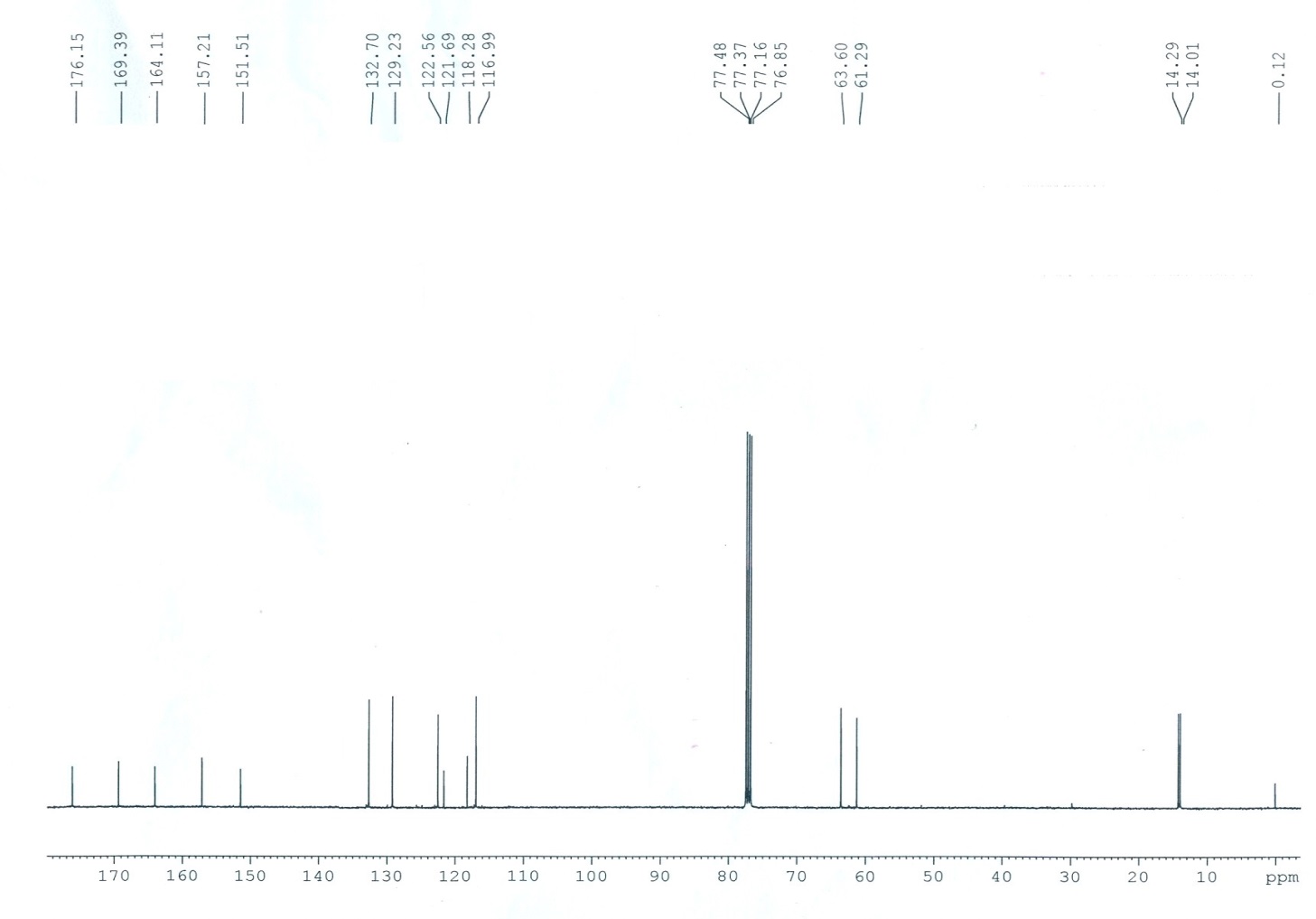


**SI Figure 23:** ^1^H and ^13^C-NMR spectra of compound **6j**


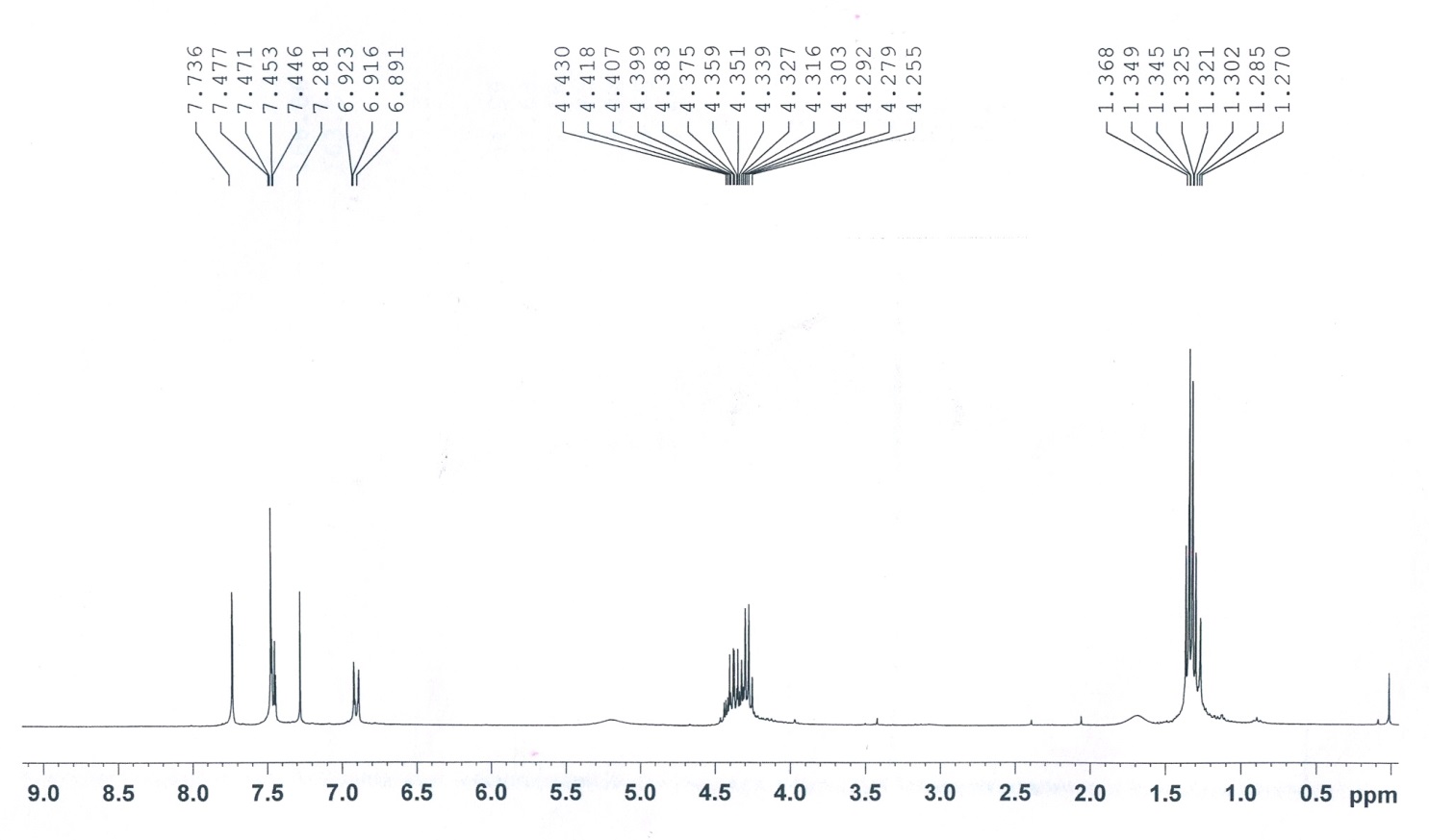

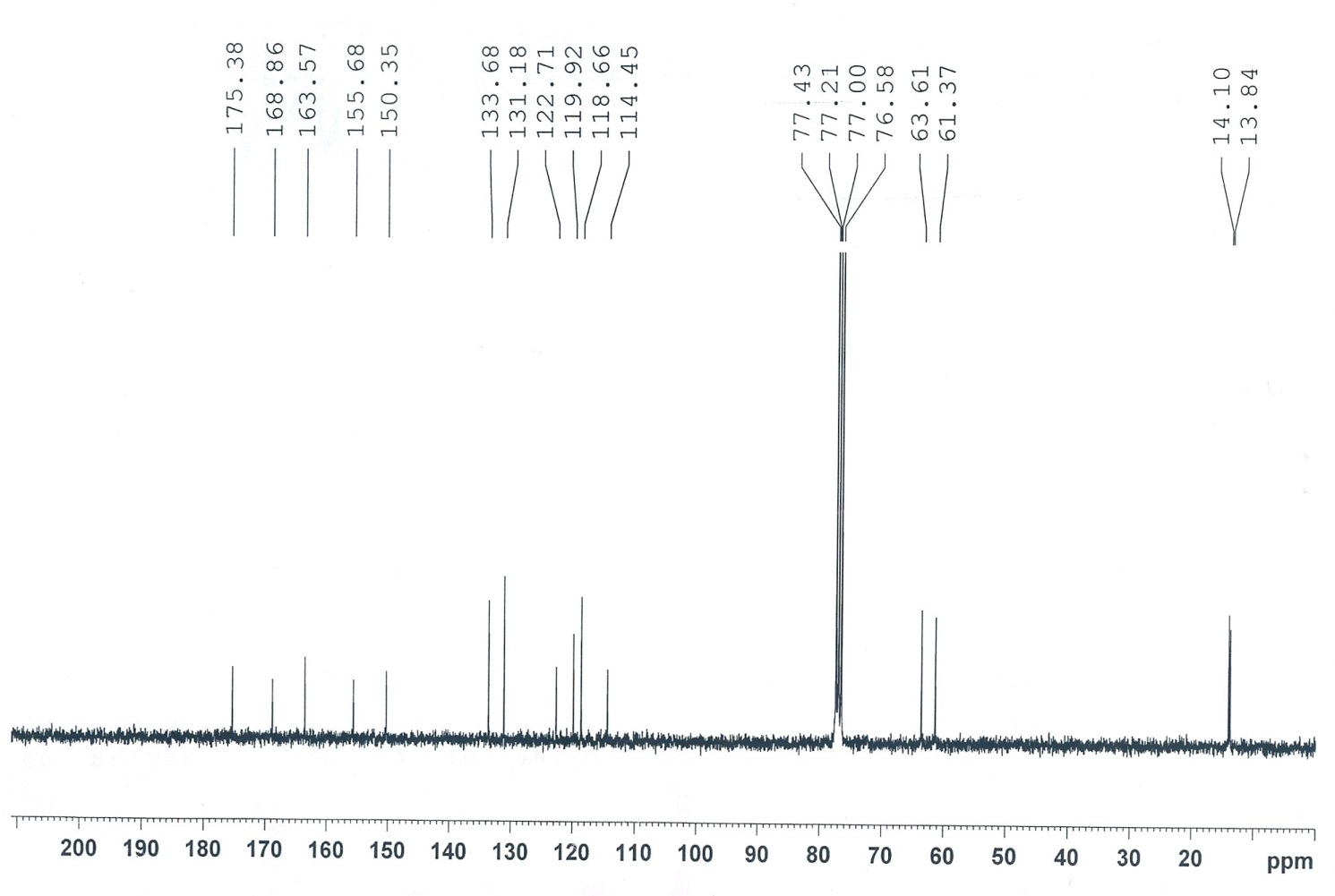


**SI Figure 24:** ^1^H and ^13^C-NMR spectra of compound **6k**

**
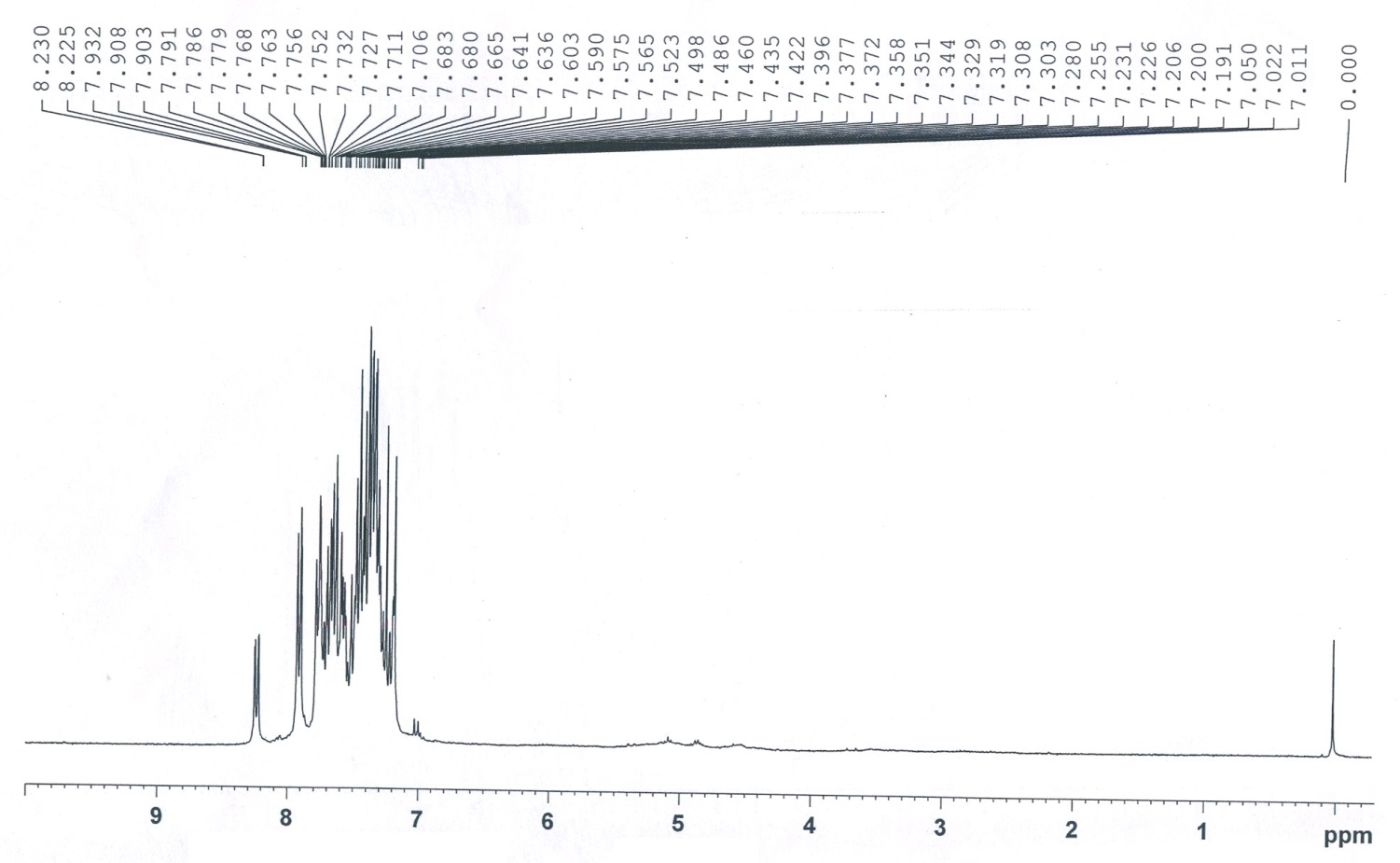
**

**
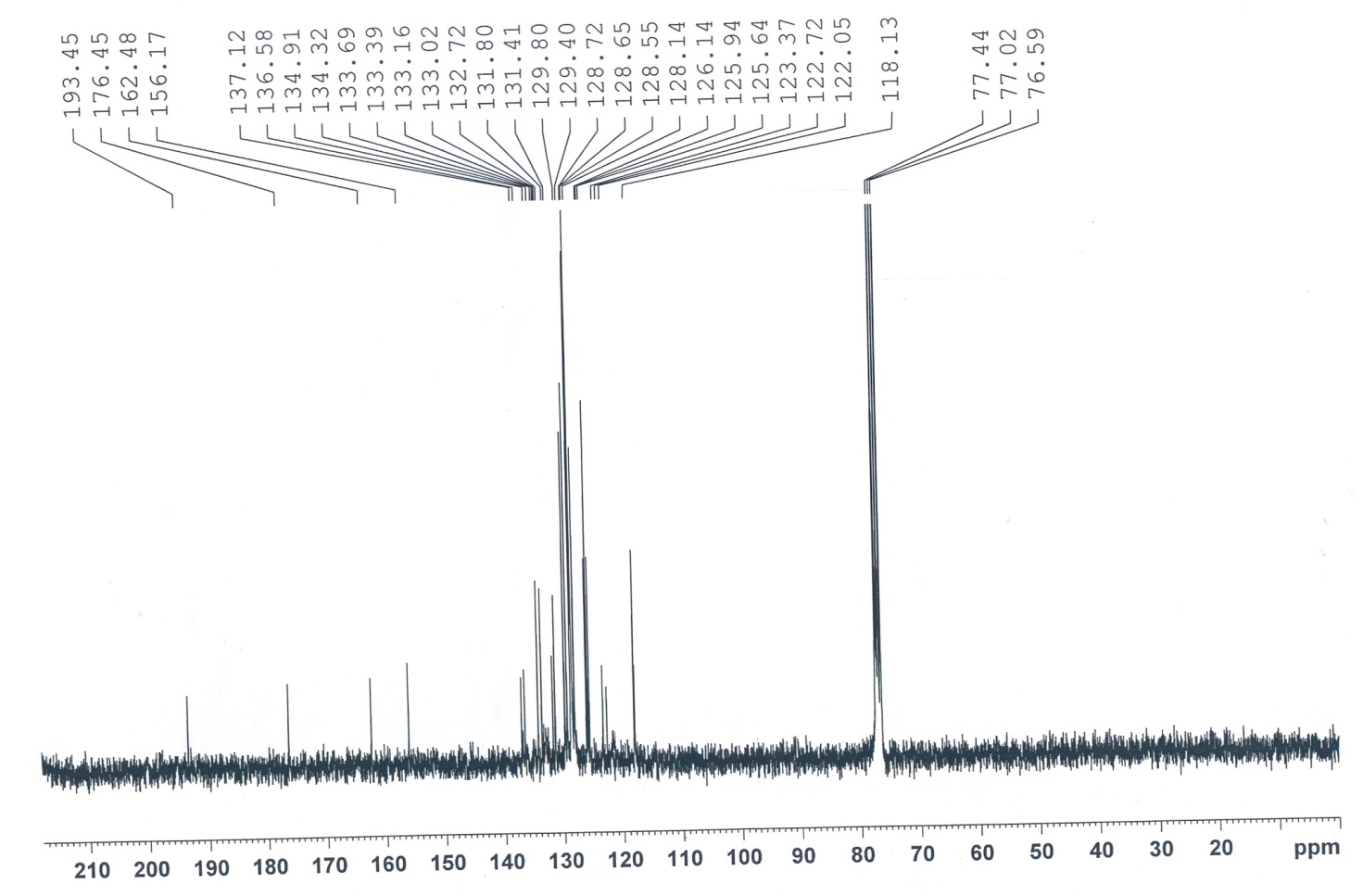
**

**SI Figure 25:** ^1^H and ^13^C-NMR spectra of compound **7a**


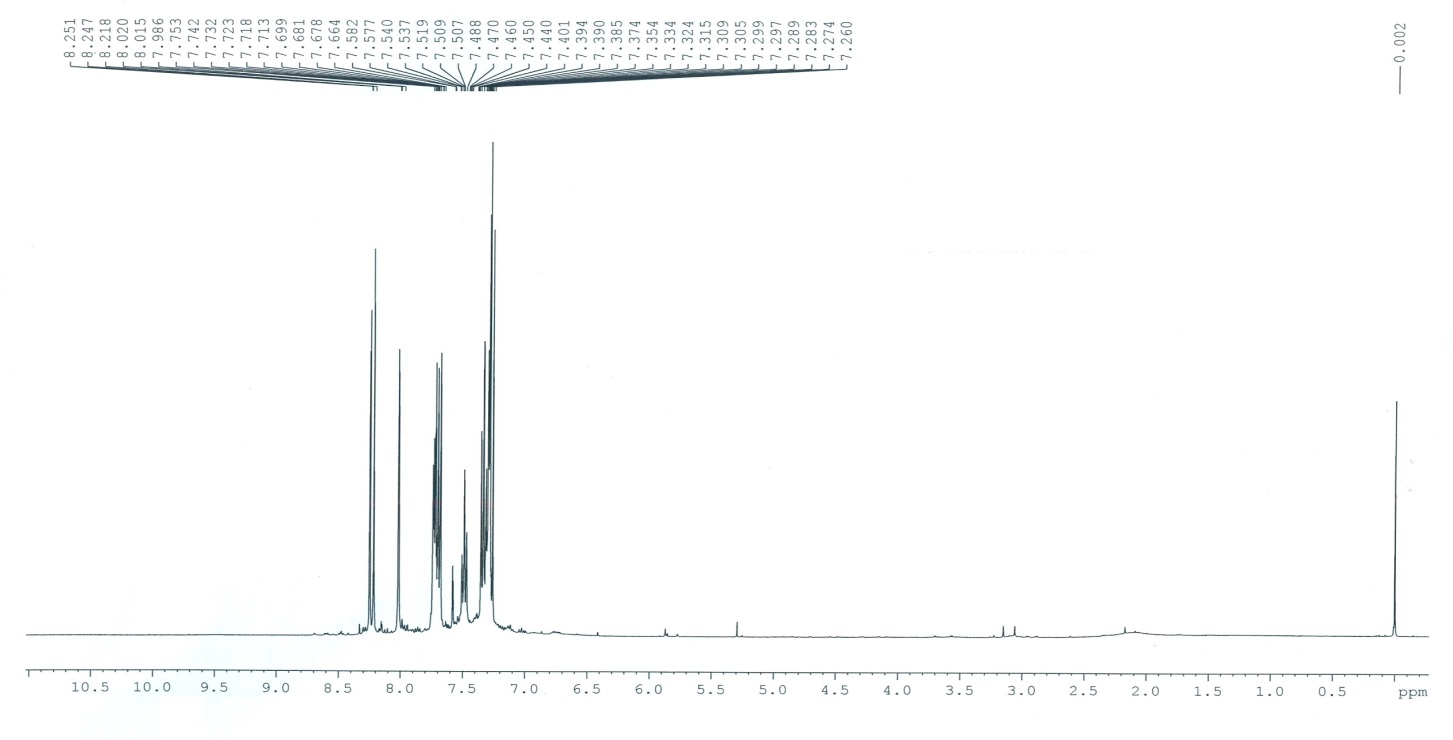

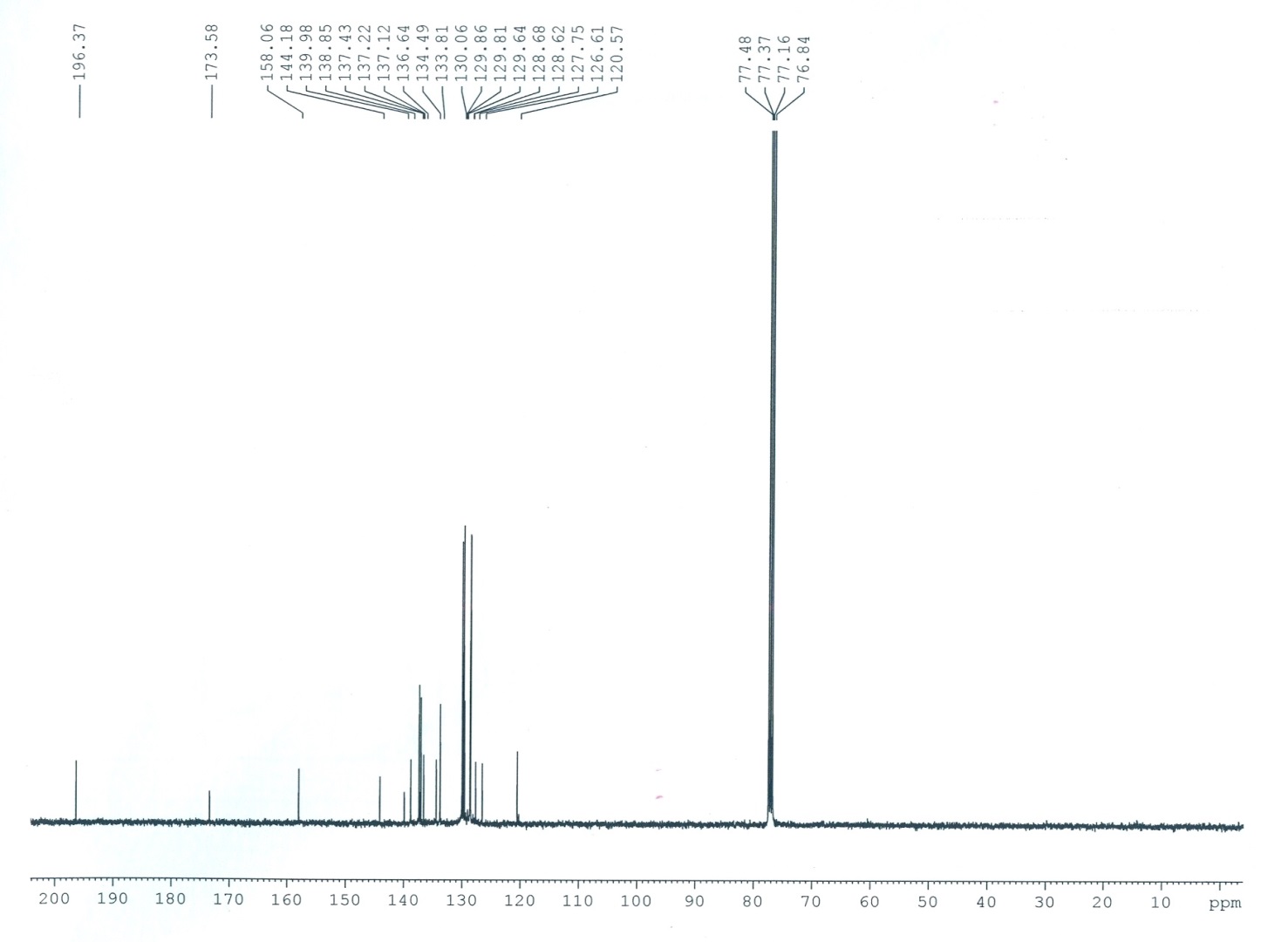


**SI Figure 26:** ^1^H and ^13^C-NMR spectra of compound **7b**


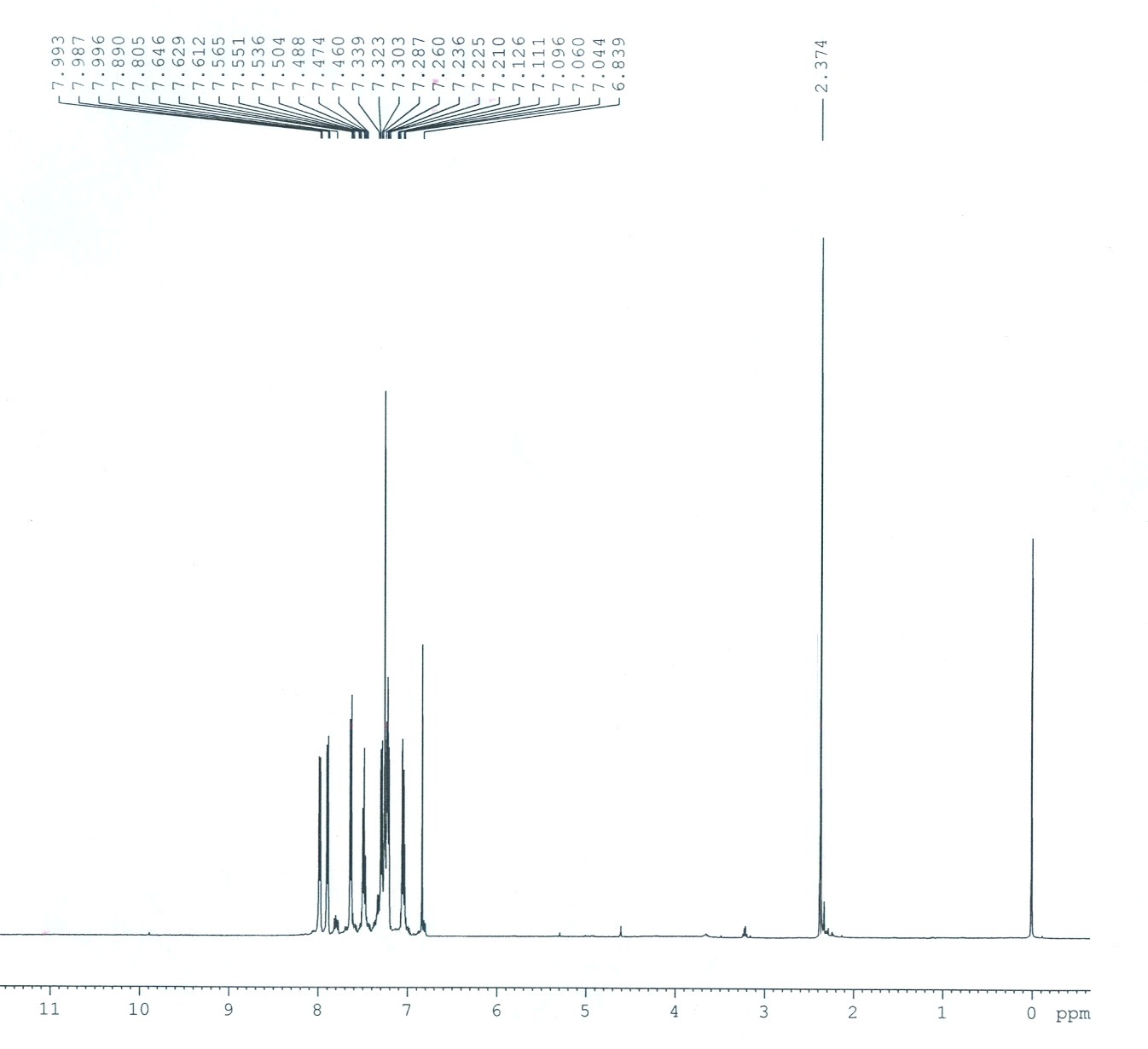

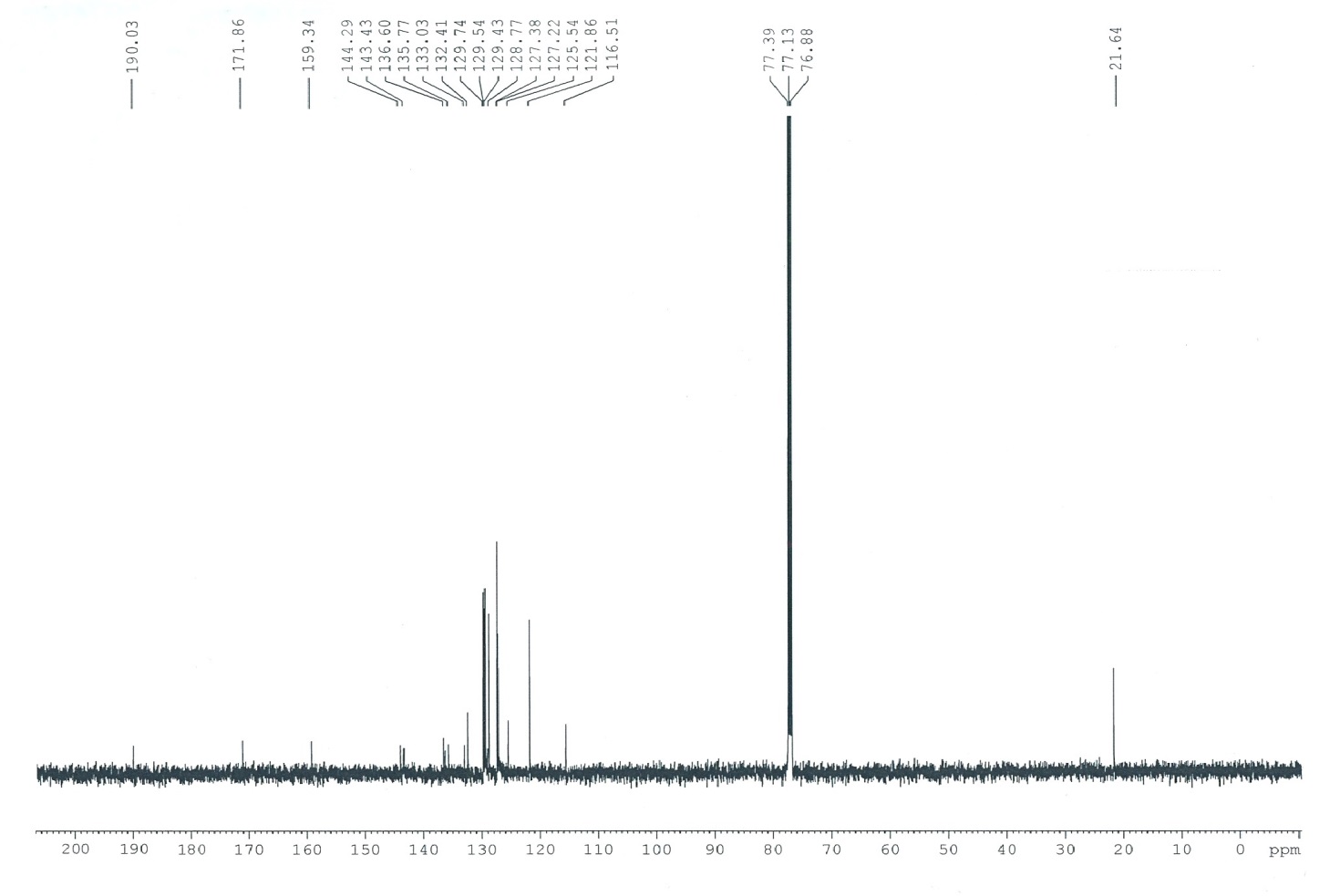


**SI Figure 27:** ^1^H and ^13^C-NMR spectra of compound **7c**


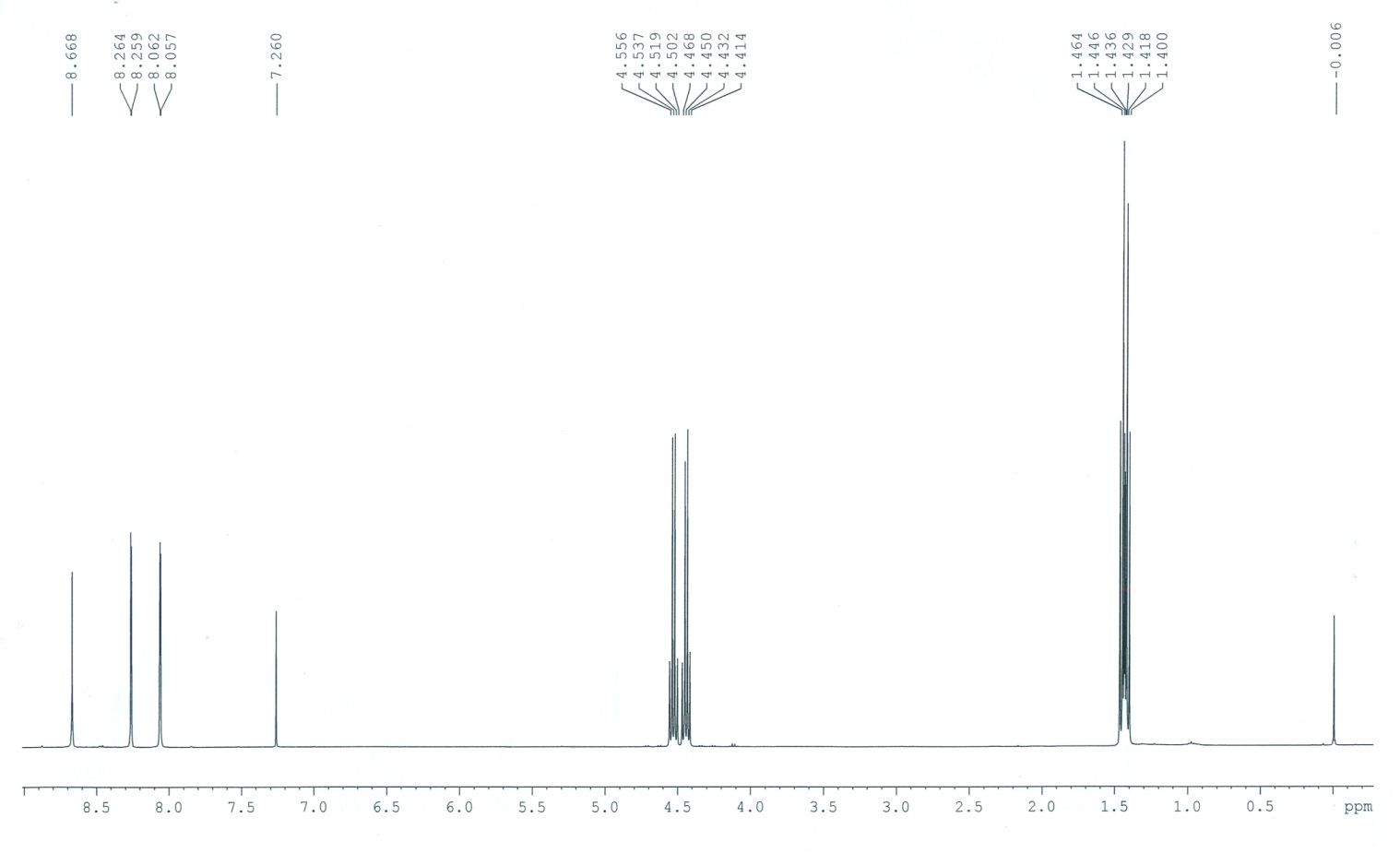

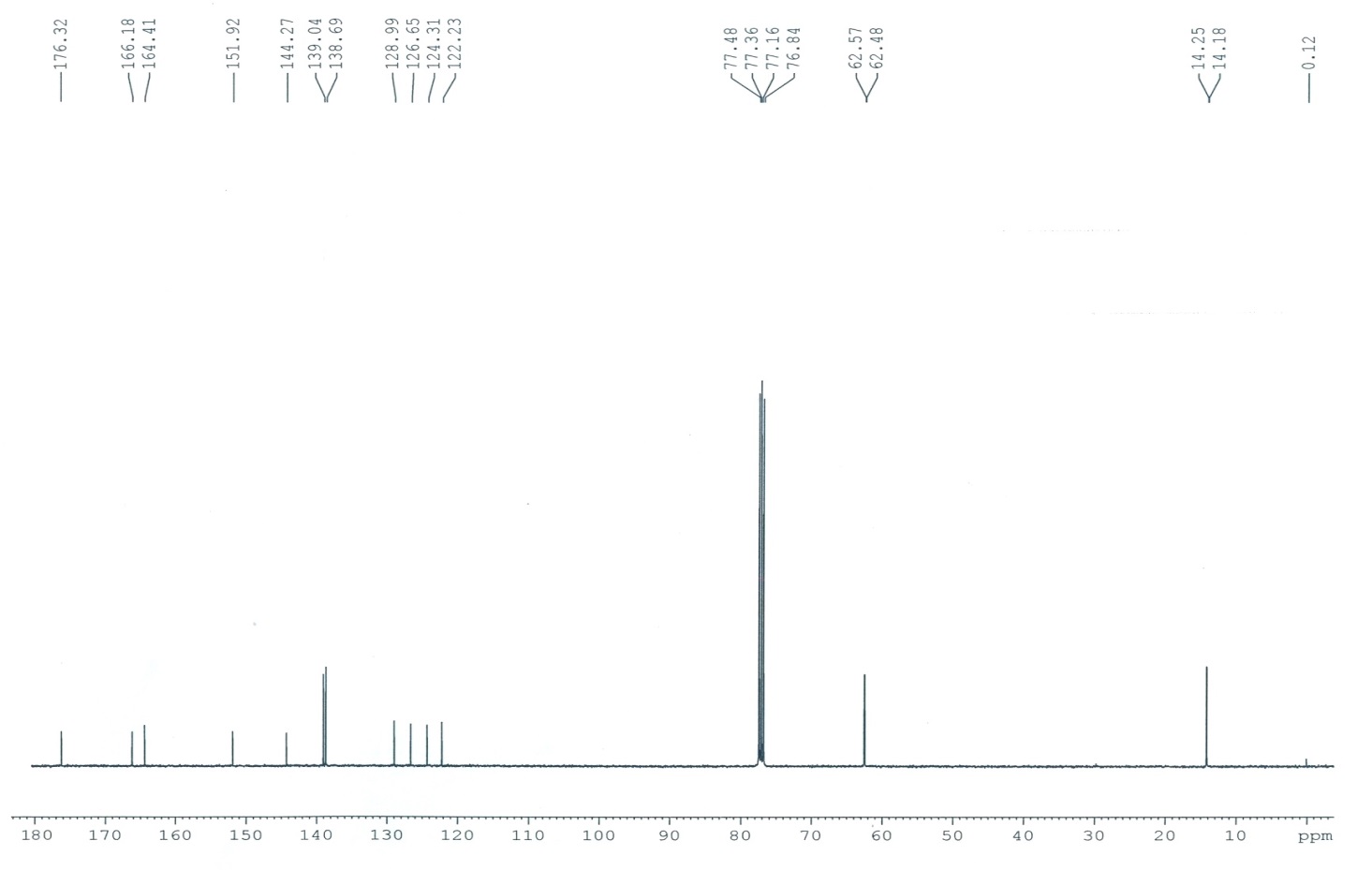


**SI Figure 28:** ^1^H and ^13^C-NMR spectra of compound **8a**


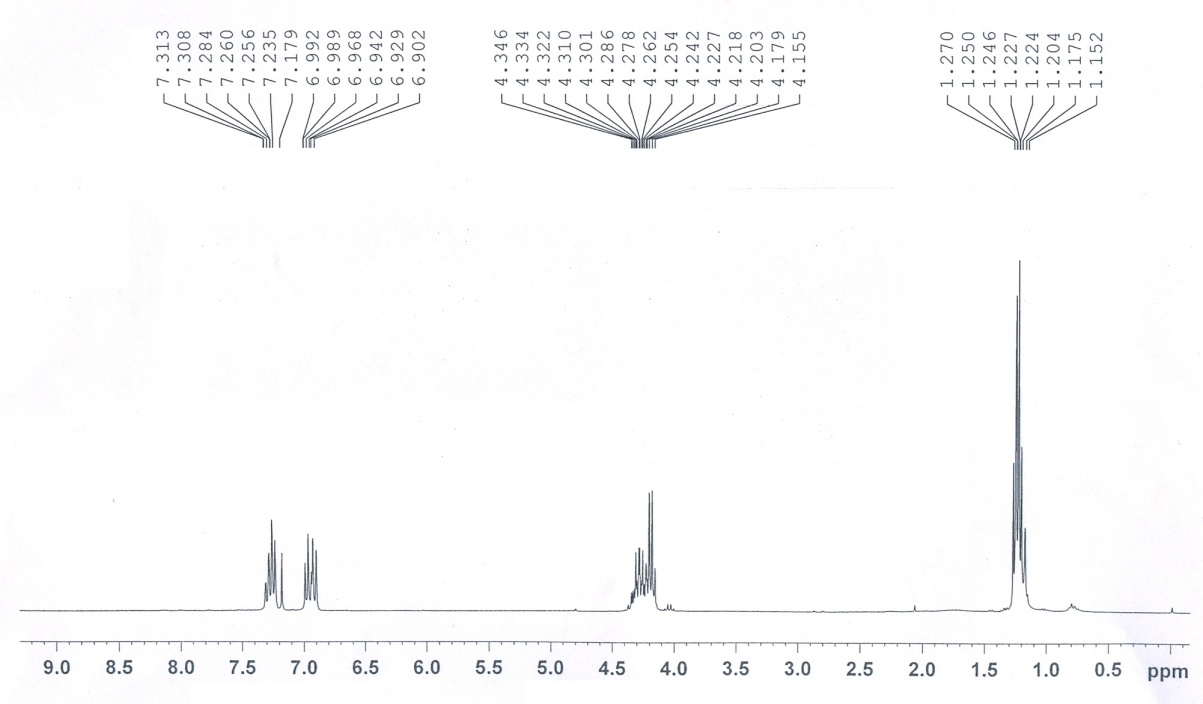

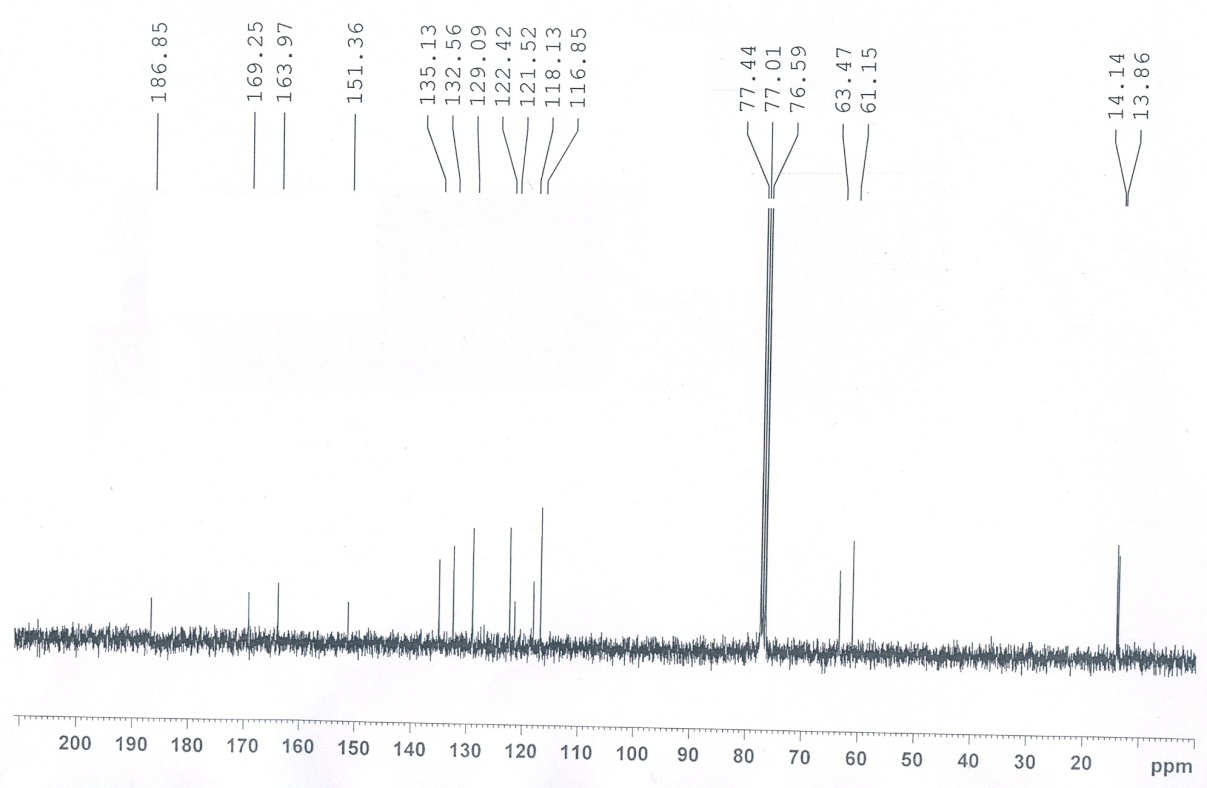


**SI Figure 29:** ^1^H and ^13^C-NMR spectra of compound **8b**


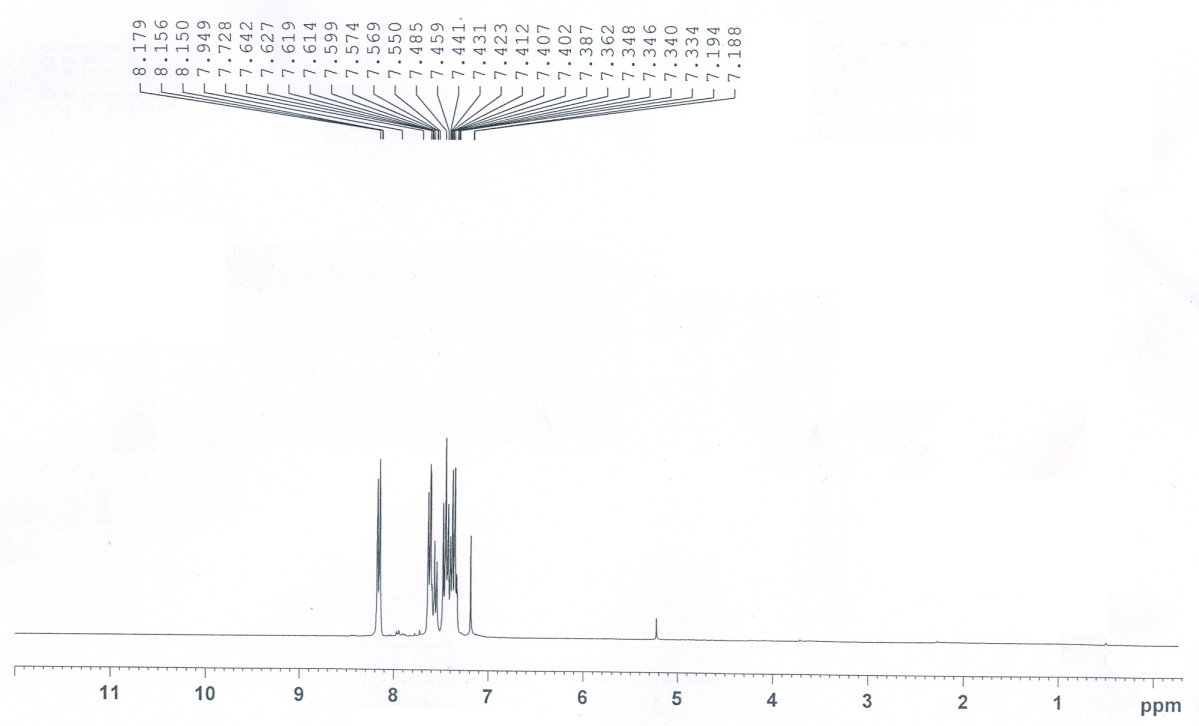

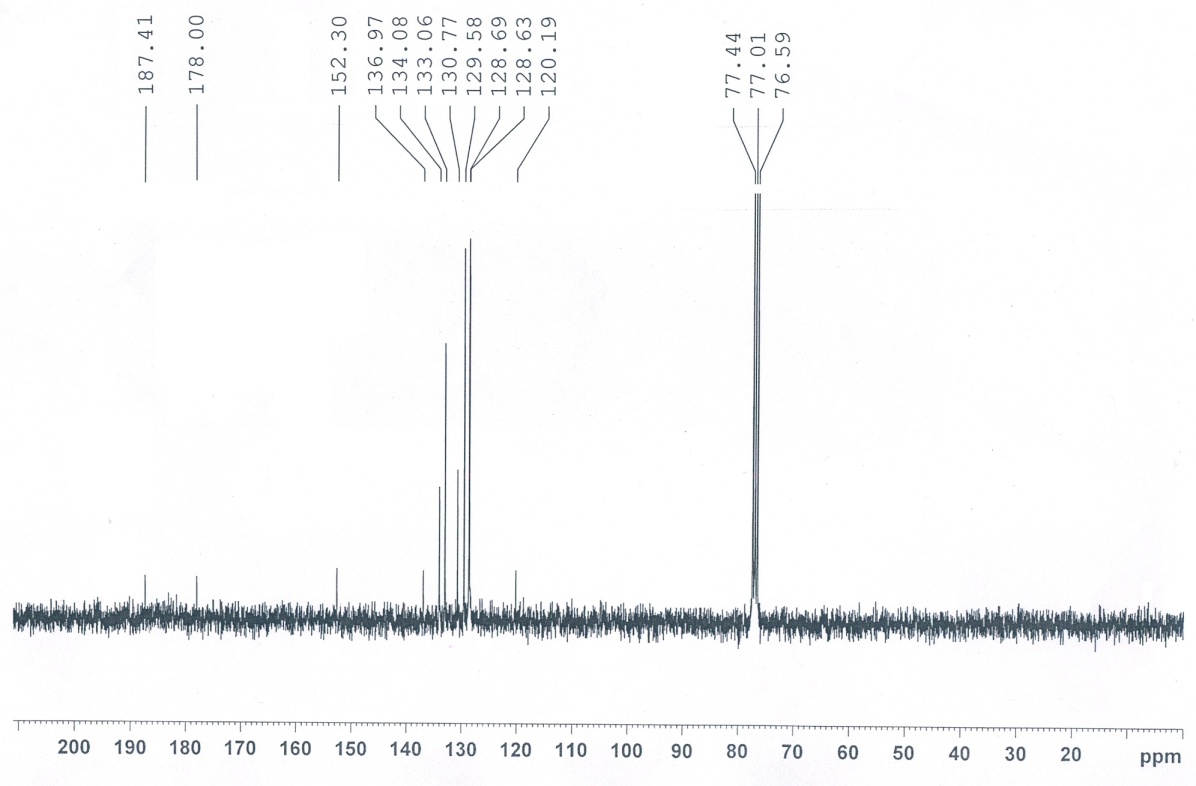


**SI Figure 30:** ^1^H and ^13^C-NMR spectra of compound **9a**


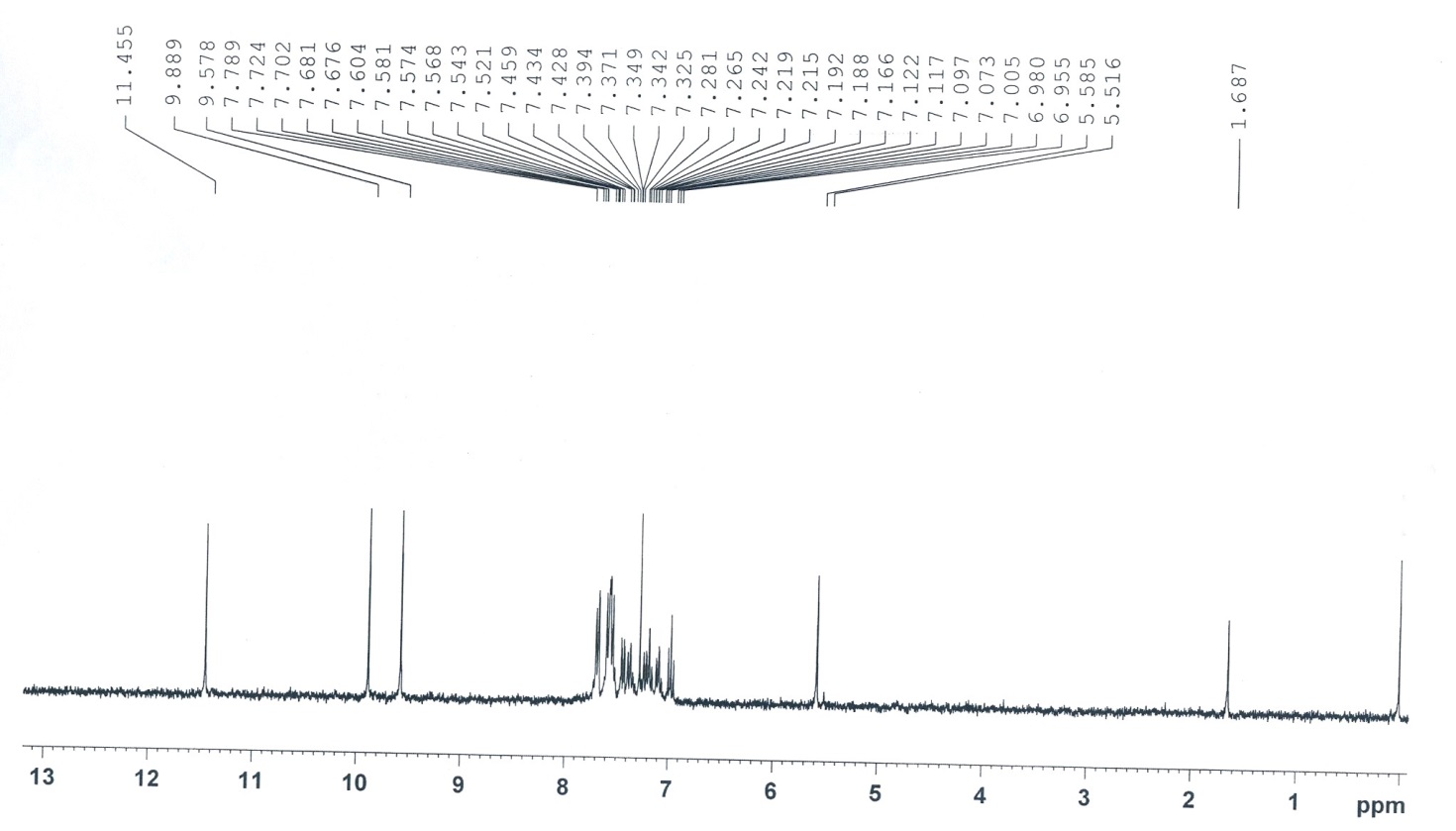

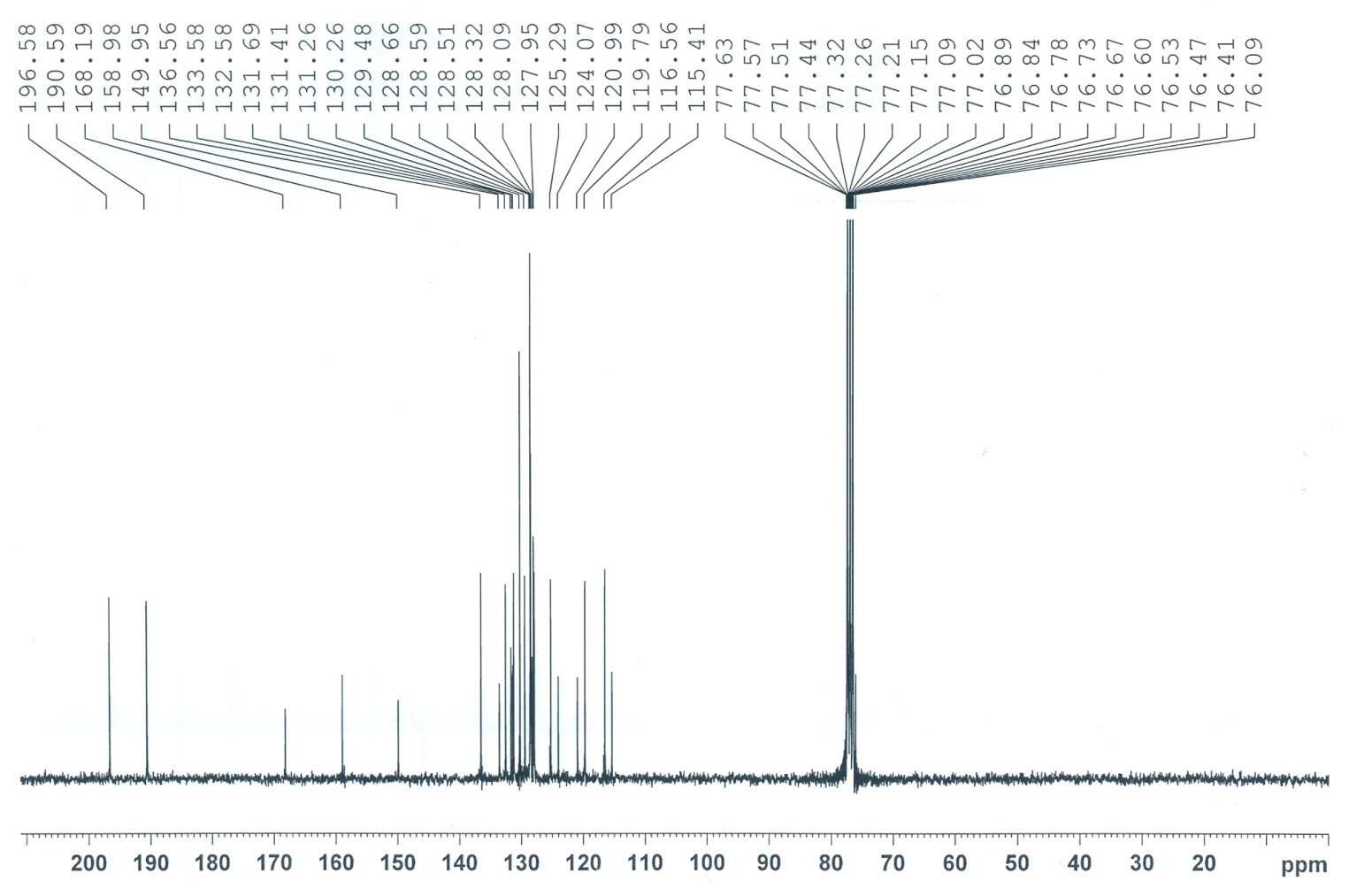


**SI Figure 31:** ^1^H and ^13^C-NMR spectra of compound **9b**


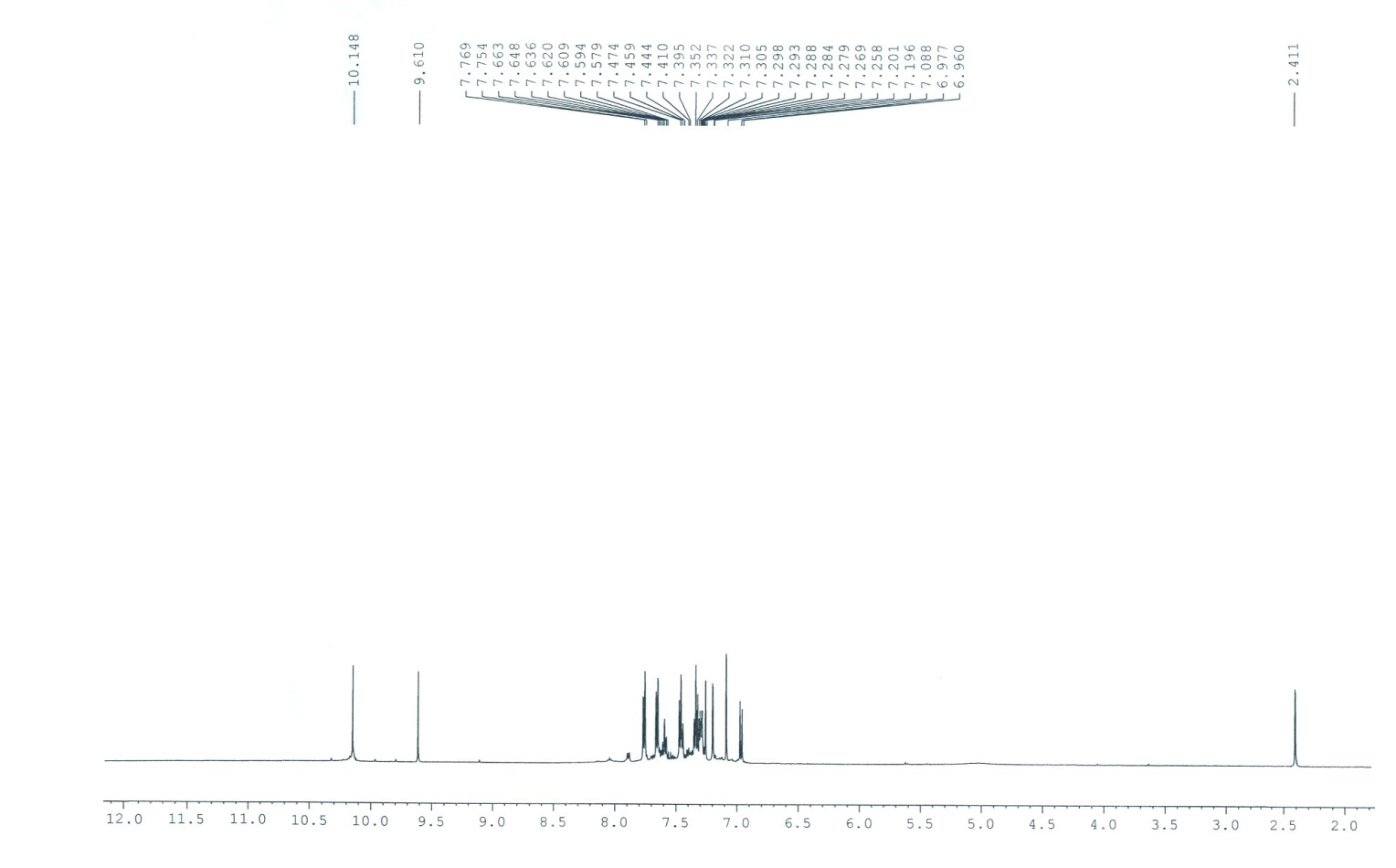

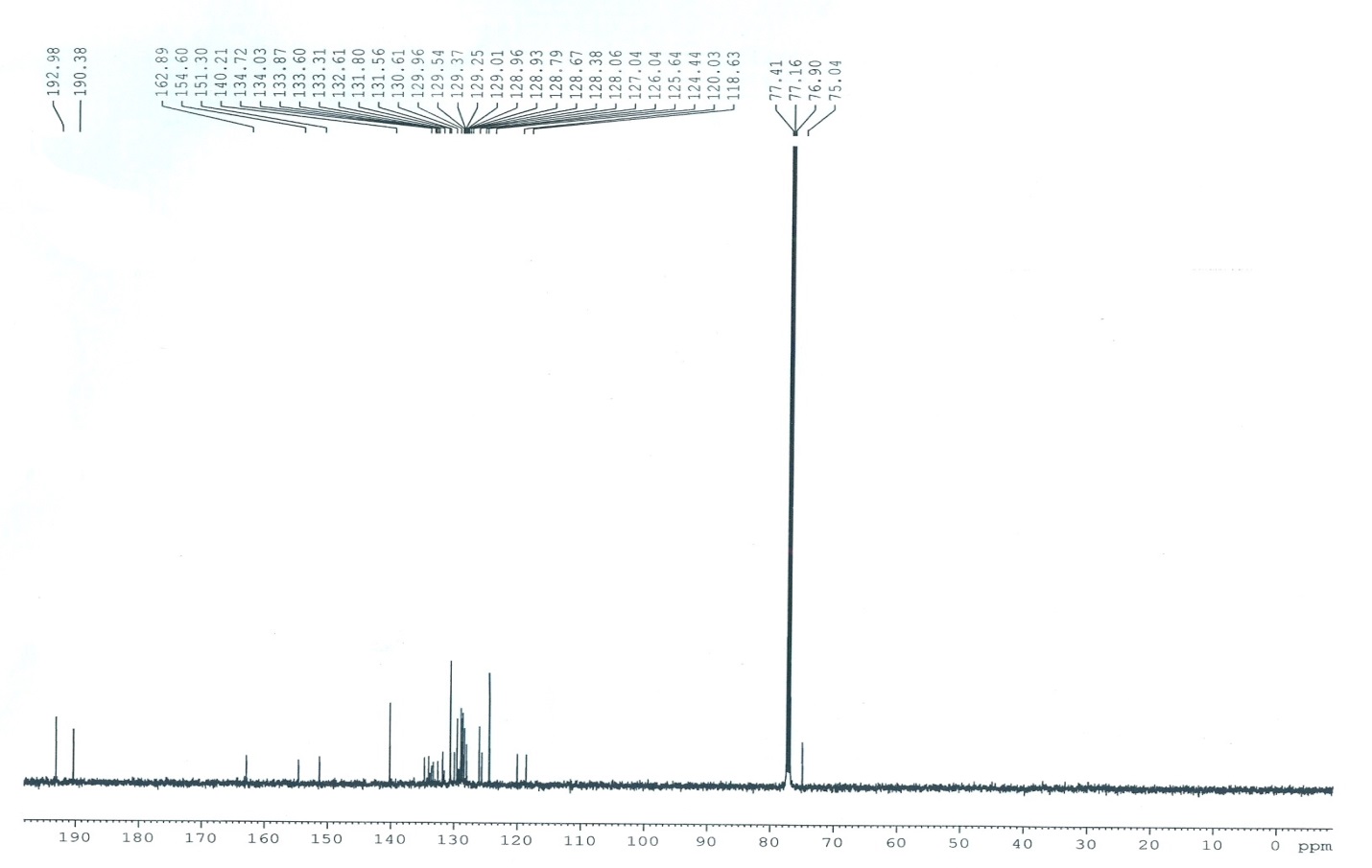


**SI Figure 32:** ^1^H and ^13^C-NMR spectra of compound **9c**


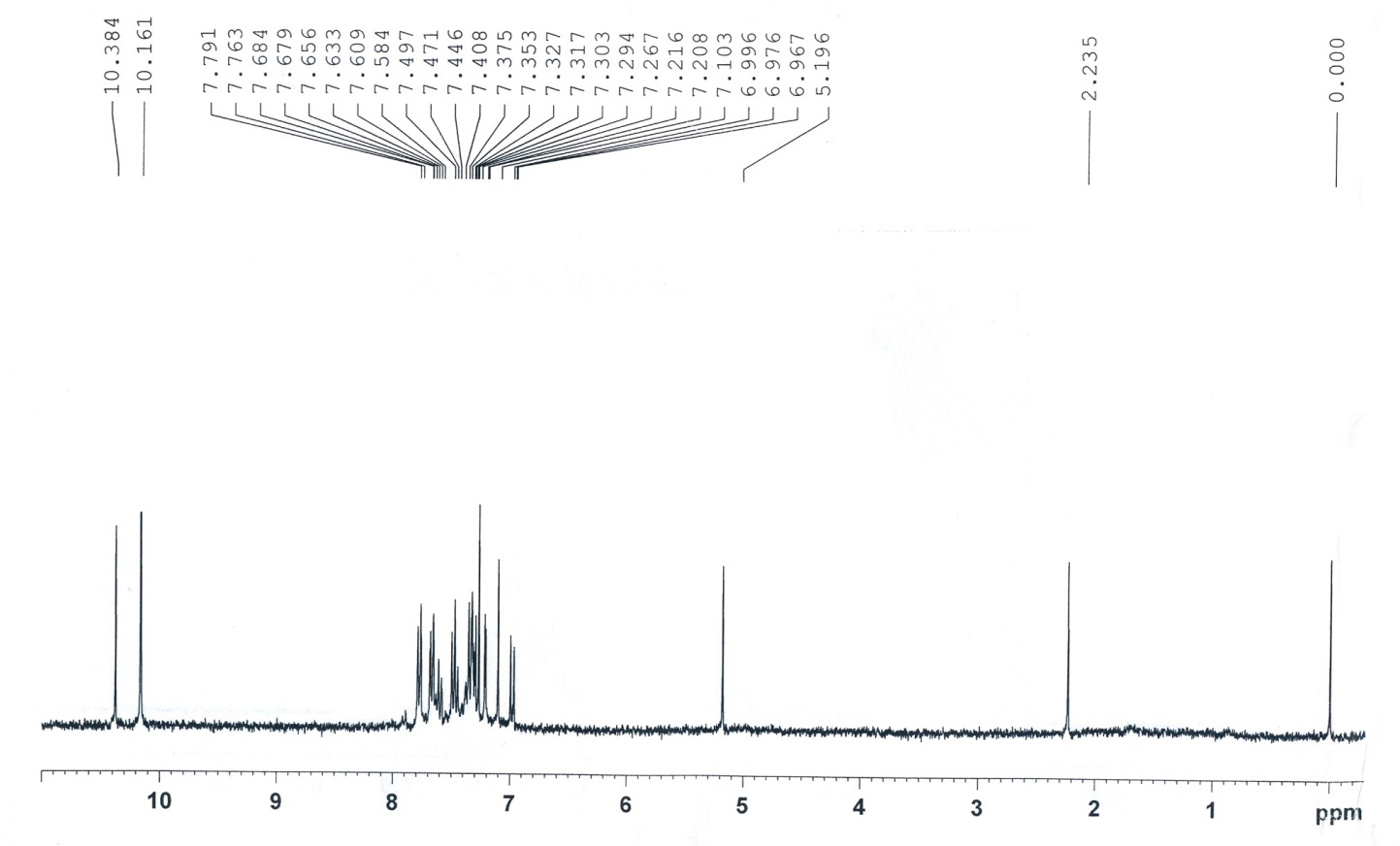

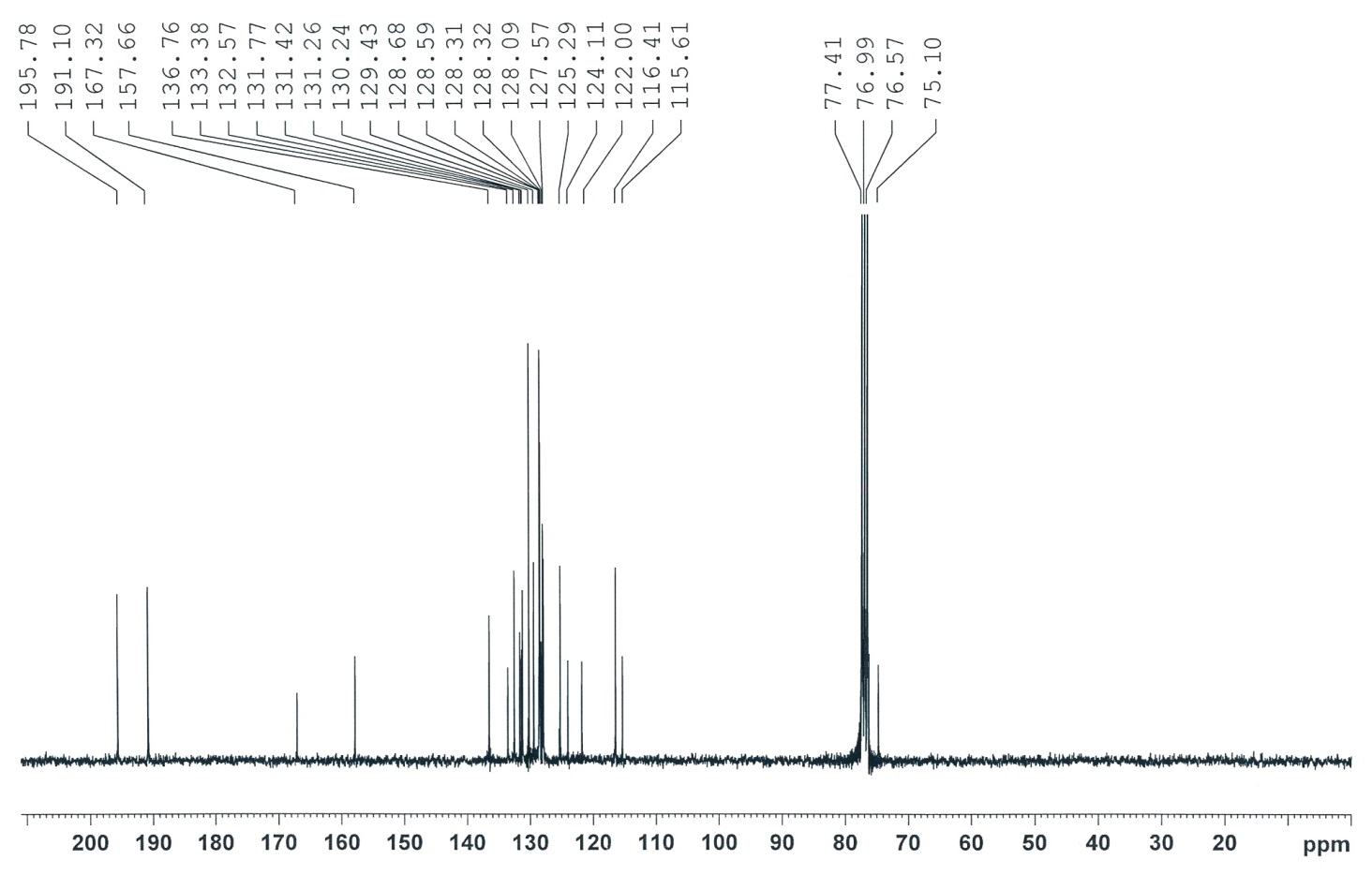

Supplement: Supplementary Information — Functionalised MnVI-nanoparticles: an advanced high-valent magnetic catalyst [file srep08636-s1.docx]
